# Cyclooxygenase-Inhibiting Platinum(IV) Prodrugs with Potent Anticancer Activity

**DOI:** 10.3390/pharmaceutics14040787

**Published:** 2022-04-03

**Authors:** Aleen Khoury, Jennette A. Sakoff, Jayne Gilbert, Kieran F. Scott, Shawan Karan, Christopher P. Gordon, Janice R. Aldrich-Wright

**Affiliations:** 1 School of Science, Western Sydney University, Locked Bag 1797, Penrith South, NSW 2751, Australia; aleen.khoury@westernsydney.edu.au (A.K.); shawan.karan@westernsydney.edu.au (S.K.); c.gordon@westernsydney.edu.au (C.P.G.); 2 Calvary Mater Hospital, Waratah, NSW 2298, Australia; jennette.sakoff@newcastle.edu.au (J.A.S.); jayne.gilbert@newcastle.edu.au (J.G.); 3 School of Medicine, Western Sydney University, Locked Bag 1797, Penrith South, NSW 2751, Australia; kieran.scott@westernsydney.edu.au; 4 Ingham Institute, 1 Campbell Street, Liverpool, NSW 2170, Australia

**Keywords:** platinum(IV), prodrugs, cancer, cyclooxygenase, 56MESS, cytotoxicity, nanomolar, lipophilicity, NSAIDS

## Abstract

Platinum(IV) prodrugs of the [Pt(P_L_)(A_L_)(COXi)(OH)]^2+^ type scaffold (where P_L_ is 1,10-phenanthroline or 5,6-dimethyl-1,10-phenanthroline, A_L_ is 1*S*,2*S*-diaminocyclohexane, and COXi is a COX inhibitor, either indomethacin or aspirin) were synthesised and characterised, and their biological activity was explored. MTT assays showed that these complexes exhibit outstanding activity against a range of cancer cell lines, and nanomolar activities were observed. The most potent complex, **4**, exhibited a GI_50_ of 3 nM in the Du145 prostate cancer cell line and was observed to display a 1614-fold increased activity against the HT29 colon cancer cell line relative to cisplatin. ICP-MS studies showed a linear correlation between increased cellular accumulation of the complexes and increased cytotoxicity, while an enzyme immunoassay showed that **1** and **2** inhibited COX-2 at 14 and 1.4 µM, respectively, which is comparable to the inhibition exhibited by indomethacin. These results suggest that while the cytotoxicity of prodrugs **1**–**4** was influenced by cellular uptake, it was not entirely dependent on either COX inhibition or lipophilicity.

## 1. Introduction

**Figure 1:**
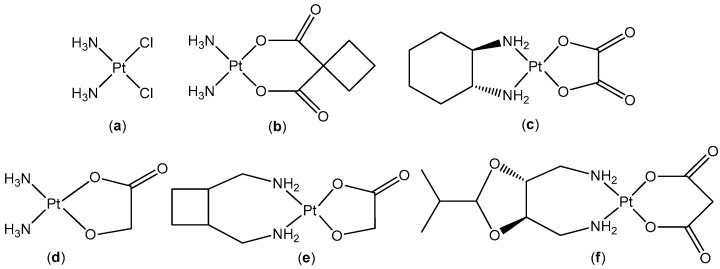
The chemical structures of: cisplatin (**a**); carboplatin (**b**); oxaliplatin (**c**); nedaplatin (**d**); lobaplatin (**e**); and heptaplatin (**f**).

Chemotherapy is the most prominent method in cancer therapy, with platinum(II)-based drugs used in ~50% of all treatments [1]. The first platinum(II)-based drug to gain FDA approval was cisplatin in 1978 (Figure 1) [2,3]. While cisplatin is routinely deployed to treat a variety of cancers, its efficacy is mitigated by severe toxic side-effects as well as intrinsic or acquired drug resistance [4,5]. Nevertheless, following the discovery of the antineoplastic activity of cisplatin, thousands of cisplatin analogues have been developed; carboplatin and oxaliplatin are approved for clinical use worldwide, while nedaplatin, lobaplatin, and heptaplatin (Figure 1) have gained approval for regional use, specifically in Asia [6,7]. These cisplatin analogues demonstrate improvements in certain aspects of chemotherapy; however, because they are structurally similar, they exhibit similar mechanisms of action to cisplatin, and therefore similar drawbacks [5,8]. As cancer continues to be a leading cause of death worldwide, the continued need for improved treatments is crucial.

Cisplatin and its analogues exert their cytotoxicity by irreversibly binding to DNA, forming inter- and intra-strand adducts, distorting the double helix, and ultimately resulting in apoptosis [9]. However, in a somewhat counterintuitive fashion, despite forming fewer DNA adducts than cisplatin, oxaliplatin exhibits comparable cytotoxicity [10,11]. Its ability to perturb ribosome biogenesis and promote immunogenic cell death by facilitating a T cell-dependent immune response is considered a significant contributor to cell death [12,13]. Consequently, oxaliplatin exhibits improved activity in cisplatin-resistant cell lines. Accordingly, the design and development of platinum complexes that demonstrate dissimilar mechanisms of action may circumvent the limitations that challenge current chemotherapeutics.

A group of platinum(II) complexes that have displayed promising results are of the form [Pt(P_L_)(A_L_)]^2+^, where P_L_ is a polyaromatic ligand such as 1,10-phenanthroline (Phen) or 5,6-dimethyl-1,10-phenanthroline (56Me_2_Phen) and A_L_ is a chiral ancillary ligand such as 1*S*,2*S*-diaminocyclohexane (SSDACH) [14,15]. These complexes exhibit a multimodal mechanism of action through which they have been observed to alter cytoskeletal phenotypes, damage nuclear DNA, reduce mitochondrial membrane potential, and induce epigenetic processes [16]. This class of complexes exhibit nanomolar in vitro IC_50_ values across numerous cancer cell lines; however, this superior activity has not entirely translated in vivo due to poor pharmacokinetics [17]. To improve this, they were subsequently oxidised to platinum(IV) ([Pt(P_L_)(A_L_)(OH)_2_]^2+^) with two hydroxido axial ligands, which demonstrated enhanced activity in vivo [18,19].

Platinum(IV) complexes are endowed with a low-spin d^6^ octahedral geometry, making them more kinetically stable; this reduces the probability of them reacting with extracellular biomolecules, which decreases the likelihood of side-effects [20,21,22]. The two additional coordination sites provide the potential for different groups to be conjugated, which can improve the pharmacological properties of the complex, including its lipophilicity, selectivity, bioactivity and reduction kinetics [23,24,25,26,27]. Platinum(IV) prodrugs are reduced to platinum(II) by intra-/extracellular reducing agents before interacting with their biological targets within the cell [28,29]. The advantage of prodrugs is that, in contrast to the various delivery modes of drugs that are used in traditional combination therapy, they can deliver multiple drug moieties in a single pharmacokinetic dose [7].

The unique properties of platinum(IV) complexes have spawned the development of prodrugs of cisplatin encompassing different enzyme inhibitors [6,7,30]. Several prodrugs of cisplatin incorporating various non-steroidal anti-inflammatory drugs (NSAIDs) have been reported [31,32,33,34,35]. NSAIDs target cyclooxygenase (COX) enzymes, which exist in two isoforms, COX-1 and COX-2. COX-1 is expressed in most tissues, while COX-2 is linked to inflammatory reactions and is overexpressed in many cancers, most prominently in prostate, breast, colon, and lung cancers [36]. Targeting COX has gained significant interest, as ~20% of cancers manifest chronic inflammation [36,37]. Previous prodrugs comprising of cisplatin with NSAIDs have demonstrated improved cytotoxicity. In certain cases this was due to an enhanced anti-inflammatory response, suggesting a synergistic effect of COX inhibition; in other cases the improved activity was due to enhanced lipophilicity, resulting in enhanced cellular accumulation [31,32,33,34,38,39].

**Figure 2:**
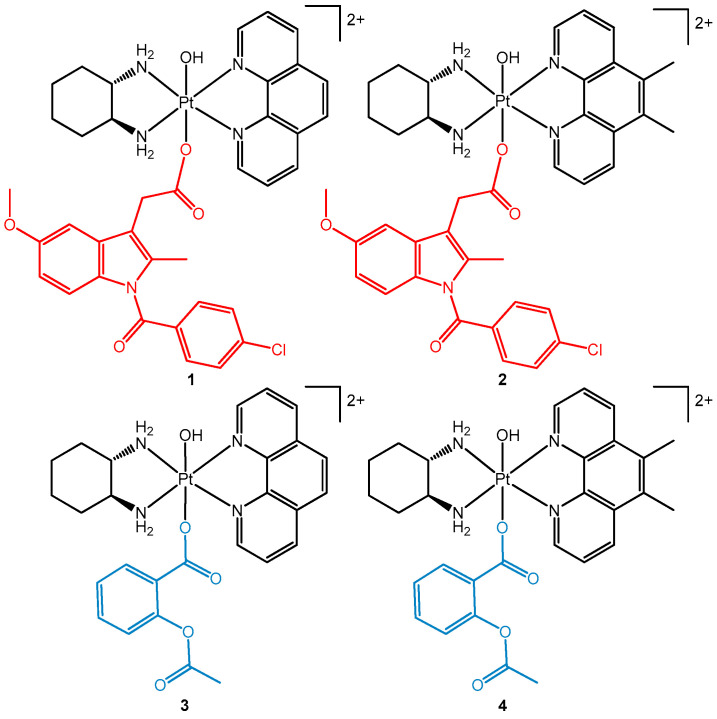
The chemical structures of multi-functional platinum(IV) prodrugs comprising of the COX inhibitors indomethacin (**1** and **2**) and aspirin (**3** and **4**) in the axial positions.

We present here, for the first time, a group of platinum(IV) prodrugs of the [Pt(P_L_)(A_L_)(COXi)(OH)]^2+^ type, where COXi is a COX inhibitor, either indomethacin or aspirin (Figure 2). These prodrugs were developed in an attempt to further understand the influence of NSAIDS with platinum-based chemotherapeutics and to improve the activity of [Pt(P_L_)(A_L_)(OH)_2_]^2+^ type complexes. The platinum(IV) complexes were characterised using several biophysical techniques, and their lipophilicity was studied using RP-HPLC. MTT assays were performed in order to determine their cytotoxicity against a panel of malignant cell lines and one non-malignant cell line. To rationalise the cytotoxicity of these complexes, their capacity to inhibit COX-2 was explored, and ICP-MS was employed to determine their cellular accumulation in two different cell lines.

## 2. Materials and Methods

### 2.1. Materials

All reagents were used as received unless otherwise specified, all organic solvents utilised were of analytical grade, and MilliQ^TM^ water (Merck, Melbourne, Australia) was used. Potassium tetrachloroplatinate (K_2_PtCl_4_) was acquired from Precious Metals Online. 1*S*,2*S*-Diaminocyclohexane (SSDACH, 98%), 1,10-phenanthroline (Phen), 5,6-dimethyl-1,10-phenanthroline (56Me_2_Phen), silver nitrate, *N*,*N*′-dicyclohexylcarbodiimide (DCC), *N*-(3-Dimethylaminopropyl)-*N*’-ethylcarbodiimide hydrochloride (EDC-HCl), *N*-hydroxysuccinimide (NHS), indomethacin and aspirin were purchased from Sigma-Aldrich, Sydney, NSW. Sep-Pak^®^ C18 columns were obtained from Waters, Australia. Methanol, diethyl ether, ethyl acetate, dichloromethane (DCM), dimethyl formamide (DMF), dimethyl sulfoxide (DMSO) and acetonitrile (ACN) were acquired from Chem-Supply, Adelaide, Australia. Hydrogen peroxide (H_2_O_2_) (30%) was purchased from VWR chemicals, Philadelphia, United States. Potassium iodide (KI) was obtained from Merck, Melbourne, Australia. Ultra-pure HNO_3_ (69%) was acquired from Choice Analytical, Sydney, Australia. The certified reference standard that was used for the ICP-MS experiments was purchased from High-Purity Standards, North Charleston, United States. The deuterated solvents deuterium oxide (D_2_O, 99.9%) and d_6_-dimethyl sulfoxide (DMSO-d_6_, 99.9%) were obtained from Novachem, Melbourne, Australia.

### 2.2. Synthesis of [Pt(P_L_)(SSDACH)]Cl_2_ (**PHENSS(II)** and **56MESS(II)**) and [Pt(P_L_)(SSDACH)(OH)_2_](NO_3_)_2_ (**PHENSS(IV)** and **56MESS(IV)**) (where P_L_ = Phen or 56Me_2_Phen)

The synthesis of platinum(II) and di-hydroxido platinum(IV) complexes were carried out following previously published methods with no modifications [18,25].

### 2.3. Synthesis of Indomethacin N-hydroxysuccinimide (Indomethacin-NHS)

Indomethacin (0.3 g, 0.84 mmol), NHS (0.106 g, 0.92 mmol) and EDC-HCl (0.241 g, 1.26 mmol) were dissolved in anhydrous DMF (8 mL). This mixture was stirred overnight at room temperature in the dark. H_2_O (~20 mL) was added to the solution to form a white precipitate which was collected using vacuum filtration and washed with H_2_O two times. The indomethacin–NHS ester was either used immediately in the next synthetic step or stored at −20 °C. Yield (291.1 mg, 76%). ^1^H NMR (400 MHz, DMSO-d_6_) δ (ppm): 7.67 (m, 4H), 7.14 (d, *J* = 2.62 Hz, 1H), 6.96 (d, *J* = 9.01 Hz, 1H), 6.74 (dd, *J*_1_ = 9.06 Hz, *J*_2_ = 2.56 Hz, 1H), 4.25 (s, 2H), 3.79 (s, 3H), 2.82 (s, 4H), 2.27 (s, 3H). ^13^C NMR (100 MHz, DMSO-d_6_) δ (ppm): 170.66, 168.34, 167.37, 156.12, 138.28, 136.52, 134.38, 131.73, 130.62, 130.42, 129.56, 115.10, 112.39, 111.27, 101.81, 55.82, 25.94, 13.64. HRMS-ESI: calculated [M+Na]^+^ *m*/*z* = 477.0829, found = 477.0816.

### 2.4. Synthesis of 2-acetoxybenzoic Anhydride (Aspirin Anhydride)

Aspirin anhydride was synthesised following a previous method [32]. Aspirin (0.5 g, 2.8 mmol) was dissolved in DCM (7 mL), and a solution of DCC (0.286 g, 1.4 mmol) in DCM (3 mL) was added. The solution was left to stir overnight at room temperature. The dicyclohexylurea (DCU) byproduct was filtered off and the solid was washed with DCM. The filtrate was evaporated to dryness and re-dissolved in ethyl acetate before being filtered to remove residual DCU. The filtrate was evaporated, producing a transparent oil which was either used immediately in the next synthetic step or stored at −20 °C. Yield (695.4 mg, 73%). ^1^H NMR (400 MHz, DMSO-d_6_) δ (ppm): 7.93 (d, *J* = 7.86 Hz, 2H), 7.64 (t, *J* = 7.76 Hz, 2H), 7.38 (t, *J* = 7.58 Hz, 2H), 7.20 (d, *J* = 8.02 Hz, 2H), 2.25 (s, 6H).

### 2.5. Synthesis of [Pt(P_L_)(SSDACH)(Indomethacin)(OH)](NO_3_)_2_ (where P_L_= Phen (**1**) or 56Me_2_Phen (**2**))

**Scheme 1:**
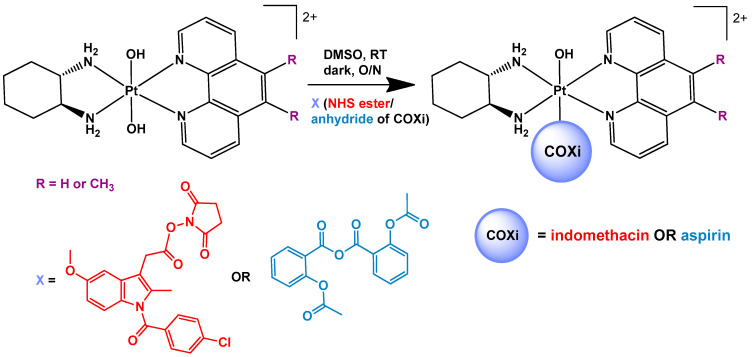
The synthetic scheme for the synthesis of the platinum(IV) prodrugs comprising of COX inhibitors (COXi) indomethacin (**1** and **2**) and aspirin (**3** and **4**) in the axial positions.

The conjugation of indomethacin-NHS to either **PHENSS(IV)** or **56MESS(IV)** followed the route in Scheme 1. [Pt(P_L_)(SSDACH)(OH)_2_](NO_3_)_2_ (200 mg) and 1.2 molar equivalents of indomethacin-NHS ester were dissolved in DMSO (2 mL) and stirred at room temperature for 5–10 h in the dark. The resulting bright yellow solution was diluted with MeOH (5 mL) and precipitated with ether (~60 mL). The yellow precipitate was collected by vacuum filtration and washed with ether. To purify **1** and **2**, flash chromatography was used, with complexes eluted through a 12 g C18 column at a flowrate of 4 mL/min over various H_2_O:MeOH gradients (detailed in Supplementary Materials Table S1).

#### 2.5.1. [Pt(Phen)(SSDACH)(Indomethacin)(OH)](NO_3_)_2_ (**1**)

Yield (198.2 mg, 65%). Electronic spectrum λ_max_ nm (ε/M^−1^ cm^−1^, water): 201 (161,400 ± 430), 276 (50,000 ± 115), 303 (16,650 ± 120). CD spectrum λ_max_ nm (mdeg mol L^−1^, water): 206 (−2.8), 267 (0.3), 290 (−0.4). ^1^H NMR (400 MHz, D_2_O) δ (ppm): 9.20 (d, *J* = 6.56 Hz, 1H), 9.15 (d, *J* = 6.00 Hz, 1H), 8.82 (m, 2H), 8.14 (dt, *J*_1_ = 8.16 Hz, *J*_2_ = 5.23 Hz, 2H), 7.99 (s, 2H), 7.53 (m, 4H), 6.47 (dd, *J*_1_ = 9.04 Hz, *J*_2_ = 2.59 Hz, 1H), 6.32 (d, *J* = 9.00 Hz, 1H), 6.18 (d, *J* = 2.52 Hz, 1H), 3.68 (s, 3H), 3.26 (m, 4H), 2.42 (m, 2H), 1.70 (m, 4H), 1.60 (s, 3H), 1.32 (m, 2H). ^1^H-^195^Pt HMQC (400/86 MHz, D_2_O): δ 9.22, 9.14/557. ^195^Pt NMR (86 MHz, D_2_O): δ 557 ppm. HPLC *t*_R_: 8.8 min. HRMS-ESI: calculated [M]^+^ *m*/*z* = 862.2209, found = 862.2231.

#### 2.5.2. [Pt(56Me_2_Phen)(SSDACH)(Indomethacin)(OH)](NO_3_)_2_ (**2**)

Yield (189.4 mg, 63%). Electronic spectrum λ_max_ nm (ε/M^−1^ cm^−1^, water): 202 (193,500 ± 215), 282 (51,600 ± 145), 313 (19,900 ± 245). CD spectrum λ_max_ nm (mdeg mol L^−1^, water): 210 (−2.9), 242 (−1.2), 274 (0.7), 295 (−0.9). ^1^H NMR (400 MHz, D_2_O) δ (ppm): 9.13 (d, *J* = 5.40 Hz, 1H), 9.08 (d, *J* = 5.40 Hz, 1H), 8.97 (m, 2H), 8.13 (dt, *J*_1_ = 8.67 Hz, *J*_2_ = 5.53 Hz, 2H), 7.48 (m, 4H), 6.47 (m, 2H), 6.14 (s, 1H), 3.64 (s, 3H), 3.23 (m, 4H), 2.68 (s, 6H), 2.42 (m, 2H), 1.70 (m, 4H), 1.60 (s, 3H), 1.31 (m, 2H). ^1^H-^195^Pt HMQC (400/86 MHz, D_2_O): δ 9.15, 9.07/540. ^195^Pt NMR (86 MHz, D_2_O): δ 540 ppm. HPLC *t*_R_: 9.2 min. HRMS-ESI: calculated [M]^+^ *m*/*z* = 890.2522, found = 890.2557.

### 2.6. Synthesis of [Pt(P_L_)(SSDACH)(Aspirin)(OH)](NO_3_)_2_ (P_L_= Phen (**3**) or 56Me_2_Phen (**4**))

The conjugation of aspirin-anhydride to either **PHENSS(IV)** or **56MESS(IV)** followed the route in Scheme 1. [Pt(P_L_)(SSDACH)(OH)_2_](NO_3_)_2_ (200 mg) and 1.3 molar equivalents of aspirin anhydride were dissolved in DMSO (2.5 mL) and stirred at room temperature overnight in the dark. The resulting yellow solution was diluted with MeOH (7 mL) and precipitated with ether (~40 mL), producing a sticky brown residue. This was centrifuged and the supernatant was discarded. The brown residue was dissolved in MeOH (2 mL) and ether (30 mL) was added, resulting in a white precipitate. The mixture was centrifuged and the supernatant discarded. The solid was washed with ether two more times to afford a white powder, which was collected. To purify **3** and **4**, flash chromatography was used, with complexes eluted through a 12 g C18 column at a flowrate of 4 mL/min over various H_2_O:MeOH gradients (detailed in Table S1).

#### 2.6.1. [Pt(Phen)(SSDACH)(Aspirin)(OH)](NO_3_)_2_ (**3**)

Yield (172.6 mg, 69%). Electronic spectrum λ_max_ nm (ε/M^−1^ cm^−1^, water): 202 (94,900 ± 295), 279 (28,900 ± 75), 306 (8050 ± 40). CD spectrum λ_max_ nm (mdeg mol L^−1^, water): 215 (−2.3). ^1^H NMR (400 MHz, D_2_O) δ (ppm): 9.32 (d, *J* = 5.56 Hz, 1H), 9.28 (d, *J* = 5.60 Hz, 1H), 9.11 (dd, *J*_1_ = 8.41 Hz, *J*_2_ = 4.08 Hz, 1H), 8.35 (s, 2H), 8.29 (m, 2H), 7.59 (d, *J* = 7.83 Hz, 1H), 7.42 (t, *J* = 7.80 Hz, 1H), 7.18 (t, *J* = 7.68 Hz, 1H), 6.88 (d, *J* = 8.08 Hz, 1H), 3.24 (m, 2H), 2.42 (m, 2H), 1.72 (m, 4H), 1.60 (s, 3H), 1.33 (m, 2H). ^1^H-^195^Pt HMQC (400/86 MHz, D_2_O): δ 9.30, 8.28/536. ^195^Pt NMR (86 MHz, D_2_O): δ 536 ppm. HPLC *t*_R_: 6.1 min. HRMS-ESI: calculated [M-H]^+^ *m*/*z* = 684.1786, found = 684.1786.

#### 2.6.2. [Pt(56Me_2_Phen)(SSDACH)(Aspirin)(OH)](NO_3_)_2_ (**4**)

Yield (166.2 mg, 67%). Electronic spectrum λ_max_ nm (ε/M^−1^ cm^−1^, water): 200 (108,200 ± 330), 291 (32,200 ± 105), 317 (7850 ± 25). CD spectrum λ_max_ nm (mdeg mol L^−1^, water): 216 (−2.1). ^1^H NMR (400 MHz, D_2_O) δ (ppm): 9.21 (m, 4H), 8.25 (m, 2H), 7.57 (d, *J* = 7.84 Hz, 1H), 7.41 (t, *J* = 7.81 Hz, 1H), 7.17 (t, *J* = 7.64 Hz, 1H), 6.86 (d, *J* = 8.04 Hz, 1H), 3.22 (m, 2H), 2.84 (s, 6H), 2.41 (m, 2H), 1.71 (m, 4H), 1.56 (s, 3H), 1.32 (m, 2H). ^1^H-^195^Pt HMQC (400/86 MHz, D_2_O): δ 9.26, 8.26/525. ^195^Pt NMR (86 MHz, D_2_O): δ 525 ppm. HPLC *t*_R_: 6.6 min. HRMS-ESI: calculated [M-H]^+^ *m*/*z* = 712.2099, found = 712.2098.

### 2.7. NMR Spectroscopy

NMR experiments were carried out using a Bruker (Melbourne, Australia) Avance 400 MHz spectrometer to obtain 1D and 2D spectra. To acquire ^1^H NMR spectra, a spectral width of 8251 Hz with 65,536 data points was used. The ^13^C NMR spectra were recorded using a spectral width of 24,038 Hz and 65,536 data points. For the COSY spectra, a spectral width of 8013 Hz for both ^1^H nuclei (F1 and F2 dimensions) was utilised, with 256 data points for F1 and 2048 data points for F2. To obtain ^195^Pt NMR spectra, a spectral width of 85,470 Hz and 674 data points was employed. The ^1^H-^195^Pt HMQC spectra were collected over a spectral width of 215,156 Hz with 256 data points for the ^195^Pt nucleus (F1 dimension) and a spectral width of 4808 Hz with 2048 data points for the ^1^H nucleus (F2 dimension). Chemical shifts are reported in parts per million (ppm) and the *J* coupling constants are reported in Hz. Samples were prepared in either D_2_O or DMSO-d_6_; data were acquired at 298 K unless otherwise specified

### 2.8. Flash Chromatography

A Biotage Isolera^TM^ One flash chromatography system (Shimadzu, Sydney, Australia) was employed to purify complexes **1**–**4**. This was equipped with a Biotage SNAP KP-C18-HS 12 g cartridge (Shimadzu, Sydney, Australia). Samples were dissolved in minimal water and eluted over different gradients of H_2_O:MeOH (Table S1) at a flow rate of 4 mL/min. A photodiode array (PDA) was used to detect absorbance at 230 and 280 nm.

### 2.9. Ultraviolet (UV) Absorption Spectroscopy

Absorbance spectra were obtained using an Agilent (Melbourne, Australia) Cary 3500 UV-Vis spectrophotometer. Complexes **1**–**4**, indomethacin, aspirin, Phen and 56Me_2_Phen were prepared in either water or MeOH at room temperature using a 1 cm quartz cuvette, and absorption data was recorded from 190–400 nm with either water or MeOH as the reference. For the titration of **1**–**4**, a stock solution of each compound (1 mM) was prepared and small aliquots (ranging from 3–6 µL) were titrated into a cuvette containing water (3000 µL) and the spectra recorded. Experiments were repeated in triplicate and the average extinction coefficients were determined.

### 2.10. Circular Dichroism (CD) Spectroscopy

CD spectra for **1**–**4** were recorded using a Jasco (Easton, United States) J-810 CD spectropolarimeter in the range 200–400 nm, with nitrogen gas flowing at 6 L/min. Samples were prepared in water and spectra were obtained using a 1 cm cylindrical quartz cuvette, with 50 accumulations per sample obtained at room temperature. A scan speed of 100 nm/min, a response of 1 s, a spectral bandwidth of 1 nm, a sensitivity setting of 100 mdeg, and a data pitch of 1 nm were used for each sample. A water baseline was acquired and subtracted from each spectrum. The HT level remained below 450 V for all experiments.

### 2.11. Synchrotron Radiation Circular Dichroism (SRCD) Spectroscopy

SRCD spectra for **1** and **2** were obtained using the AU-CD beamline on ASTRID2 at ISA, Centre for Storage Ring Facilities, Aarhus University, Aarhus, Denmark. This beamline operates in top-up mode over a wavelength range of 125–450 nm. It uses a bandwidth of 0.6 nm and a current of 120 mA. A sample to detector distance of 25 mm was used, with a beam size of 2 × 6 mm (vertical × horizontal) on the sample. D-10-camphorsulfonic acid was used for calibration of the spectropolarimeter. Data were collected in the 176–350 nm range with 1 nm increments over six accumulations at 25 °C. A suprasil quartz cuvette with a path length of 0.01 cm was used. Both samples were prepared in H_2_O, with the water baseline obtained and subtracted from each spectrum.

### 2.12. Mass Spectrometry (MS)

ESI-MS data were acquired using a Waters (Sydney, Australia) SYNAPT G2-S quadruple time-of-flight (QTOF) HDMS equipped with an ESI source and in positive ion mode. The capillary voltage ranged from 1–2.0 kV and spectra were obtained in the range *m*/*z* = 50–1200 Da. Samples were prepared in H_2_O at a concentration of 10–100 ng/mL.

### 2.13. High Performance Liquid Chromatography (HPLC) Conditions and Lipophilicity Measurements

HPLC experiments were executed on an Agilent (Melbourne, Australia) Technologies 1260 Infinity instrument equipped with a Phenomenex (Sydney, Australia) Onyx^TM^ Monolithic C18 reverse phase column (100 × 4.6 mm, 130 Å). The mobile phase was comprised of 0.06% TFA in water (solvent A) and 0.06% TFA in an acetonitrile-water mixture (9:1) (solvent B). A sample injection volume of 10 µL was used at a flow rate of 1 mL/min and eluted over a gradient of 0–100% for 15 min. A PDA was used to detect the eluting peaks at 254 nm. Lipophilicity experiments to determine $\log k_{w}$ values were undertaken following previously published methods [25]. Rather than a gradient, different isocratic ratios ranging from 28–56% of solvent B were employed and the retention times measured for each complex. Potassium iodide was used as an external dead volume marker to determine the dead time of the column. The following equation was used to calculate the capacity factors:

(1) $$k=\frac{t_{r}-t_{0}}{t_{0}}$$

where *k* is the capacity factor, *t_r_* is the retention time of the analyte, and *t*_0_ is the dead time. Following this, log *k* was plotted against the percentage of ACN. Five different concentrations of ACN were used to produce a linear plot comprised of R^2^ > 0.99. This graph produced the following equation:

(2) $$\log k=S\varphi +\log k_{w}$$

where *S* is the slope, φ is the % ACN, and the *y*-intercept, represented as $\log k_{w}$, is the capacity factor of the compound in 100% water.

### 2.14. In Vitro Cytotoxicity Assay

The in vitro cytotoxicity assays were completed at Calvary Mater Hospital, Waratah, NSW, Australia, following a previously published method [40]. A stock solution for each sample was prepared in DMSO (30 mM) and stored at −20 °C. The cell lines used in the study included HT29 colon, U87 glioblastoma, MCF-7 breast, A2780 ovarian, H460 lung, A431 skin, Du145 prostate, BE2-C neuroblastoma, SJ-G2 glioblastoma, MIA pancreas and cisplatin-resistant ADDP ovarian (subclone of A2780 cell line) as well as the non-tumour derived MCF10A breast cell line. The cancer cell lines were cultured in a humidified atmosphere of 5% CO_2_ at 37 °C and maintained in Dulbecco’s modified Eagle’s medium (Trace Biosciences, Sydney, Australia) supplemented with 10% foetal bovine serum, sodium bicarbonate (10 mM), penicillin (100 IU/mL), streptomycin (100 µg/mL) and glutamine (4 mM). The non-cancerous MCF10A cell line was cultured in DMEM:F12 (1:1) cell culture media with 5% heat inactivated horse serum supplemented with penicillin (50 IU/mL), streptomycin (50 µg/mL), glutamine (2 mM), HEPES (20 mM), epidermal growth factor (20 ng/mL), hydrocortisone (500 ng/mL), cholera toxin (100 ng/mL) and insulin (10 µg/mL). Using 96-well plates, cells were plated in duplicate in medium (100 µL) at a density of 2500–4000 cells per well. On day 0, (24 h after plating) when the cells were in logarithmic growth, medium (100 µL) with or without the test agent was added to each well. After 72 h drug exposure the growth inhibitory effects were evaluated using MTT (3-(4,5-dimethylthiazol-2-yl)-2,5-diphenyltetrazolium bromide) assay, with the absorbance determined at 540 nm. An eight-point dose-response curve was produced, which was used to determine the concentration at which the growth was inhibited by 50% (GI_50_). This calculation was based on the difference between the optical density values on day 0 and those at the end of drug exposure. The GI_50_ values for cisplatin, carboplatin, oxaliplatin and **PHENSS(IV)** were taken from literature, where the same method was used [18,25]. GI_50_ values (nM) were reported as the mean and standard error of the mean, and experiments were conducted on three separate occasions. Student’s t-test for unpaired data with equal variance was used to investigate differences in values. All calculations were performed with Statistica v10.0 (StatSoft, Tulsa, OK, USA) using two-tailed tests, and *p*-values < 0.05 were considered statistically significant.

### 2.15. COX Inhibition Assay

An enzyme immunoassay (EIA) kit purchased from Cayman Chemicals (Ann Arbor, United States, catalogue No. 701230) was used to assess the ability of the complexes (**1**–**4**) and the ligands (indomethacin and aspirin) to inhibit human COX-2. The preparation of standards and samples and the completion of the assay were carried out following the manufacturer’s protocol. As described below, the completion of the assay required three main steps: the preparation of standards, the COX reaction and EIA.

The preparation of standards involved serial dilution of the prostaglandin (PG)-screening ELISA standard with ELISA buffer.

The COX reaction involved the following:

- Background samples: COX-2 was inactivated by transferring 20 µL of the enzyme to an Eppendorf tube which was placed in boiling water for 3 min. Solutions containing the reaction buffer (160 µL), heme (10 µL), inactivated COX-2 enzyme (10 µL) and the inhibitor vehicle (DMSO, 10 µL) were prepared in duplicate.
- COX-2 100% initial activity samples: Solutions containing the reaction buffer (160 µL), heme (10 µL), COX-2 enzyme (10 µL) and the inhibitor vehicle (DMSO, 10 µL) were prepared in duplicate.
- COX-2 inhibitor samples: Solutions containing the reaction buffer (160 µL), heme (10 µL), COX-2 enzyme (10 µL) and the sample being investigated (10 µL; indomethacin, complex **1** or **2** at 14 and 1.4 µM, each; aspirin, complex **3** or **4** at 700, 70 and 7 µM each) were added together.

All tubes were incubated for 10 min at 37 °C. To initiate the reaction, arachidonic acid (10 µL) was added to each tube. This was incubated for 30 s at 37 °C before stannous chloride (30 µL) was added to stop enzyme catalysis. The tubes were removed from the water bath and vortexed, then incubated for five minutes at room temperature. The background samples were diluted with ELISA buffer 1:100 times and the COX-2 100% initial activity and COX-2 inhibitor samples were diluted 1:2000 and 1:4000 times.

For EIA, ELISA buffer (100 µL) was added to non-specific binding (NSB) wells and ELISA buffer (50 µL) was added to maximum binding (B_0_) wells; 50 µL from each PG screening standard was added to the standard wells in duplicate. A background sample (50 µL) and COX 100% initial activity samples (50 µL) were added to the wells in duplicate. Each inhibitor sample (50 µL) was added to the wells in triplicate. PG screening acetylcholinesterase (AChE) tracer (50 µL) was added to all wells except for the total activity and blank wells. PG screening ELISA antiserum (50 µL) was added to each well except for the total activity, NSB and blank wells. The plate was covered with plastic film and incubated for 18 h at room temperature on an orbital shaker. The wells were emptied and rinsed with wash buffer five times. Ellman’s reagent (200 µL) was added to each well, followed by AChE tracer (5 µL) in the total activity well. The plate was covered with plastic film and left to develop in the dark using an orbital shaker at room temperature for one hour. The absorbance was recorded at 405 nm using a plate reader.

### 2.16. Cellular Accumulation

Cellular uptake studies were carried out in A2780 (ovarian) and ADDP (cisplatin-resistant ovarian) cancer cell lines. Experiments were repeated in triplicate on three separate occasions. The number of cells per well seeded into 12-well plates for each replicate and cell line are presented in Table S2. After incubating at 37 °C overnight, the cell culture medium was replaced with fresh medium containing the test compounds at concentrations of 1.0 and 0.1 µM, which were incubated for 4 h. Following this, the medium was removed and the cells were washed twice with PBS and incubated with trypsin solution until the cells dissociated from the plate. The harvested cells were then placed into an Eppendorf tube and PBS was added to a volume of 1 mL. Trypan blue staining was then used to determine cell numbers. Cell pellets were obtained after centrifugation, and the cells were dispersed in MilliQ™ water (50 µL) and stored immediately at −20 °C for subsequent platinum analysis.

### 2.17. Platinum Analysis

HNO_3_ 69% (50 µL) was added to each sample which was then digested at 80 °C for 1.5 h before being taken up with H_2_O to 1 mL. A PerkinElmer (Melbourne, Australia) NexION™ 300X inductively coupled plasma mass spectrometer (ICP-MS) was used to carry out the platinum analyses. A certified platinum reference standard was serially diluted to produce a four-point calibration curve. The resulting calibration curve was linear over the working range (*R*^2^ > 0.9999), and the most abundant platinum isotope (*m*/*z* = 195) was monitored. The average values for three independent experiments were calculated and reported with the standard error of the mean.

## 3. Results and Discussion

### 3.1. Synthesis and Characterisation

The synthesis of the platinum(II) precursors **PHENSS(II)** and **56MESS(II),** together with that of the di-hydroxido platinum(IV) precursors **PHENSS(IV)** and **56MESS(IV)** was accomplished following previously published methods with no modifications [25]. To conjugate the COX inhibitors to the di-hydroxido platinum(IV) complexes, the anhydride of aspirin and the NHS ester of indomethacin were initially synthesised. For the synthesis of **3** and **4**, the anhydride of aspirin was used in the reaction to afford the mono-substituted product; however, for the synthesis of **1** and **2**, the NHS ester of indomethacin was required as this was more responsive to acylation than the anhydride. The respective anhydride or ester was then coordinated to either **PHENSS(IV)** or **56MESS(IV)** and stirred overnight in minimal DMSO at room temperature in the dark. For the synthesis of all complexes **1**–**4**, only 1.2–1.3 molar equivalents of the ligands were required for full conversion.

The reactions were monitored using ^195^Pt NMR and HPLC to determine when the mono-substituted complex had formed and to ensure no unreacted di-hydroxido platinum(IV) remained. In DMSO, a platinum chemical shift of ~330 ppm is indicative of di-hydroxido platinum(IV), and when the inhibitor coordinates to the platinum(IV), a new peak at ~480 ppm appears and increases in intensity with increasing concentration, while the peak at ~330 ppm decreases. The signal at ~480 ppm verifies the formation of mono-substituted platinum(IV) complexes from this group in DMSO [25]. When the peak at ~330 ppm was no longer visible, the product was precipitated by initially diluting the crude solution with MeOH, followed by the addition of diethyl ether. Flash chromatography was used to isolate the mono-substituted complexes from any unreacted ligands or di-hydroxido platinum(IV). A C18 12 g cartridge column was employed for all complexes. Each sample was eluted at a flow rate of 4 mL/min using customised gradient elution profiles of H_2_O:MeOH (Table S1). The mono-substituted complexes eluted after any di-hydroxido platinum(IV) and before the unreacted ligand.

Both 1D and 2D NMR spectroscopy were utilised to characterise and assess the structure and purity of each compound (Figures S1–S21). HPLC was used to corroborate the purity of each complex, and showed peak areas greater than 96% for all complexes (Figures S22–S25). ESI-MS produced the correct mass peak and expected isotopic distribution pattern for the platinum complexes, thus confirming their successful synthesis (Figures S26–S30 and Table S3).

### 3.2. NMR Spectroscopy

A combination of 1D (^1^H and ^195^Pt) and 2D (^1^H-^1^H COSY and ^1^H-^195^Pt HMQC) NMR experiments were used to characterise all compounds (Figures S1–S21). The NMR spectra of the initial platinum(II) and platinum(IV) di-hydroxido complexes (**PHENSS(II)**, **56MESS(II)**, **PHENSS(IV)** and **56MESS(IV)**) were all in agreement with the published data [18]. For complexes **1**–**4**, the proton resonances in the aliphatic region arising from the A_L_ SSDACH were in agreement with those published in the literature. Also observed in the aliphatic region is the singlet from the H5/H6 protons of the methyl substituents on the phenanthroline for both **2** and **4** (Figures S10 and S18). The Phen resonances of **1**–**4** were shifted further downfield compared to the resonances of the di-hydroxido platinum(IV) complexes, consistent with asymmetrical complexes from this group in the literature [41]. The Phen protons closest to the platinum centre exhibited signal splitting attributed to the asymmetry of the octahedral complexes, suggesting that the Phen ligand that constitutes the square plane may adopt a slightly distorted conformation.

**Figure 3:**
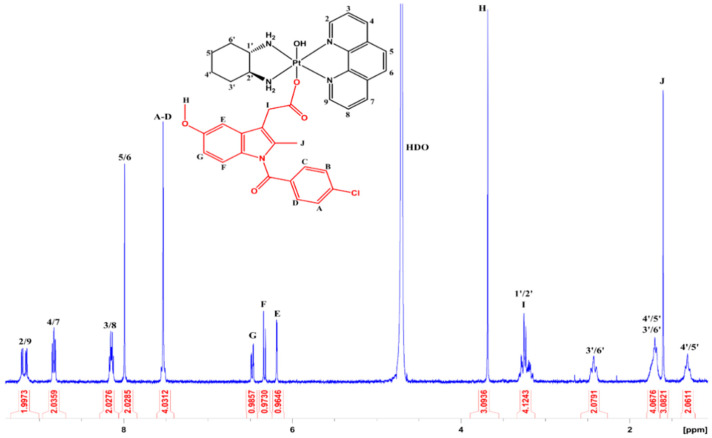
The ^1^H NMR spectrum of [Pt(Phen)(SSDACH)(Indomethacin)(OH)](NO_3_)_2_ (**1**) in D_2_O at 298 K. Insert: The chemical structure of **1**, with assigned numbers/letters.

For the assignment of the protons of the axially bound indomethacin in **1**, signals were observed in both the aliphatic and aromatic region of the ^1^H NMR spectrum (Figure 3). In the aliphatic region, the singlets at 3.68 and 1.60 ppm, integrating for three protons each, were assigned to the methyl groups of indomethacin (protons H and J, respectively), with proton H exhibiting a further downfield shift due to the deshielding from the oxygen. The resonance at 3.23 ppm correlated to proton I, which was merged with the resonances of H1′/H2′ from SSDACH. Protons from the aromatic rings on the indomethacin produced four signals in the aromatic region of the ^1^H NMR spectrum. The multiplet at 7.53 ppm integrated for four protons and was assigned to protons A–D from the aromatic ring, as these were shifted furthest downfield due to the electronegativity of the chloro group. The doublet of doublet at 6.47 ppm, with an integration of one proton, was coupled with the resonances at 6.32 (ortho, *J* = 9.04 Hz) and 6.18 (meta, *J* = 2.59 Hz) ppm in the COSY spectrum (Figure S7), and was thus assigned to proton G. The resonances at 6.32 and 6.18 ppm, each integrating for one proton, were therefore assigned to protons F and E, respectively. The assignment of resonances in the ^1^H NMR spectrum of **2** followed a similar rationale to that described for **1**, with the exception of the proton resonances for G and F merging into a single signal appearing at 6.47 ppm (Figure S10).

For the assignment of the protons of the axially coordinated aspirin in **3**, one signal was observed in the aliphatic region, while there were four peaks in the aromatic region (Figure S14). The singlet at 1.60 ppm integrated for three protons and was therefore assigned to the protons of the methyl group (E). In the aromatic region, the doublets at 7.59 and 6.88 ppm, integrating for one proton each, were assigned to protons A and D, respectively. In the COSY spectrum, the triplet at 7.18 ppm was coupled to A and to the triplet at 7.42 ppm, which displayed coupling to D; accordingly, these were assigned to B and C, respectively. The assignment of resonances in the ^1^H NMR spectrum of **4** followed the same rationale as in **3** (Figure S18). The amine proton resonances were not visible in any of the spectra due to exchange with D_2_O.

To confirm conjugation of the axial ligands to the platinum centre, the ^195^Pt and ^1^H-^195^Pt HMQC NMR spectra were obtained. Due to the sensitivity of the platinum coordination sphere, a significant difference in the chemical shift was observed when this was altered [42]. Platinum(II) and di-hydroxido platinum(IV) complexes from this class of compounds have chemical shifts of ~−2800 and ~400 ppm (in D_2_O), respectively [18,19]. Complexes **1**–**4** are all mono-substituted, and thus experienced a downfield chemical shift occurring at ~530 ppm (in D_2_O). Indeed, this remains consistent with other mono-substituted compounds from this class of platinum(IV) complexes, providing confirmation of the successful conjugation of the axial ligand to the platinum centre [25,41].

### 3.3. Electronic Spectra

**Figure 4:**
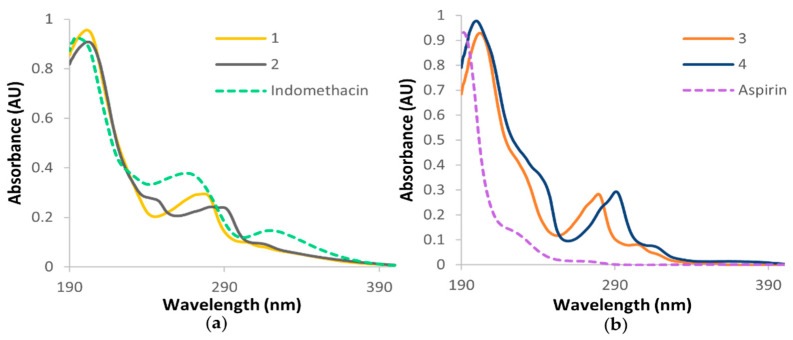
The UV spectra of (**a**) **1**, **2** and indomethacin and (**b**) **3**, **4** and aspirin, in water.

The UV spectra were recorded for prodrugs **1**–**4**, the axial ligands and the P_L_, and molar extinction coefficients were determined for **1**–**4** (Figures S31–S35 and Table S3). The absorbance bands were in agreement with similar compounds in the literature, with the expected π–π* transitions arising from the P_L_ and the metal-to-ligand charge-transfer (MLCT) interactions [18]. Prodrugs **1**–**4** each displayed two strong absorbance bands (~200 and ~280 nm) and one weaker band (~310 nm), as depicted in Figure 4. For **1** and **3**, with the P_L_ Phen, the most notable absorbance bands were observed at 201 and 202 nm, respectively, with the second bands at 276 and 279 nm, respectively, arising from the Phen π–π* transitions. In **2** and **4**, consisting of the P_L_ 56Me_2_Phen, two similar strong absorbance bands were exhibited; however, they were red-shifted compared to the Phen derivatives. This red-shift was more prominent in the 280 nm region, where **2** exhibited a red-shift of 6 nm compared to **1**, and **4** exhibited a red-shift of 12 nm compared to **3**. This red-shift is attributed to the methyl groups on the P_L_ and was confirmed by the UV spectra of the individual P_L_, which shows the peak absorbances of 56Me_2_Phen to be red-shifted compared to those exhibited by Phen (Figure S35). This trend is consistent with previously published compounds with the same P_L_ [18,25]. The third absorbance band depicted in the spectra of all prodrugs, at ~310 nm, is owing to MLCT interactions, which shows the 56Me_2_Phen prodrugs (**2** and **4**) to be red-shifted (by ~10 nm) compared to the Phen prodrugs (**1** and **3**). Additionally, the aspirin prodrugs (**3** and **4**) were slightly red-shifted (by ~3 nm) compared to the indomethacin prodrugs (**1** and **2**). This may be influenced by the strong absorbance exhibited by indomethacin at ~320 nm (Figure 4a). Prodrugs **2**–**4** exhibited shoulders at ~240 nm, which is a trend displayed by their platinum(IV) di-hydroxido precursors [18]. This shoulder was not observed in **1**, and may have merged with the absorbance band at ~280 nm.

CD and SRCD spectroscopy were used to confirm the chirality of the complexes (Figures S36–S41). As the chirality of SSDACH is a major contributing factor to the activity of these compounds, it is essential that the chirality of this ligand was retained during synthesis. The CD spectra of the indomethacin prodrugs (**1** and **2**) exhibited negative peaks at ~208 nm and positive peaks at ~270 nm, with **2** showing two additional negative peaks at 242 and 295 nm (Figures S36 and S37). There is evidence of a signal at ~290 nm for **1**; however, it could not be clearly defined within the operating range of the instrument. SRCD spectra of **1** and **2** were obtained to gain data below 200 nm, and while no additional peaks were observed, the peak at 290 nm for **1** was much more prominent and thus able to be clearly identified (Figures S38 and S39). The peaks in the CD spectra for the aspirin prodrugs (**3** and **4**) were much less defined than the spectra of **1** and **2**, and only the negative peaks at ~215 nm were clearly identified (Figures S40 and S41). This observed pattern is consistent with previous complexes of this type where SSDACH is coordinated with platinum, thus confirming that chirality was conserved during synthesis [18].

### 3.4. Preliminary Reduction Studies

**Figure 5:**
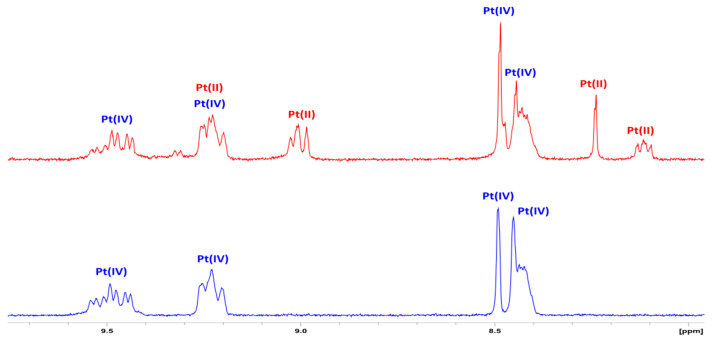
The ^1^H NMR spectra of the aromatic region of **3** with 10X PBS in D_2_O, at 37 °C (**bottom**) and with ascorbic acid added (**top**), showing platinum(II) resonances.

Given that **1**–**4** were designed on the premise of functionating as prodrugs, it is anticipated that these complexes be reduced to platinum(II) and release their respective axial ligands, which will allow each component to exert its activity. To explore this possibility, preliminary NMR reduction studies were undertaken for **3**. The sample was prepared in D_2_O with 10X PBS before ascorbic acid was added, and the sample was monitored by ^1^H NMR at 37 °C. After the addition of ascorbic acid, resonances corresponding to the platinum(II) species appeared, with the intensities increasing over time, while the peaks correlating to platinum(IV) species decreased (Figure 5). Following this, ^195^Pt NMR was carried out in order to confirm that the platinum(II) species remained intact. A peak was observed at −2808 ppm, while the peak at 536 ppm disappeared, confirming reduction to its platinum(II) congener (Figure S42) [18]. While these conditions do not entirely mimic the biological environment to which these complexes would be exposed, this study indicates that these complexes reduce to their platinum(II) congeners when exposed to reducing agents.

### 3.5. Lipophilicity Studies

Lipophilicity studies were carried out to determine the $\log k_{w}$ of each complex. This was ascertained through a RP-HPLC based approach whereby complexes were eluted at several isocratic ratios. In turn, retention times were employed as in Equation (1), where the capacity factor (*k*) was calculated. The log *k* value at each ratio was then plotted against the percentage of the organic solvent used in solvent B, producing a linear equation that allowed for $\log k_{w}$ to be extrapolated (Figure S43). The $\log k_{w}$ value represents the retention factor in a 100% aqueous solution; thus, higher values indicate more lipophilic compounds. While this method for determining lipophilicity is slightly different from the shake-flask method, it has been used previously to compare a series of complexes that are structurally similar and is fitting for this group of complexes [41].

The order of complexes by increasing lipophilicity is **3** < **4** < **1** < **2**. According to the $\log k_{w}$ values presented in Table 1, it is evident that the indomethacin complexes (**1** and **2**) are more lipophilic than the aspirin complexes (**3** and **4**). Indomethacin and aspirin have log P values of 4.27 and 1.18, respectively, and thus correspond with the lipophilicity of the complexes [43,44]. Additionally, the 56MESS derivatives are more lipophilic than their corresponding PHENSS derivatives, which could be attributed to the methyl groups on the P_L_. These results indicate that the axial ligands have more of an effect on the overall lipophilicity of the compounds than does altering the groups on the P_L_ (Table 1).

### 3.6. In Vitro Cytotoxicity

The in vitro cytotoxicity of **1**–**4**, their platinum(II) and platinum(IV) precursors (**PHENSS(II)**, **56MESS(II)**, **PHENSS(IV)** and **56MESS(IV)**) and the COX inhibitors (indomethacin and aspirin) against a broad range of cancer cell lines (HT29 colon, U87 glioblastoma, MCF-7 breast, A2780 ovarian, H460 lung, A431 skin, Du145 prostate, BE2-C neuroblastoma, SJ-G2 glioblastoma, MIA pancreas and cisplatin-resistant ADDP ovarian) as well as a non-cancerous (MCF10A breast) cell line was analysed using MTT (3-(4,5-dimethylthiazol-2-yl)-2,5-diphenyltetrazolium bromide) assays (Table S4). This panel of cell lines and the 72 h incubation time were chosen to allow for comparison to similar complexes previously published [18,25]. As platinum-based compounds are most commonly used to treat ovarian cancer, we selected the A2780 (ovarian) and ADDP (cisplatin-resistant ovarian) cancer cell lines in order to explore the ability of these complexes to overcome cisplatin resistance.

**Figure 6:**
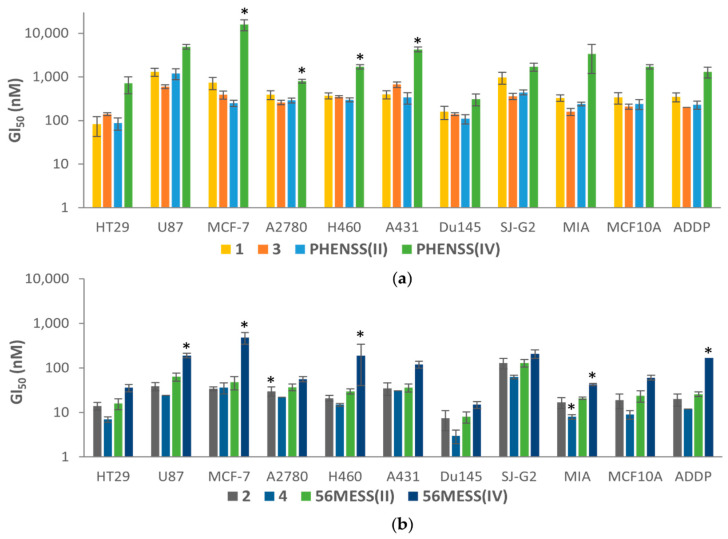
The GI_50_ values of (**a**) **1**, **3**, **PHENSS(II)** and **PHENSS(IV)**, and (**b**) **2**, **4**, **56MESS(II)** and **56MESS(IV),** across several cancer cell lines after 72 h drug exposure. GI_50_ values (nM) are the mean and reported with standard error of the mean; results are produced from three independent experiments. Significant differences between the compounds and **PHENSS(II)** or **56MESS(II)** are shown as *, at *p* < 0.05.

Compounds **1**–**4** displayed exceptional activity across the entire panel of cell lines, with GI_50_ values ranging from as low as 3 nM up to 1.3 µM (Table S4). The complexes consisting of the 56MESS core (**2** and **4**, Figure 6b) were on average twelve-fold more active than the PHENSS derivatives (**1** and **3**, Figure 6a) across all cancer cell lines, which is consistent with other published results including these core complexes [25,41]. Complexes **1**–**4** displayed enhanced activity compared to their corresponding di-hydroxido platinum(IV) derivatives in most cell lines, more so for the PHENSS derivatives; where **1** and **3** were on average seven-fold and ten-fold more active than **PHENSS(IV)**, and **2** and **4** were on average four-fold and 5.5-fold more active than **56MESS(IV)** across all the cancer cell lines, as depicted in Figure 6.

The GI_50_ values of **1** and **3** were comparable to their platinum(II) precursor, **PHENSS(II)** (Figure 6a), suggesting that the conjugation of the COX inhibitor neither hinders nor improves cytotoxicity. The observed activity could be a result of the complex reducing within the cellular matrix, enabling **PHENSS(II)** to exert its cytotoxic activity. In contrast, the 56MESS derivatives, **2** and **4**, both displayed improved activity compared to their precursor, **56MESS(II)**, more so with **4**, the aspirin derivative, which exhibited up to three-fold enhanced activity (U87 and Du145) compared with **56MESS(II)**, suggesting that the axial ligand contributes to the activity (Figure 6b). The aspirin prodrugs (**3** and **4**) both exhibited, on average, ~1.5-fold greater activity than the indomethacin prodrugs (**1** and **2**) across all the cancer cell lines tested. This is interesting, as the indomethacin prodrugs are more lipophilic than the aspirin prodrugs (Table 1), suggesting that the enhanced activity exhibited by **3** and **4** is not solely influenced by lipophilicity.

**Figure 7:**
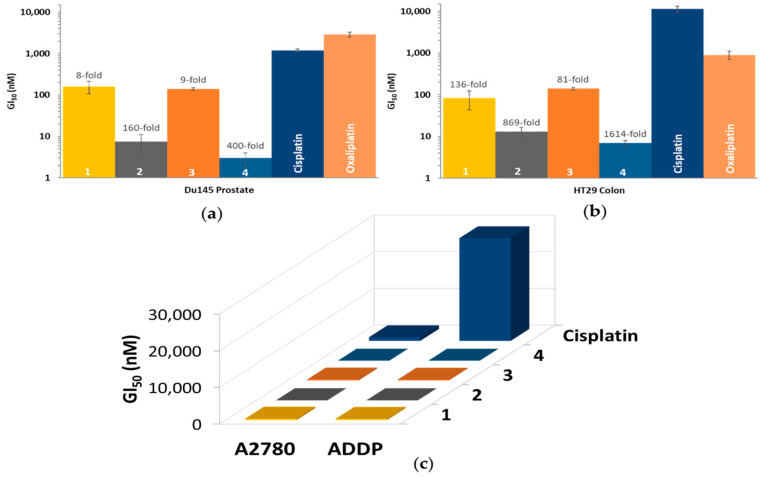
(**a**) and (**b**): The GI_50_ values of **1**–**4**, cisplatin and oxaliplatin in the Du145 prostate (**a**) and HT29 colon (**b**) cancer cell lines, with the (number)-fold potency of **1**–**4** compared to cisplatin; (**c**) The GI_50_ values of **1**–**4** and cisplatin in the A2780 (ovarian) and ADDP (cisplatin-resistant ovarian) cancer cell lines. Complexes were incubated for 72 h; GI_50_ values (nM) are the mean and reported with standard error of the mean; results are produced from three independent experiments.

Moreover, prodrugs **1**–**4** displayed notable specificity towards the Du145 prostate and HT29 colon cancer cells. This trend was observed for the platinum(II) and di-hydroxido platinum(IV) precursors as well, implying that the selectivity for these cell lines may be attributed to the platinum core rather than the axial ligands. Prodrug **4** was the most active complex, exhibiting the lowest GI_50_ value of 3 nM in the Du145 prostate cancer cell line (Figure 7a). Cisplatin exhibited a 28-fold decrease in activity in the cisplatin-resistant ADDP cell line as compared to the A2780 cell line (Figure 7c). Prodrugs **1**–**4**, on the other hand, only exhibited a 1.1–1.8-fold decrease in the cisplatin-resistant cell line, indicating that these prodrugs can overcome cisplatin resistance. Overall, compared to cisplatin, oxaliplatin and carboplatin, most complexes exhibited significantly enhanced activity, with **4** showing 1614-fold increase in activity relative to cisplatin against the HT29 colon cancer cell line (Figure 7b). While **1**–**4** exhibited remarkable activity across the entire panel of cancer cell lines, they exhibited activity in the non-cancerous MCF10A breast cell line as well.

### 3.7. COX Inhibition

**Figure 8:**
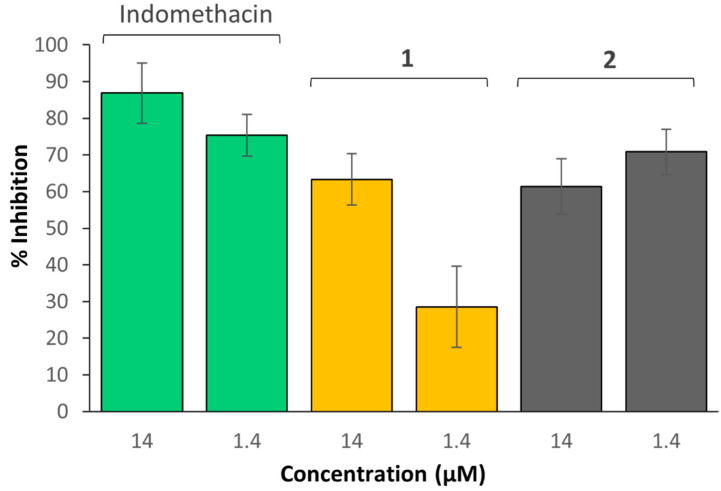
Inhibition of human COX-2 by **1**, **2** and indomethacin, at 14 and 1.4 µM.

To explore the in vitro COX inhibitory properties of these complexes, they were assessed using an enzyme immunoassay (EIA) against the COX-2 enzyme. The COX-2 enzyme was chosen as it is upregulated in certain tumours and contributes to tumour progression [35]. Several different concentrations of the indomethacin and aspirin compounds were assessed; however, higher concentrations were selected for aspirin because it is a less potent inhibitor of COX-2 compared to indomethacin [35]. Aspirin and the aspirin prodrugs (**3** and **4**) were prepared at concentrations ranging from (7–700 µM), with aspirin only showing minimal (19%) COX-2 inhibition at 700 µM. No inhibition was exhibited by **3**, **4**, or aspirin at lower concentrations (7 and 70 µM, as depicted in Figure S44), suggesting the need for higher concentrations. Contrary to this, indomethacin and the indomethacin prodrugs (**1** and **2**) exhibited COX-2 inhibition, despite being prepared at lower concentrations of 14 and 1.4 µM. As depicted in Figure 8, at 14 µM, **1** and **2** showed 63% and 61% inhibition, respectively, and at 1.4 µM, **1** and **2** showed 29% and 71% inhibition, respectively. Indomethacin on its own exhibited 87% and 75% inhibition at 14 and 1.4 µM, respectively. While the COX-2 inhibition shown by **1** and **2** was not as effective as indomethacin, the results suggest that the complexes may bind to the COX-2 enzymes, or alternatively reduce and then release the ligands, which then bind to the enzymes.

No correlation between COX inhibition and the in vitro cytotoxicity of these complexes was evident. While the aspirin complexes exhibited enhanced cytotoxicity, the indomethacin complexes displayed enhanced COX inhibition. It is possible that the in vitro cytotoxicity of **1**–**4** is not necessarily a result of COX inhibition and could be due to other factors. Nevertheless, the exhibited COX-2 inhibitory properties of **1** and **2** indicate they may be able to reduce tumour-associated inflammation and could be selective towards tumours that overexpress this enzyme.

### 3.8. Platinum Uptake Studies

**Figure 9:**
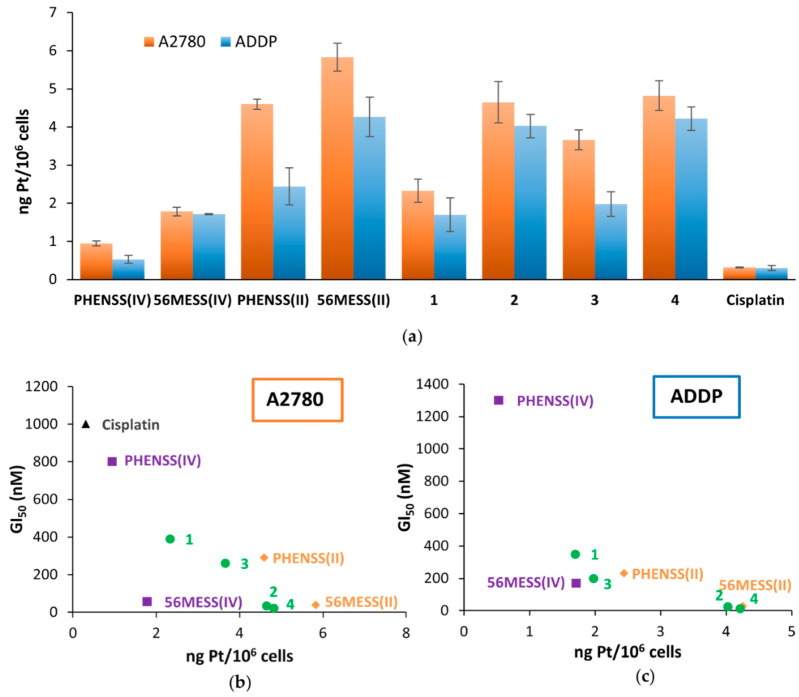
(**a**) Cellular accumulation levels of **1**–**4**, their platinum(II) and platinum(IV) precursors and cisplatin against A2780 (ovarian) and ADDP (cisplatin-resistant ovarian) cancer cells that were treated with 1.0 µM for 4 h. Values are reported in ng Pt/10^6^ cells, are reported with standard error of the mean, and were produced from three independent experiments. (**b**) and (**c**): The correlation between the cellular accumulation and cytotoxicity of **1**–**4**, their platinum(II) and platinum(IV) precursors and cisplatin against A2780 (**b**) and ADDP (**c**) cancer cells treated with 1.0 µM for 4 h. Note: in order to avoid skewing the graph in (**c**), data for cisplatin have been omitted as its GI_50_ value against the ADDP cell line was 28,000 nM.

Cellular accumulation studies of **1**–**4** and their platinum(II) and platinum(IV) precursors, as well as cisplatin, were carried out using the A2780 (ovarian) and ADDP (cisplatin-resistant ovarian) cancer cell lines. ICP-MS was used to determine cellular platinum levels after the cells were exposed to concentrations of 1.0 and 0.1 µM of each compound for 4 h (Figure 9a and Figure S45). These conditions were chosen as a compromise between the high toxicity of these platinum complexes and the quantification range of platinum by ICP-MS. As depicted in Figure 9a, the platinum uptake levels of **3** were 1.2–1.6-fold greater than **1**, and the platinum uptake levels of **4** were 1–1.3-fold greater than **2**. These results show that the aspirin complexes (**3** and **4**) exhibited greater uptake than their analogous indomethacin counterparts (**1** and **2**, respectively). The order of increasing platinum uptake is **1** < **3** < **2** < **4**, which differs from the order of increasing lipophilicity (**3** < **4** < **1** < **2**), indicating that lipophilicity is not the predominant factor in their uptake. It is possible that the uptake of these complexes may be influenced more significantly by active transport mechanisms.

There is a linear correlation between the cellular uptake and cytotoxicity of the complexes (Figure 9b,c), showing that as the platinum uptake level increases, the cytotoxicity does as well (i.e., the GI_50_ values decrease). The least cytotoxic compound, cisplatin, exhibited the lowest uptake. This was followed by the Phen derivatives (**PHENSS(IV)**, **PHENSS(II)**, **1** and **3**), while the 56MESS derivatives (**56MESS(IV)**, **56MESS(II)**, **2** and **4**), were the most cytotoxic and exhibited higher levels of intracellular uptake. While the uptake level of cisplatin in the ADDP cell line was the lowest, it was only ~two-fold lower than **PHENSS(IV)**, which exhibited the next highest uptake. When comparing the GI_50_ values of these two compounds, cisplatin was ~22-fold less active than **PHENSS(IV)**. Cisplatin was therefore omitted from this correlation in the ADDP cell line (Figure 9c), as it is resistant to ADDP and due to its high GI_50_ value it skews the graph. This indicates that cisplatin’s resistance in this cell line is not a result of its uptake. For the rest of the complexes in the ADDP cell line and for all complexes in the A2780 cell line, the results indicate that their higher cellular uptake may be contributing to the enhanced cytotoxicity exhibited by these complexes; however, it is not clear whether their lipophilicity influences their activity. This is inconsistent with data reported previously for similar complexes, which displayed no clear correlation between lipophilicity, cytotoxicity and platinum uptake. This could be due to the nature of the axial ligands (indomethacin and aspirin) as compared to the long-chain fatty acids used previously, as well as the differences in the tested cell lines [41].

## 4. Conclusions

A group of platinum(IV) prodrugs of the type [Pt(P_L_)(A_L_)(COXi)(OH)]^2+^ (where P_L_ is Phen or 5,6-Me_2_Phen and A_L_ is SSDACH), all of which contain a COX inhibitor (where COXi is either indomethacin or aspirin) in the axial position, were synthesised, characterised and assessed for their biological activity. The complexes were characterised using NMR spectroscopy, HPLC, ESI-MS, UV and CD spectroscopy, as well as SRCD for **1** and **2**, which together confirmed the successful synthesis and purity of each complex. Preliminary reduction studies via ^1^H NMR of **3** showed that it reduced to its platinum(II) congener, thus confirming the successful release of its axial ligand. The lipophilicity of **1**–**4** was determined using RP-HPLC, with the produced $\log k_{w}$ values indicating that the complexes with indomethacin in the axial position (**1** and **2**) were more lipophilic than those with aspirin in the axial position (**3** and **4**).

MTT assays were used to assess the in vitro cytotoxicity of **1**–**4** against a panel of cell lines. The most cytotoxic prodrug was complex **4**, which exhibited a GI_50_ value as low as 3 nM against the Du145 prostate cancer cell line. Almost all of the prodrugs exhibited exceptional cytotoxicity when compared to traditional drugs such as cisplatin, with the most cytotoxic prodrug, **4**, exhibiting 1614-fold enhancement in activity relative to cisplatin in the HT29 colon cancer cell line. Furthermore, **1**–**4** showed that they can overcome cisplatin resistance in the ADDP (cisplatin-resistant) ovarian cancer cell line. The enzyme immunoassay showed that the indomethacin prodrugs (**1** and **2**) inhibited 29–71% of COX-2 expression at concentrations of 14 and 1.4 µM, comparable to indomethacin alone, while aspirin and the aspirin prodrugs (**3** and **4**) did not exhibit COX-2 inhibition despite being tested at higher concentrations. Platinum uptake studies showed that the prodrugs with the 56MESS core (**2** and **4**) exhibited greater uptake than those with the PHENSS core (**1** and **3**), and the aspirin complexes exhibited 1–1.6-fold greater uptake compared to the indomethacin complexes with the same core.

Overall, the prodrugs incorporating aspirin exhibited greater cytotoxicity compared to those incorporating indomethacin with the same platinum(II) core. Juxtaposed to this, the indomethacin prodrugs exhibited greater COX inhibition and increased lipophilicity. Although there was no clear correlation between lipophilicity, COX inhibition or cytotoxicity, a linear trend between increasing cellular uptake and increasing cytotoxicity was observed. While it is apparent that cellular uptake plays a role in the cytotoxicity of these prodrugs, it is not entirely clear how lipophilicity and COX inhibition influence this activity. The lack of correlation between the lipophilicity and uptake in these complexes warrants mechanistic studies into their cellular uptake mechanisms.

## 5. Patents

This work is part of Australian Provisional Patent Application 2022900110, Platinum(IV) complexes, February 2022, Western Sydney University, Sydney, Australia.

## Figures and Tables

**Table 1:** The $\log k_{w}$ of each complex with the type of platinum core and axial ligand.

Complex	Log *k*_w_	Axial Ligand	Pt(II) Congener
**3**	0.41	Aspirin	PHENSS
**4**	0.65	Aspirin	56MESS
**1**	1.08	Indomethacin	PHENSS
**2**	1.28	Indomethacin	56MESS

## Data Availability

All data relevant to the publication are included.

## Supplementary Materials

The following supporting information can be downloaded at: https://www.mdpi.com/article/10.3390/pharmaceutics14040787/s1, Table S1: The flash chromatography conditions for **1**–**4**, including flow rates, gradients and elution of complexes represented by column volume (CV). Table S2: The number of cells per well for each cell line and replicate. Figure S1: The ^1^H NMR spectrum of indomethacin-NHS ester in DMSO-d_6_ at 298 K. Insert: The chemical structure of indomethacin-NHS ester with assigned numbering. Figure S2: The COSY NMR spectrum of indomethacin-NHS ester in DMSO-d_6_ at 298 K. Figure S3: The ^13^C NMR spectrum of indomethacin-NHS ester in DMSO-d_6_ at 298 K. Insert: The chemical structure of indomethacin-NHS ester with assigned numbering. Figure S4: The ^1^H NMR spectrum of aspirin anhydride in DMSO-d_6_ at 298 K. Insert: The chemical structure of aspirin anhydride with assigned numbering. Figure S5: The COSY NMR spectrum of aspirin anhydride in DMSO-d_6_ at 298 K. Figure S6: The ^1^H NMR spectrum of [Pt(Phen)(SSDACH)(Indomethacin)(OH)](NO_3_)_2_ (1) in D_2_O at 298 K. Insert: The chemical structure of 1 with assigned numbering. Figure S7: The COSY NMR spectrum of [Pt(Phen)(SSDACH)(Indomethacin)(OH)](NO_3_)_2_ (**1**) in D_2_O at 298 K. Figure S8: The ^195^Pt NMR spectrum of [Pt(Phen)(SSDACH)(Indomethacin)(OH)](NO_3_)_2_ (**1**) in D_2_O at 298 K, showing a peak at 557 ppm. Figure S9: The ^1^H-^195^Pt HMQC NMR spectrum of [Pt(Phen)(SSDACH)(Indomethacin)(OH)](NO_3_)_2_ (**1**) in D_2_O at 298 K. Figure S10: The ^1^H NMR spectrum of [Pt(56Me_2_Phen)(SSDACH)(Indomethacin)(OH)](NO_3_)_2_ (**2**) in D_2_O at 298 K. Insert: The chemical structure of **2** with assigned numbering. Figure S11: The COSY NMR spectrum of [Pt(56Me_2_Phen)(SSDACH)(Indomethacin)(OH)](NO_3_)_2_ (**2**) in D_2_O at 298 K. Figure S12: The ^195^Pt NMR spectrum of [Pt(56Me_2_Phen)(SSDACH)(Indomethacin)(OH)](NO_3_)_2_ (**2**) in D_2_O at 298 K, showing a peak at 540 ppm. Figure S13: The ^1^H-^195^Pt HMQC NMR spectrum of [Pt(56Me_2_Phen)(SSDACH)(Indomethacin)(OH)](NO_3_)_2_ (**2**) in D_2_O at 298 K. Figure S14: The ^1^H NMR spectrum of [Pt(Phen)(SSDACH)(Aspirin)(OH)](NO_3_)_2_ (**3**) in D_2_O at 298 K. Insert: The chemical structure of **3** with assigned numbering. Figure S15: The COSY NMR spectrum of [Pt(Phen)(SSDACH)(Aspirin)(OH)](NO_3_)_2_ (**3**) in D_2_O at 298 K. Figure S16: The ^195^Pt NMR spectrum of [Pt(Phen)(SSDACH)(Aspirin)(OH)](NO_3_)_2_ (**3**) in D_2_O at 298 K, showing a peak at 536 ppm. Figure S17: The ^1^H-^195^Pt HMQC NMR spectrum of [Pt(Phen)(SSDACH)(Aspirin)(OH)](NO_3_)_2_ (**3**) in D_2_O at 298 K. Figure S18: The ^1^H NMR spectrum of [Pt(56Me_2_Phen)(SSDACH)(Aspirin)(OH)](NO_3_)_2_ (**4**) in D_2_O at 298 K. Insert: The chemical structure of **4** with assigned numbering. Figure S19: The COSY NMR spectrum of [Pt(56Me_2_Phen)(SSDACH)(Aspirin)(OH)](NO_3_)_2_ (**4**) in D_2_O at 298 K. Figure S20: The ^195^Pt NMR spectrum of [Pt(56Me_2_Phen)(SSDACH)(Aspirin)(OH)](NO_3_)_2_ (**4**) in D_2_O at 298 K, showing a peak at 525 ppm. Figure S21: The ^1^H-^195^Pt HMQC NMR spectrum of [Pt(56Me_2_Phen)(SSDACH)(Aspirin)(OH)](NO_3_)_2_ (**4**) in D_2_O at 298 K. Figure S22: The HPLC chromatogram of [Pt(Phen)(SSDACH)(Indomethacin)(OH)](NO_3_)_2_ (**1**), at 254 nm, at a gradient of 0–100% (ACN:H_2_O, 9:1) over 15 min. Figure S23: The HPLC chromatogram of [Pt(56Me_2_Phen)(SSDACH)(Indomethacin)(OH)](NO_3_)_2_ (**2**), at 254 nm, at a gradient of 0–100% (ACN:H_2_O, 9:1) over 15 min. Figure S24: The HPLC chromatogram of [Pt(Phen)(SSDACH)(Aspirin)(OH)](NO_3_)_2_ (**3**), at 254 nm, at a gradient of 0–100% (ACN:H_2_O, 9:1) over 15 min. Figure S25: The HPLC chromatogram of [Pt(56Me_2_Phen)(SSDACH)(Aspirin)(OH)](NO_3_)_2_ (**4**), at 254 nm, at a gradient of 0–100% (ACN:H_2_O, 9:1) over 15 min. Figure S26: Full ESI-MS spectrum of Indomethacin-NHS ester. Below: The expanded region of the [M + Na]^+^ peak. Figure S27: Full ESI-MS spectrum of [Pt(Phen)(SSDACH)(Indomethacin)(OH)](NO_3_)_2_ (**1**). Below: The expanded region of the [M]^+^ peak. Figure S28: Full ESI-MS spectrum of [Pt(56Me_2_Phen)(SSDACH)(Indomethacin)(OH)](NO_3_)_2_ (**2**). Below: The expanded region of the [M]^+^ peak. Figure S29: Full ESI-MS spectrum of [Pt(Phen)(SSDACH)(Aspirin)(OH)](NO_3_)_2_ (**3**). Below: The expanded region of the [M-H]^+^ peak. Figure S30: Full ESI-MS spectrum of [Pt(56Me_2_Phen)(SSDACH)(Aspirin)(OH)](NO_3_)_2_ (**4**). Below: The expanded region of the [M-H]^+^ peak. Table S3: Summary of characterisation data for **1**–**4**. Figure S31: The UV spectrum of a replicate of [Pt(Phen)(SSDACH)(Indomethacin)(OH)](NO_3_)_2_ (**1**) in water. Figure S32: The UV spectrum of a replicate of [Pt(56Me_2_Phen)(SSDACH)(Indomethacin)(OH)](NO_3_)_2_ (**2**) in water. Figure S33: The UV spectrum of a replicate of [Pt(Phen)(SSDACH)(Aspirin)(OH)](NO_3_)_2_ (**3**) in water. Figure S34: The UV spectrum of a replicate of [Pt(56Me_2_Phen)(SSDACH)(Aspirin)(OH)](NO_3_)_2_ (**4**) in water. Figure S35: The UV spectra of Phen (in water) and 56Me_2_Phen (in MeOH). Figure S36: The CD spectrum of [Pt(Phen)(SSDACH)(Indomethacin)(OH)](NO_3_)_2_ (**1**) in water. 9pt smoothing applied. Figure S37: The CD spectrum of [Pt(56Me_2_Phen)(SSDACH)(Indomethacin)(OH)](NO_3_)_2_ (**2**) in water. 9pt smoothing applied. Figure S38: The SRCD spectrum of [Pt(Phen)(SSDACH)(Indomethacin)(OH)](NO_3_)_2_ (**1**) in water. 7pt smoothing applied. Figure S39: The SRCD spectrum of [Pt(56Me_2_Phen)(SSDACH)(Indomethacin)(OH)](NO_3_)_2_ (**2**) in water. 7pt smoothing applied. Figure S40: The CD spectrum of [Pt(Phen)(SSDACH)(Aspirin)(OH)](NO_3_)_2_ (**3**) in water. 9pt smoothing applied. Figure S41: The CD spectrum of [Pt(56Me_2_Phen)(SSDACH)(Aspirin)(OH)](NO_3_)_2_ (**4**) in water. 9pt smoothing applied. Figure S42: The ^195^Pt NMR spectra of **3** with 10X PBS in D_2_O after being reduced with ascorbic acid, showing the new peak that developed in the platinum(II) region (top) and the disappearance of the peak in the platinum(IV) region (bottom). Figure S43: Plot of the concentration of organic solvent vs. $\log k_{w}$ for **1**–**4**. Table S4: The in vitro cytotoxicity values of complexes **1**–**4**, their free ligands, platinum(II) and platinum(IV) di-hydroxido precursors, cisplatin, carboplatin and oxaliplatin. GI_50_ values (nM) are reported with standard error of the mean; produced from experiments that were conducted on three separate occasions (*n* = 3); n.d. = not determined. Figure S44: Inhibition of human COX- 2 by aspirin, **3** and **4**. Figure S45: Cellular accumulation levels of **1**–**4**, their platinum(II) and platinum(IV) precursors and cisplatin against A2780 (ovarian) and ADDP (cisplatin-resistant ovarian) cancer cells that were treated with 0.1 µM for 4 h. Values are reported in ng Pt/10^6^ cells and are reported with standard error of the mean; produced from three independent experiments.
