# Supplementary material for: Cyclooxygenase-Inhibiting Platinum(IV) Prodrugs with Potent Anticancer Activity
Source: Pharmaceutics. 2022 Apr 3;14(4):787. doi: 10.3390/pharmaceutics14040787 (PMC9029360; doi:10.3390/pharmaceutics14040787)
Supplement: Supplementary file 1 [file pharmaceutics-14-00787-s001.zip › pharmaceutics-1636057-supplementary.pdf]

Supplementary Materials: Cyclooxygenase-Inhibiting Platinum(IV) Prodrugs with Potent Anticancer Activity

Aleen Khoury, Jennette A. Sakoff, Jayne Gilbert, Kieran F. Scott, Shawan Karan, Christopher P. Gordon and Janice R. Aldrich-Wright

1. Flash Chromatography

Table S1. The flash chromatography conditions for **1–4**, including flow rates, gradients and elution of complexes represented by column volume (CV).

Complex	Flowrate (mL/min)	Gradient (% MeOH (B)) over various CVs	Elution of complex (CV)
[Pt(Phen)(SSDACH)(Indomethacin)(OH)]$(NO_3)_2$ (**1**)	4	0% B for 4 CV<br>0–35% B for 3 CV<br>35–42% B for 5 CV	9–11
[Pt(56Me_2Phen)(SSDACH)(Indomethacin)(OH)]$(NO_3)_2$ (**2**)	4	0% B for 8 CV<br>0–35% B for 3 CV<br>35–42% B for 6 CV	14–16
[Pt(Phen)(SSDACH)(Aspirin)(OH)]$(NO_3)_2$ (**3**)	4	0% B for 6 CV<br>0–20% B for 5 CV	8–10
[Pt(56Me_2Phen)(SSDACH)(Aspirin)(OH)]$(NO_3)_2$ (**4**)	4	0% B for 10 CV<br>0–20% B for 6 CV	13–15

2. Cellular Accumulation

Table S2. The number of cells per well for each cell line and replicate.

Replicate	Cell line	Number of cells per well
1	A2780	3.20×10^5
	ADDP	3.05×10^5
2	A2780	3.55×10^5
	ADDP	3.60×10^5
3	A2780	4.50×10^5
	ADDP	4.80×10^5

3. NMR Spectra

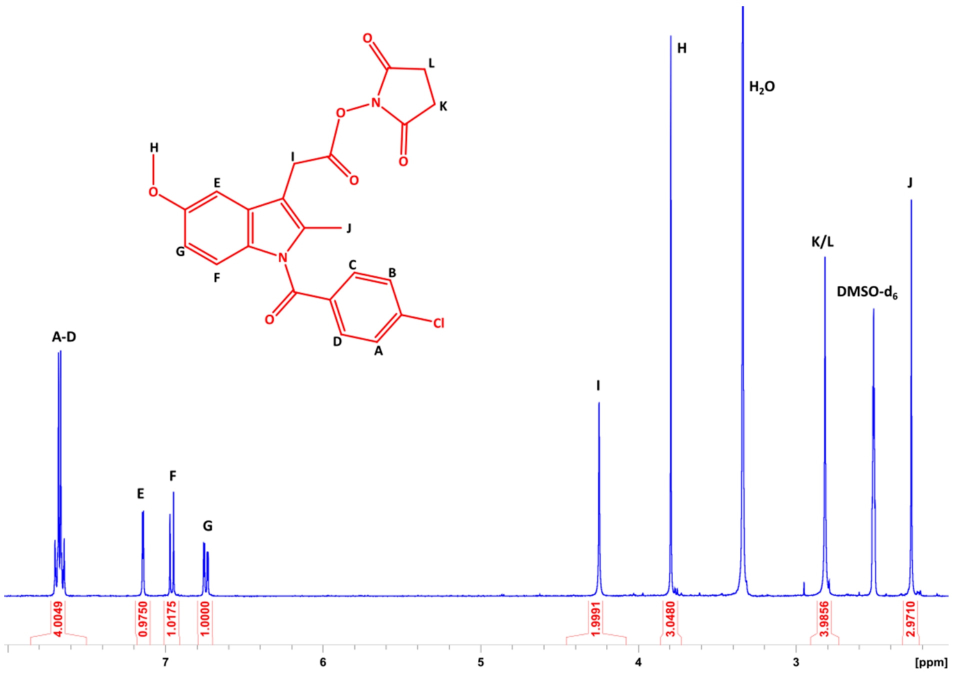

Figure S1. The ^{1}H NMR spectrum of indomethacin-NHS ester in DMSO-d6 at 298 K. Insert: The chemical structure of indomethacin-NHS ester with assigned numbering.

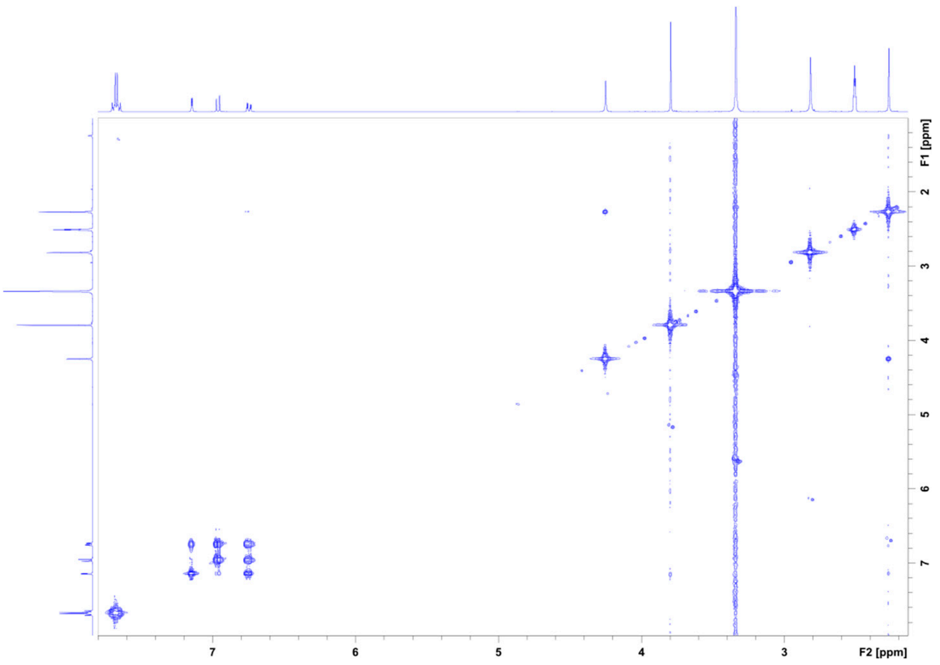

Figure S2. The COSY NMR spectrum of indomethacin-NHS ester in DMSO-d6 at 298 K.

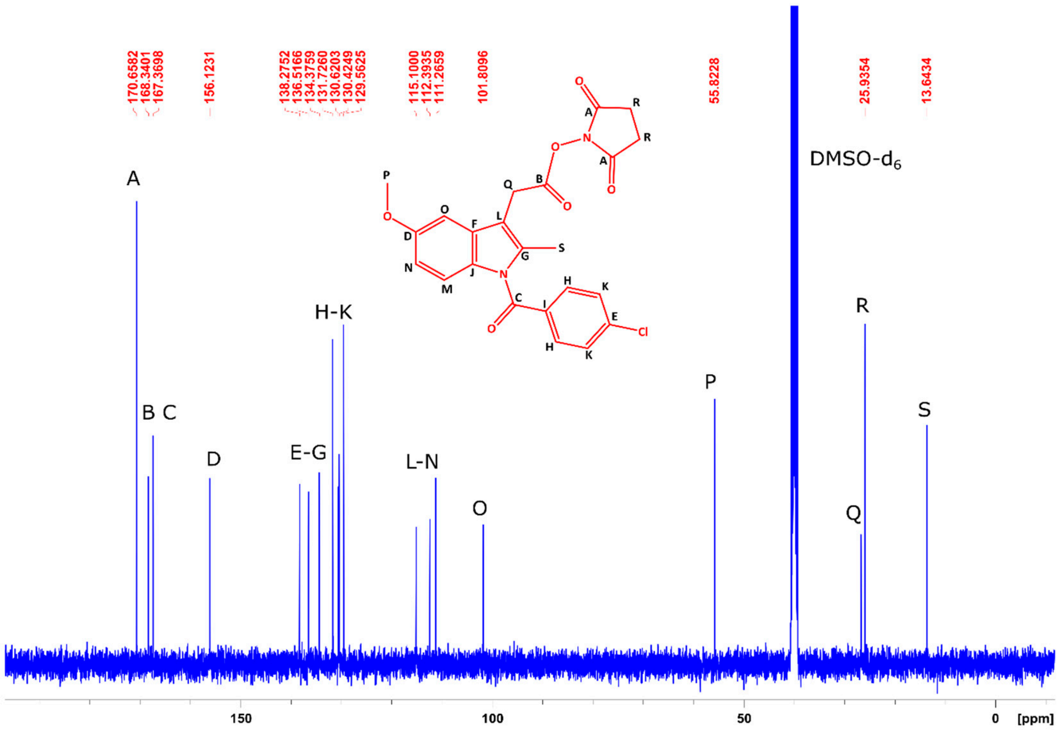

Figure S3. The ^{13}C NMR spectrum of indomethacin-NHS ester in DMSO-d₆ at 298 K. Insert: The chemical structure of indomethacin-NHS ester with assigned numbering.

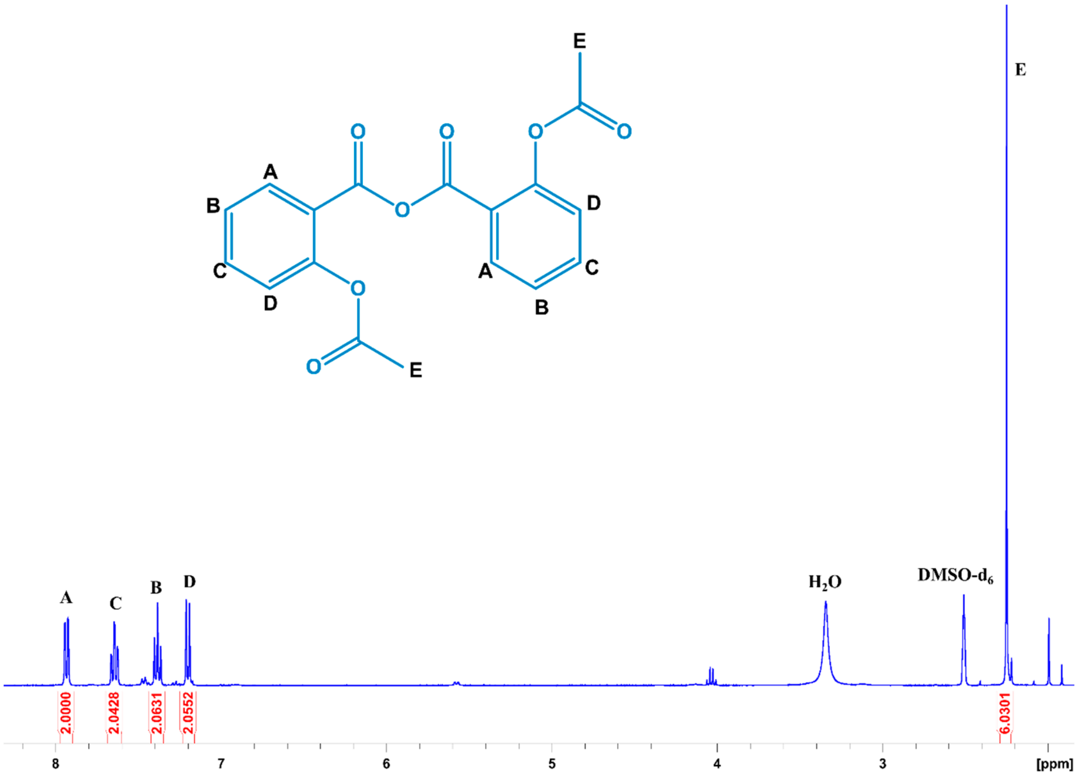

Figure S4. The ^{1}H NMR spectrum of aspirin anhydride in DMSO-d₆ at 298 K. Insert: The chemical structure of aspirin anhydride with assigned numbering.

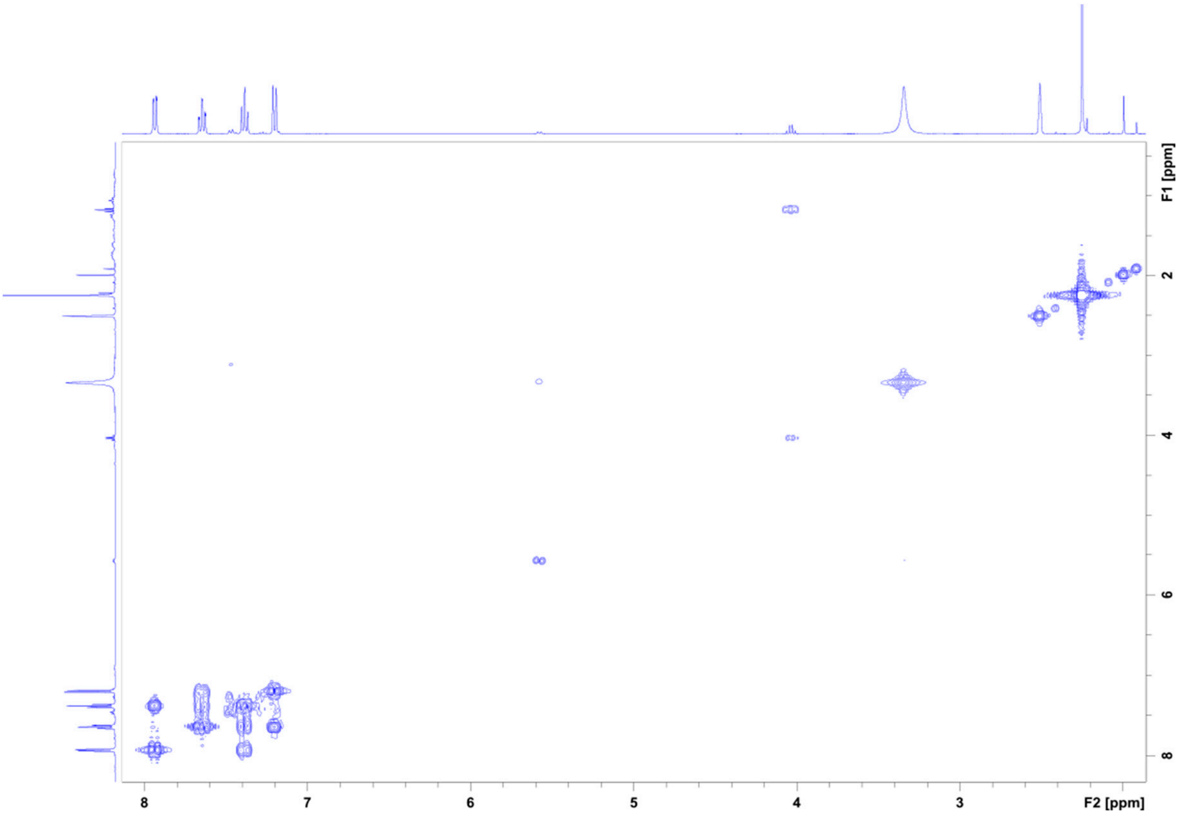

Figure S5. The COSY NMR spectrum of aspirin anhydride in DMSO-d6 at 298 K.

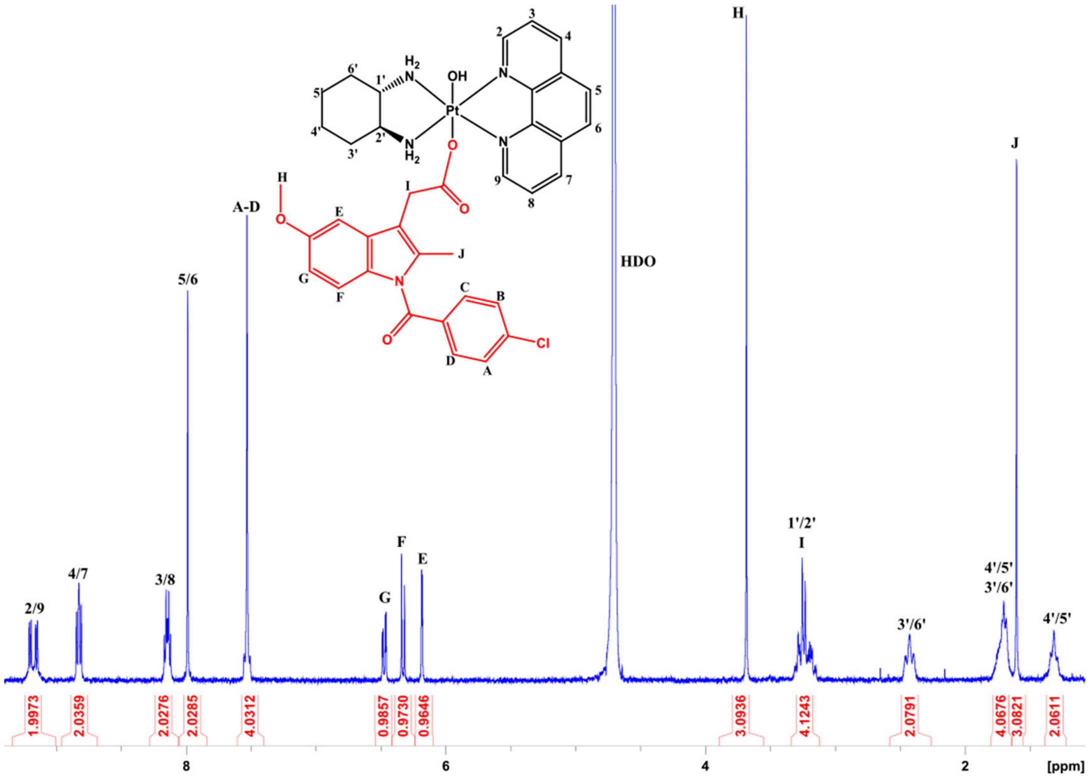

Figure S6. The ^{1}H NMR spectrum of [Pt(Phen)(SSDACH)(Indomethacin)(OH)]$(NO_3)_2$ (1) in D_2O at 298 K. Insert: The chemical structure of 1 with assigned numbering.

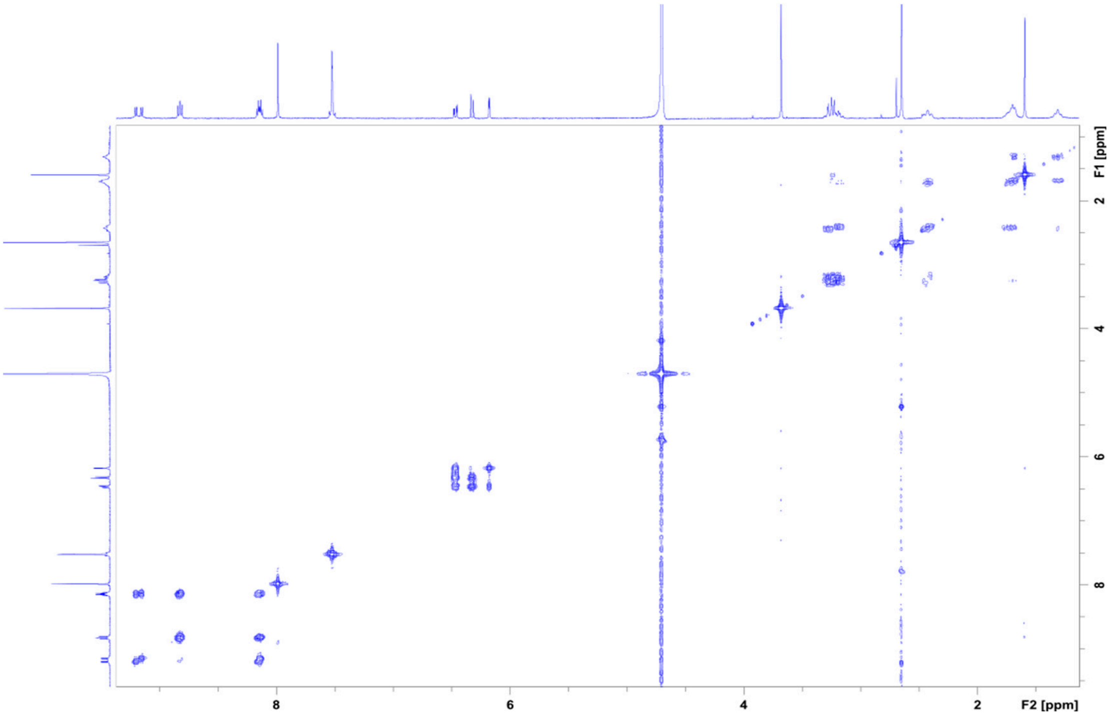

Figure S7. The COSY NMR spectrum of [Pt(Phen)(SSDACH)(Indomethacin)(OH)]$(NO_3)_2$ (**1**) in D_2O at 298 K.

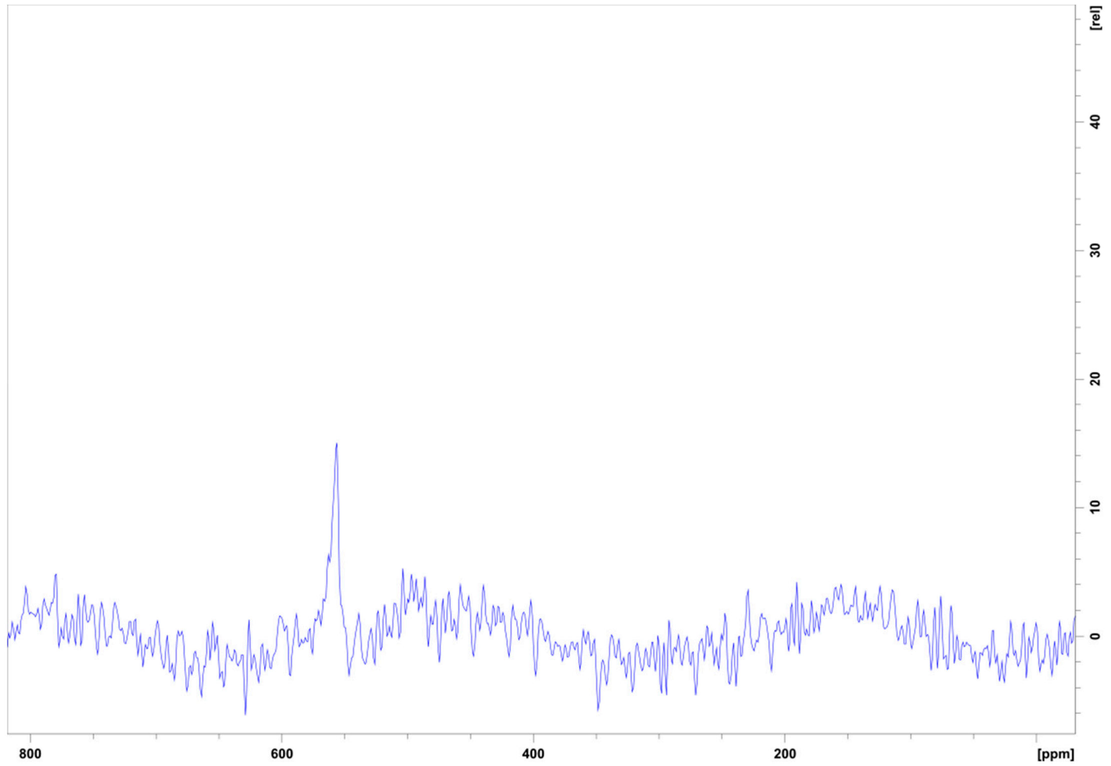

Figure S8. The ^{195}Pt NMR spectrum of [Pt(Phen)(SSDACH)(Indomethacin)(OH)]$(NO_3)_2$ (**1**) in D_2O at 298 K, showing a peak at 557 ppm.

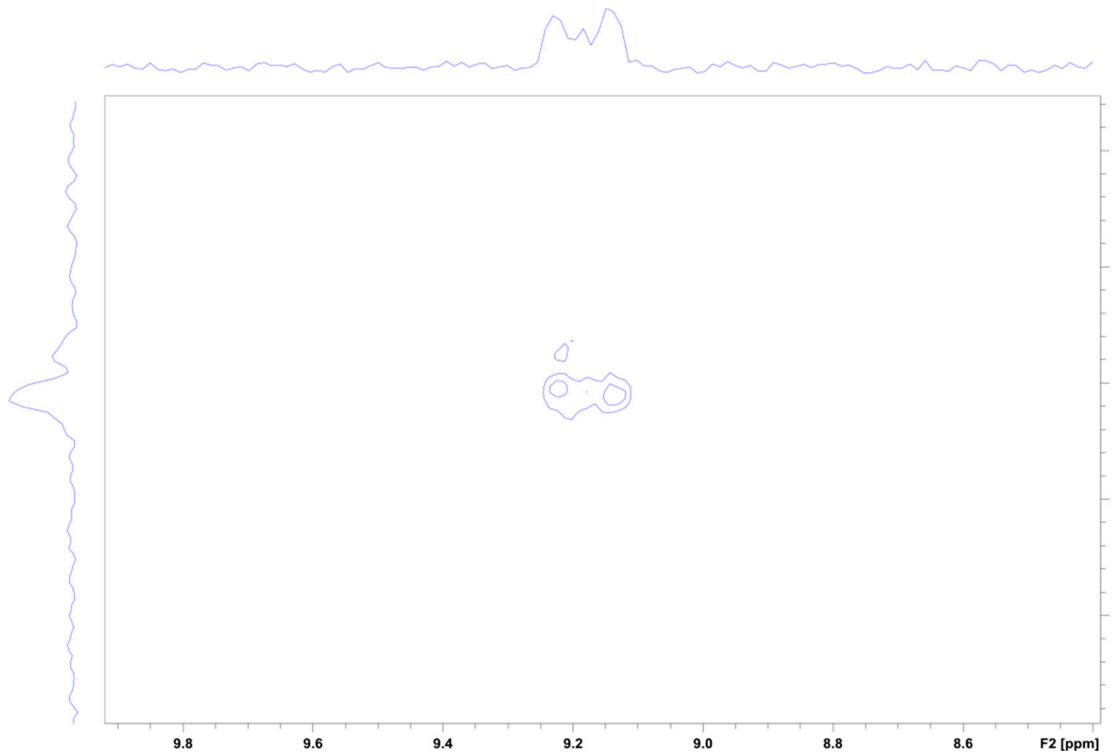

Figure S9. The ^{1}H-^{195}Pt HMQC NMR spectrum of [Pt(Phen)(SSDACH)(Indomethacin)(OH)]$(NO_3)_2$ (**1**) in D_2O at 298 K.

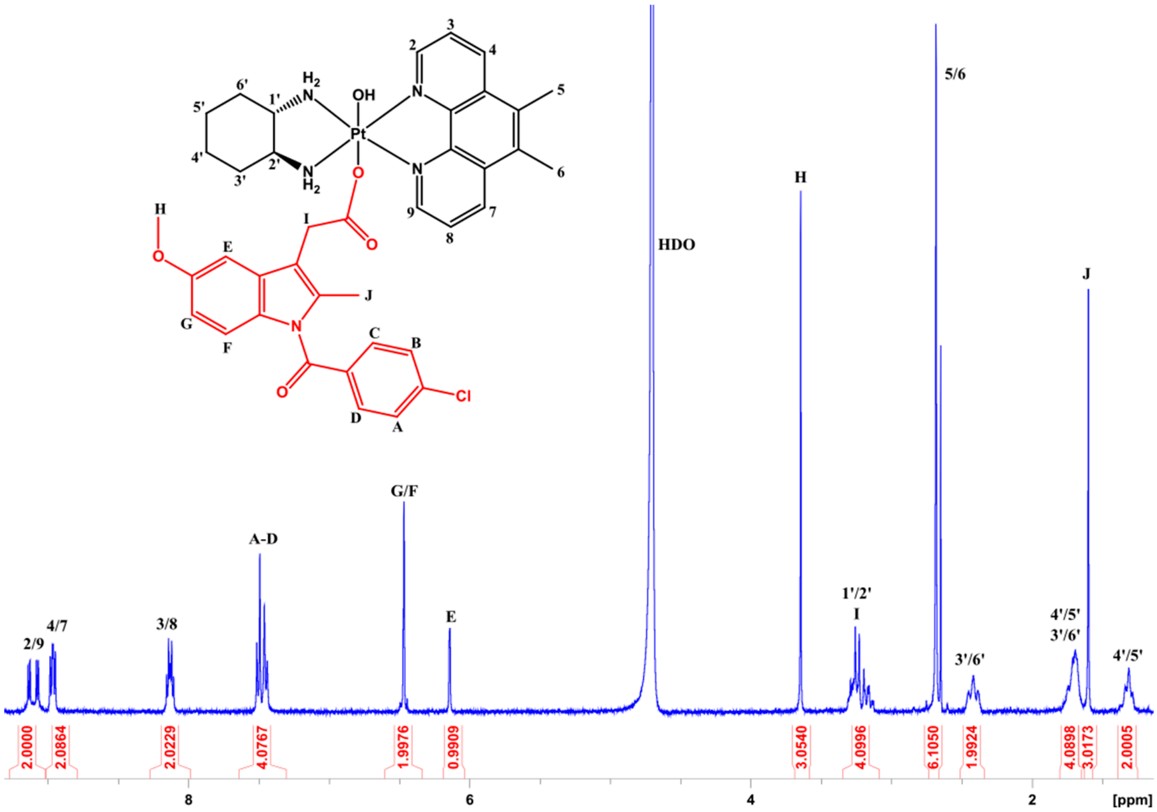

Figure S10. The ^{1}H NMR spectrum of [Pt(56Me_2Phen)(SSDACH)(Indomethacin)(OH)]$(NO_3)_2$ (**2**) in D_2O at 298 K. Insert: The chemical structure of **2** with assigned numbering.

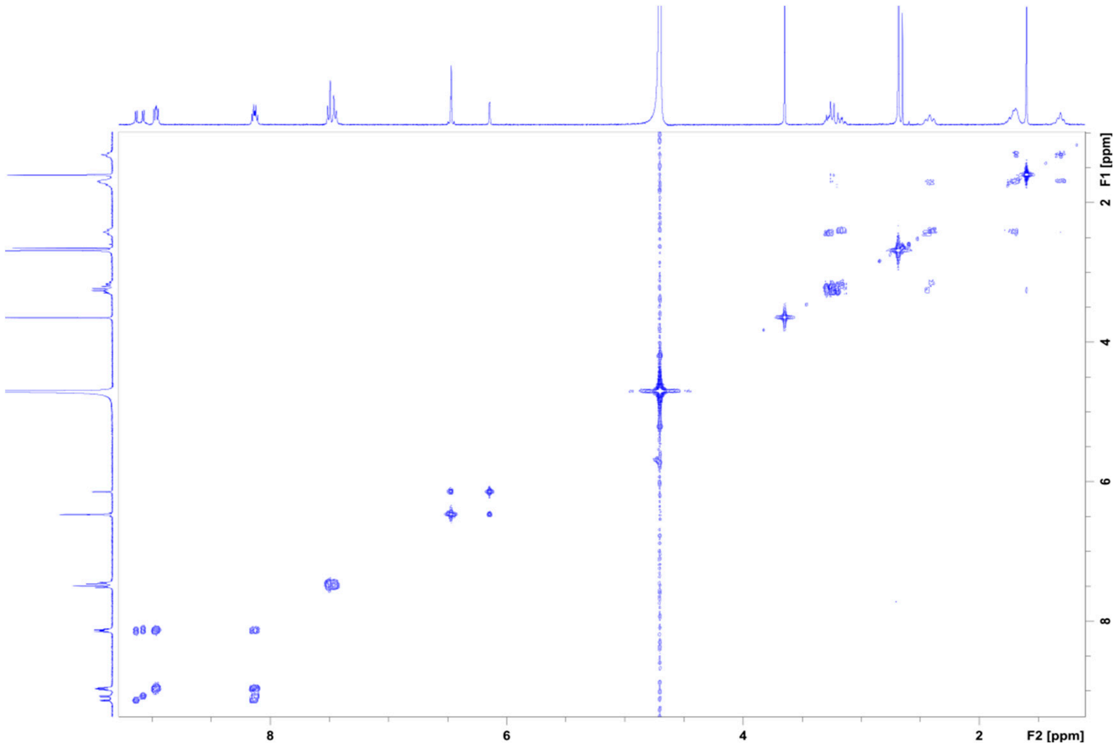

Figure S11. The COSY NMR spectrum of [Pt(56Me$_2$Phen)(SSDACH)(Indomethacin)(OH)](NO$_3$)$_2$ (**2**) in D$_2$O at 298 K.

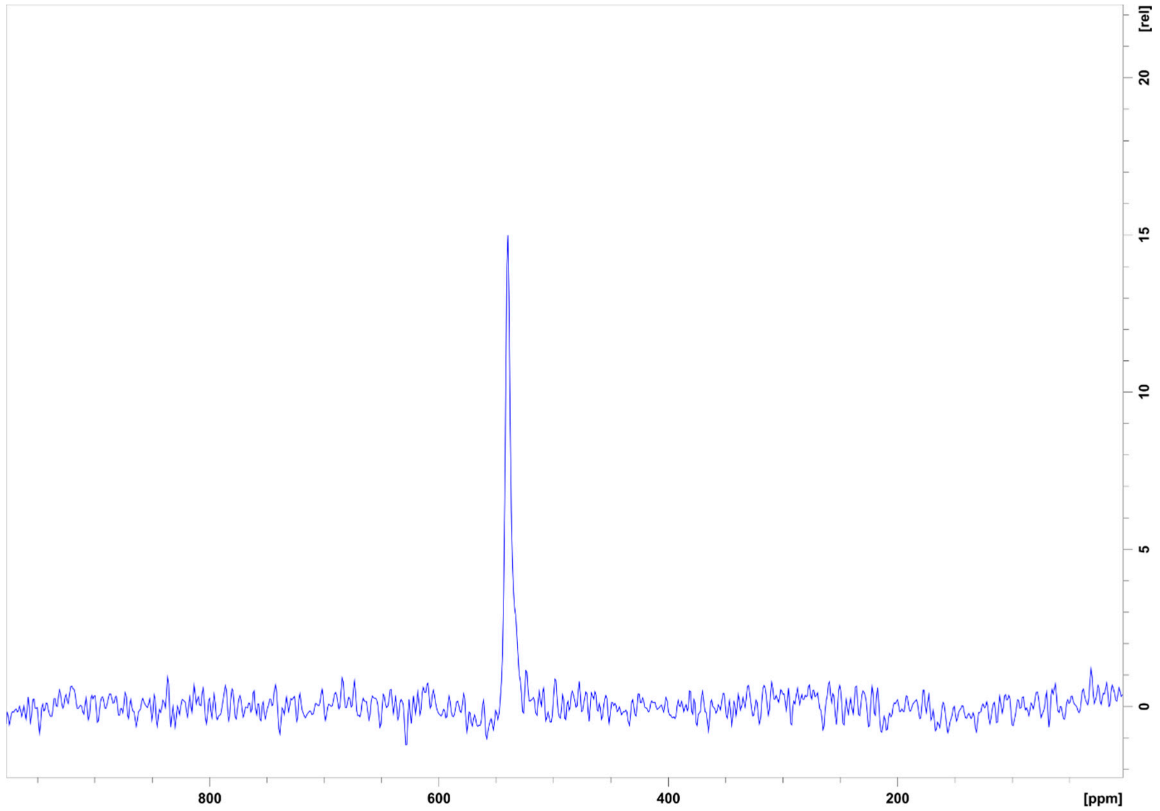

Figure S12. The ^{195}Pt NMR spectrum of [Pt(56Me$_2$Phen)(SSDACH)(Indomethacin)(OH)](NO$_3$)$_2$ (**2**) in D$_2$O at 298 K, showing a peak at 540 ppm.

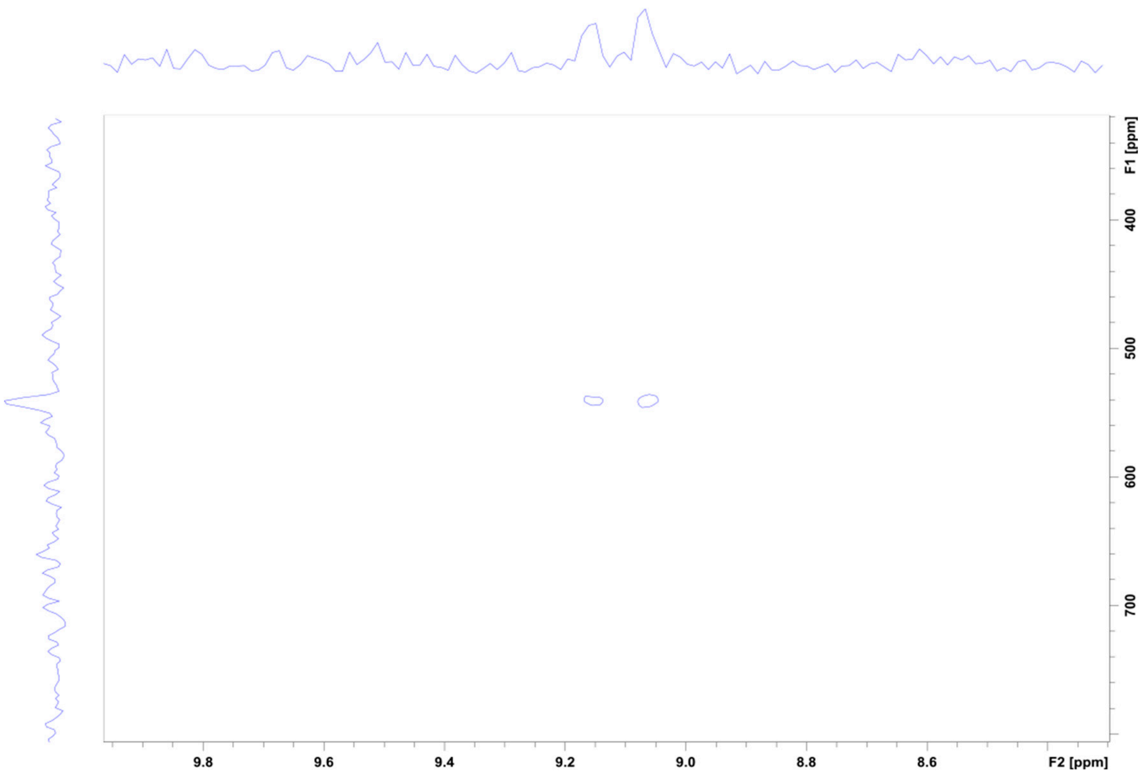

Figure S13. The ^{1}H-^{195}Pt HMQC NMR spectrum of [Pt(56Me$_2$Phen)(SSDACH)(Indomethacin)(OH)](NO_3)$_2$ (**2**) in D_2O at 298 K.

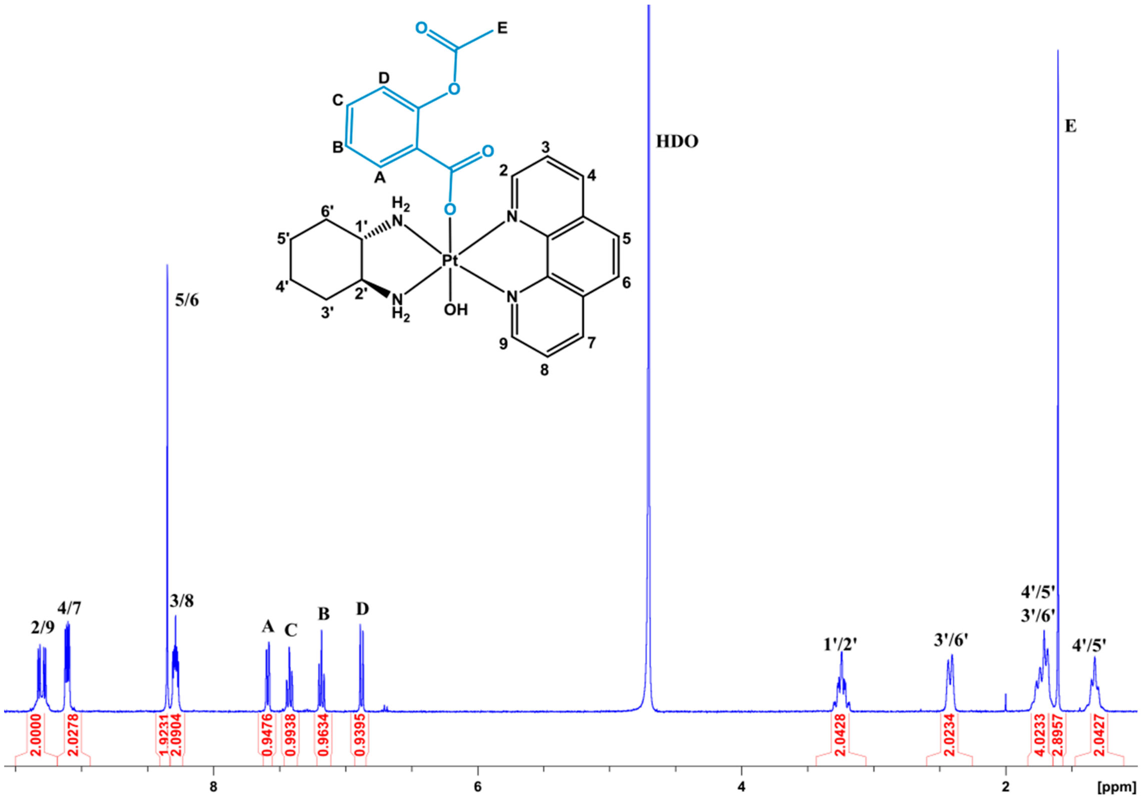

Figure S14. The ^{1}H NMR spectrum of [Pt(Phen)(SSDACH)(Aspirin)(OH)](NO_3)$_2$ (**3**) in D_2O at 298 K. Insert: The chemical structure of **3** with assigned numbering.

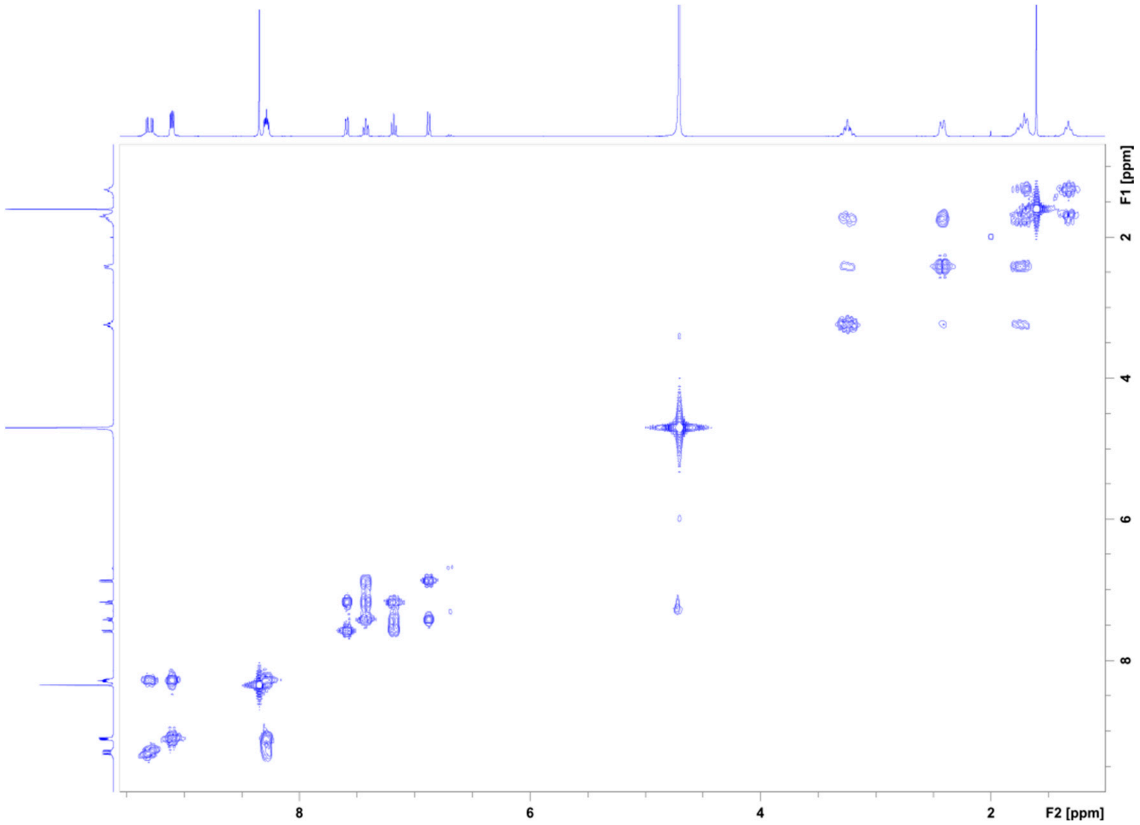

Figure S15. The COSY NMR spectrum of [Pt(Phen)(SSDACH)(Aspirin)(OH)]$(NO_3)_2$ (**3**) in D_2O at 298 K.

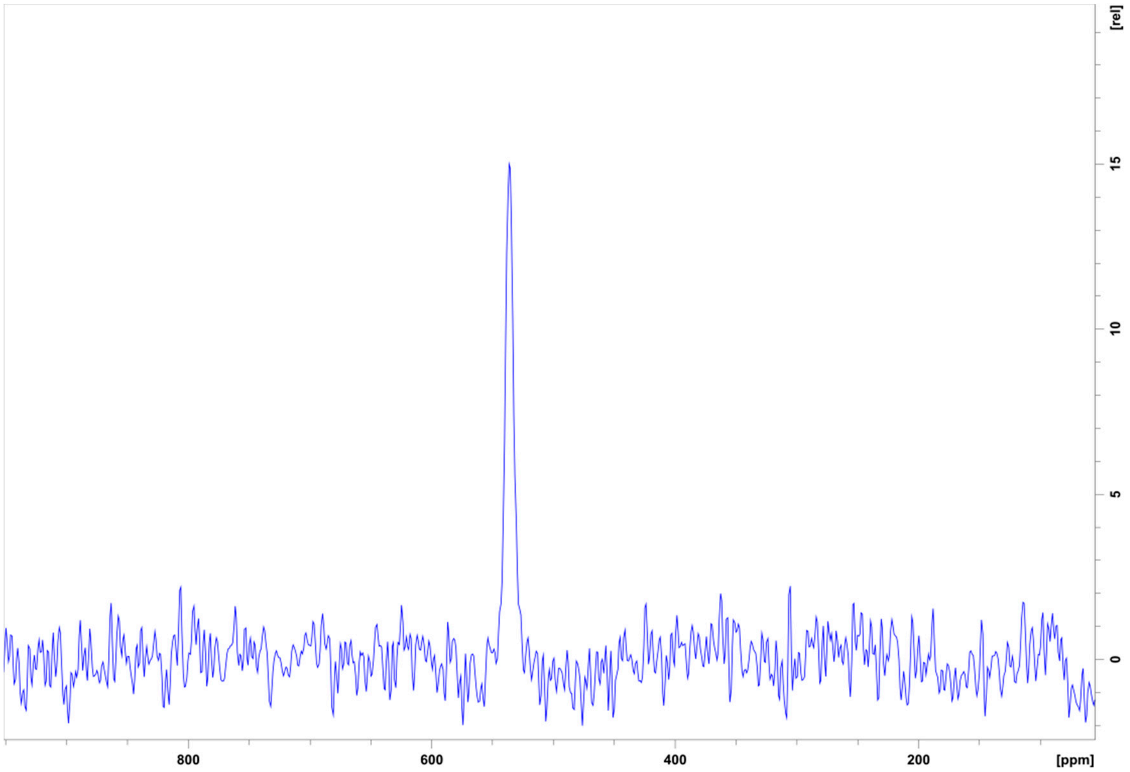

Figure S16. The ^{195}Pt NMR spectrum of [Pt(Phen)(SSDACH)(Aspirin)(OH)]$(NO_3)_2$ (**3**) in D_2O at 298 K, showing a peak at 536 ppm.

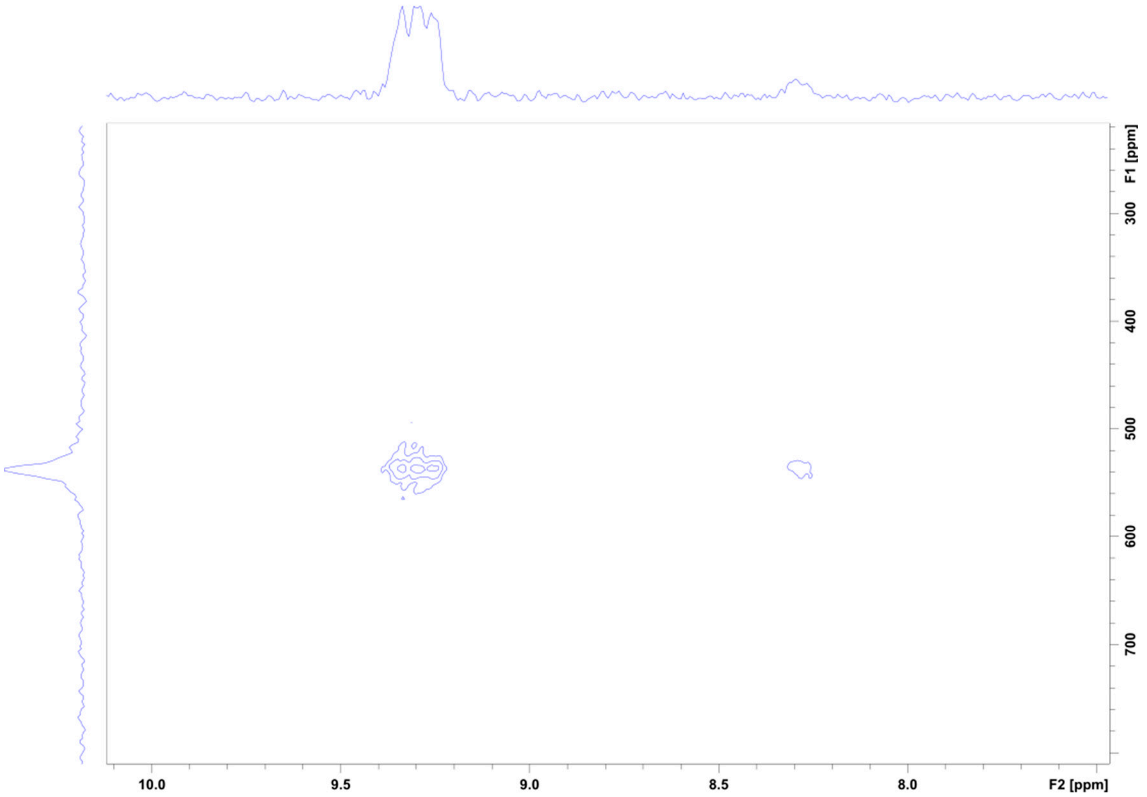

Figure S17. The ^{1}H-^{195}Pt HMQC NMR spectrum of $[Pt(Phen)(SSDACH)(Aspirin)(OH)](NO_3)_2$ (**3**) in D_2O at 298 K.

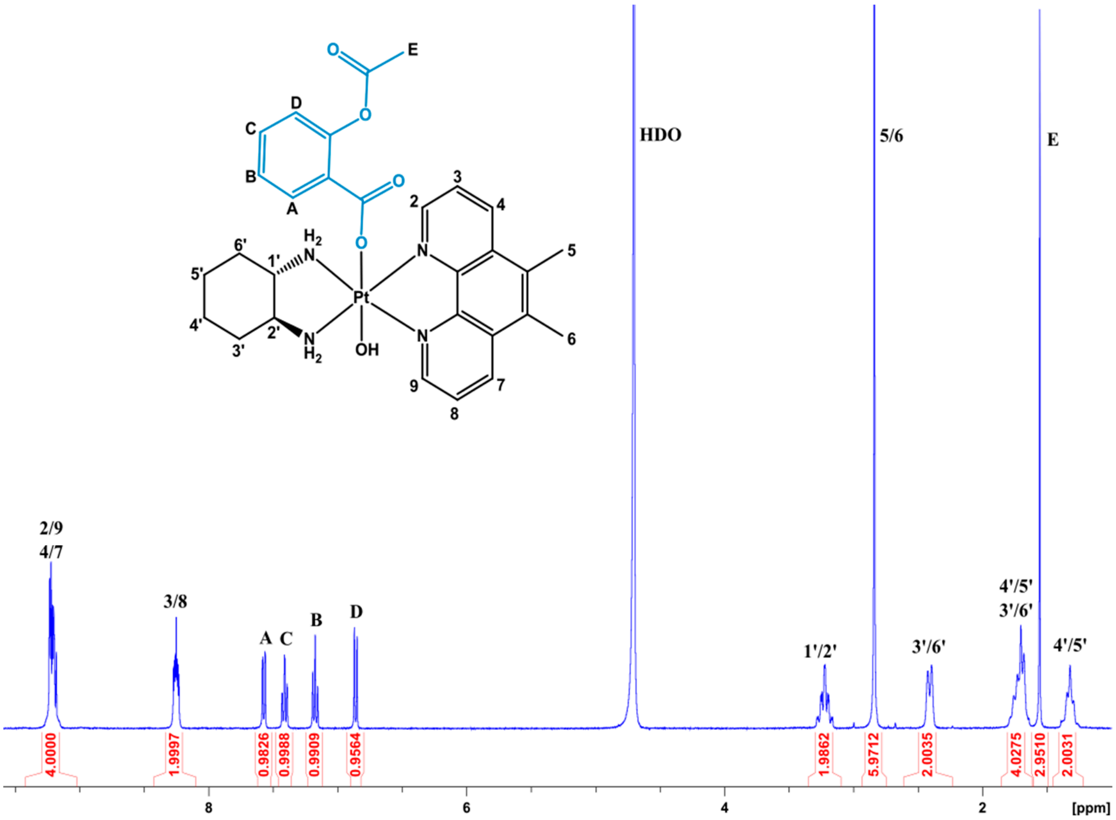

Figure S18. The ^{1}H NMR spectrum of $[Pt(56Me_2Phen)(SSDACH)(Aspirin)(OH)](NO_3)_2$ (**4**) in D_2O at 298 K. Insert: The chemical structure of **4** with assigned numbering.

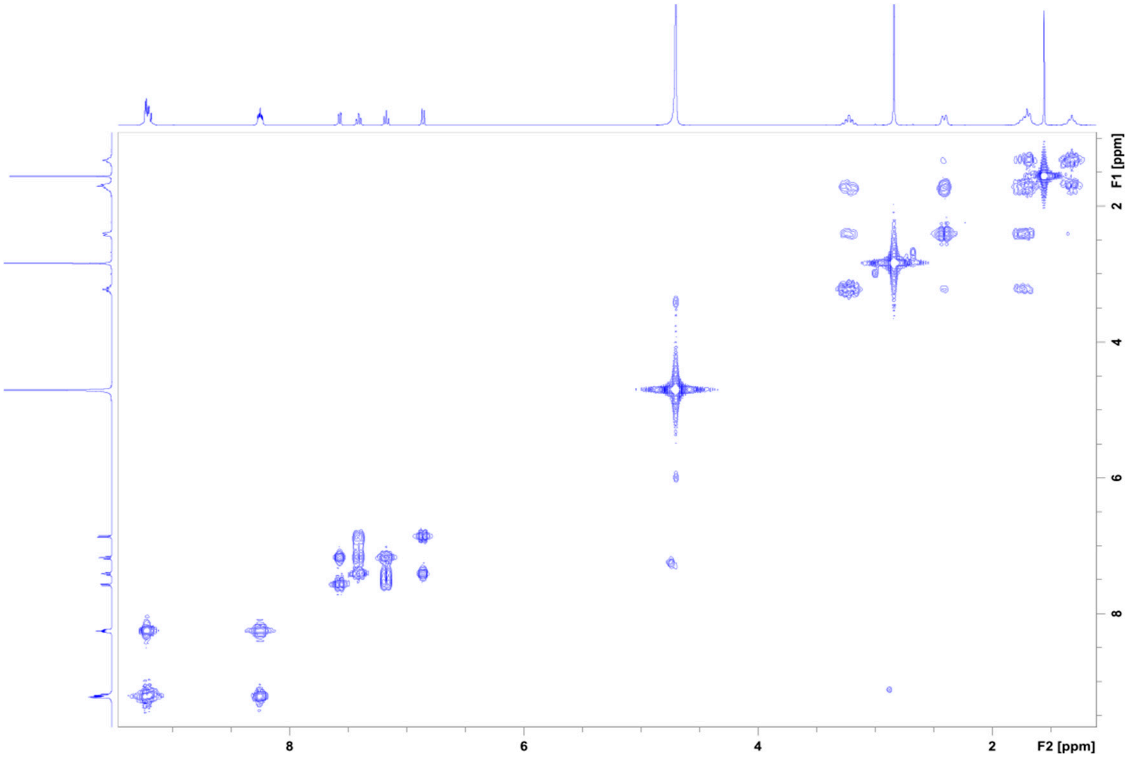

Figure S19. The COSY NMR spectrum of [Pt(56Me_2Phen)(SSDACH)(Aspirin)(OH)](NO_3)$_2$ (**4**) in D_2O at 298 K.

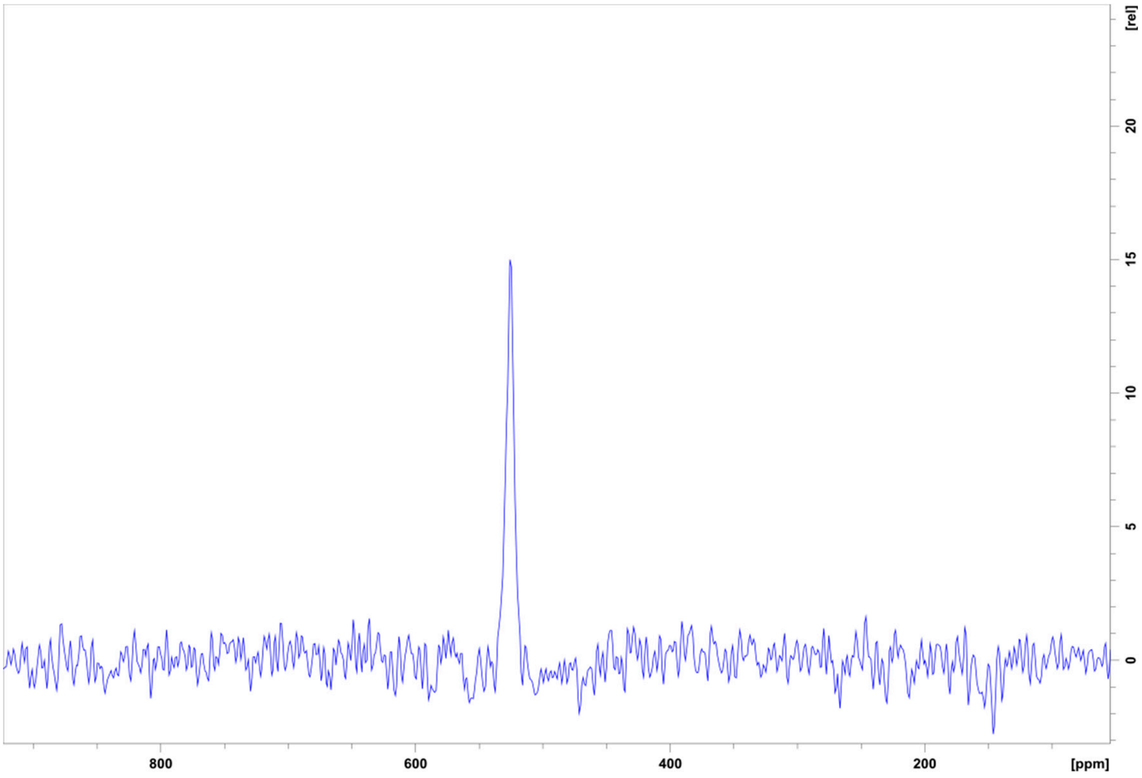

Figure S20. The ^{195}Pt NMR spectrum of [Pt(56Me_2Phen)(SSDACH)(Aspirin)(OH)](NO_3)$_2$ (**4**) in D_2O at 298 K, showing a peak at 525 ppm.

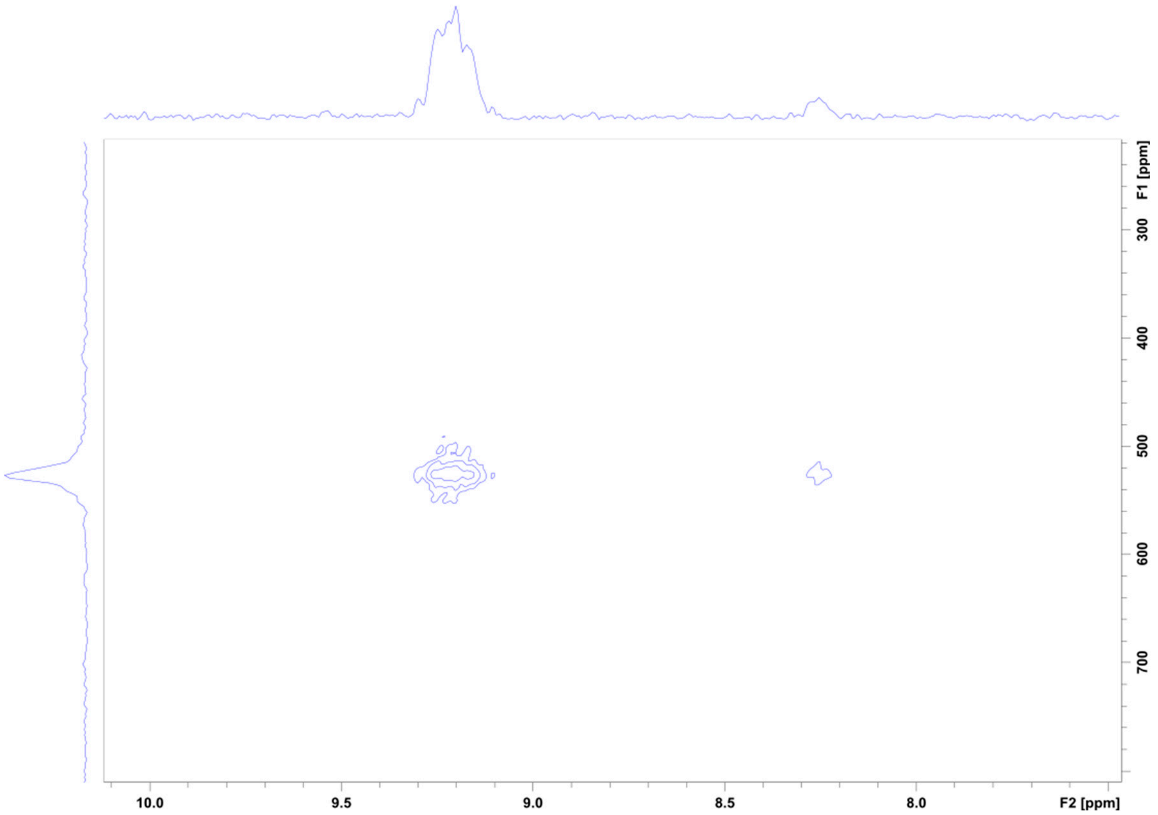

Figure S21. The ^{1}H-^{195}Pt HMQC NMR spectrum of [Pt(56Me$_2$Phen)(SSDACH)(Aspirin)(OH)](NO_3)$_2$ (**4**) in D_2O at 298 K.

4. HPLC

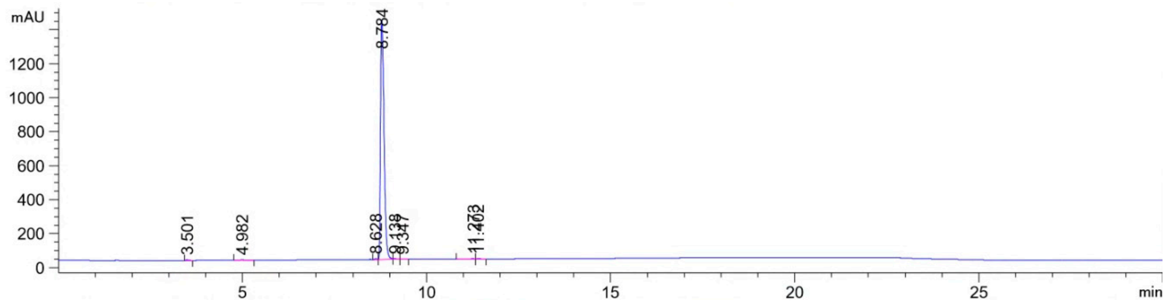

Figure S22. The HPLC chromatogram of [Pt(Phen)(SSDACH)(Indomethacin)(OH)](NO_3)$_2$ (**1**), at 254 nm, at a gradient of 0–100% (ACN:H_2O, 9:1) over 15 min.

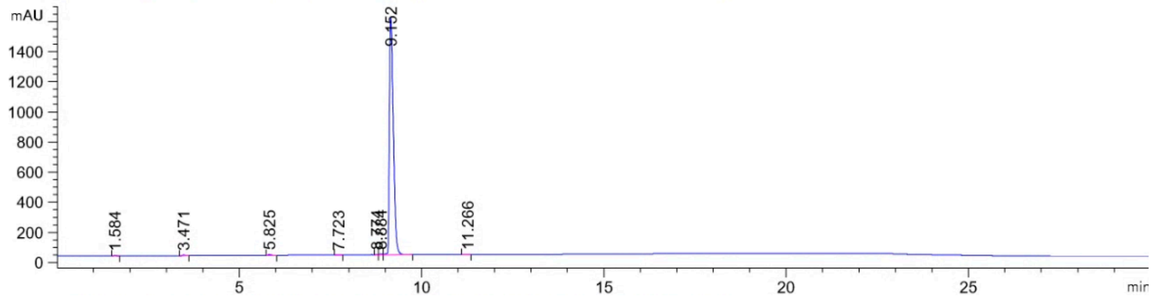

Figure S23. The HPLC chromatogram of [Pt(56Me$_2$Phen)(SSDACH)(Indomethacin)(OH)](NO_3)$_2$ (**2**), at 254 nm, at a gradient of 0–100% (ACN:H_2O, 9:1) over 15 min.

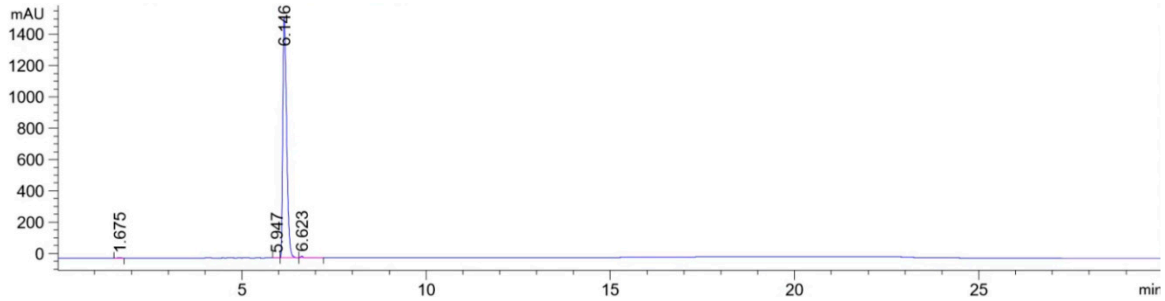

Figure S24. The HPLC chromatogram of [Pt(Phen)(SSDACH)(Aspirin)(OH)](NO_3)$_2$ (**3**), at 254 nm, at a gradient of 0–100% (ACN:H_2O, 9:1) over 15 min.

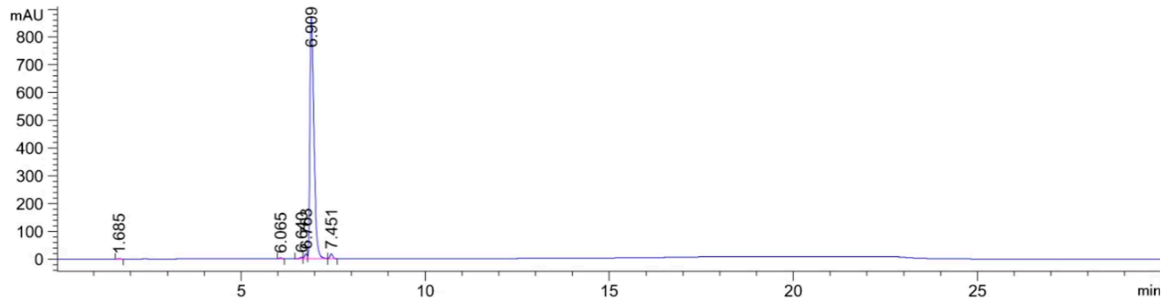

Figure S25. The HPLC chromatogram of [Pt(56Me$_2$Phen)(SSDACH)(Aspirin)(OH)](NO_3)$_2$ (**4**), at 254 nm, at a gradient of 0–100% (ACN:H_2O, 9:1) over 15 min.

5. ESI-MS

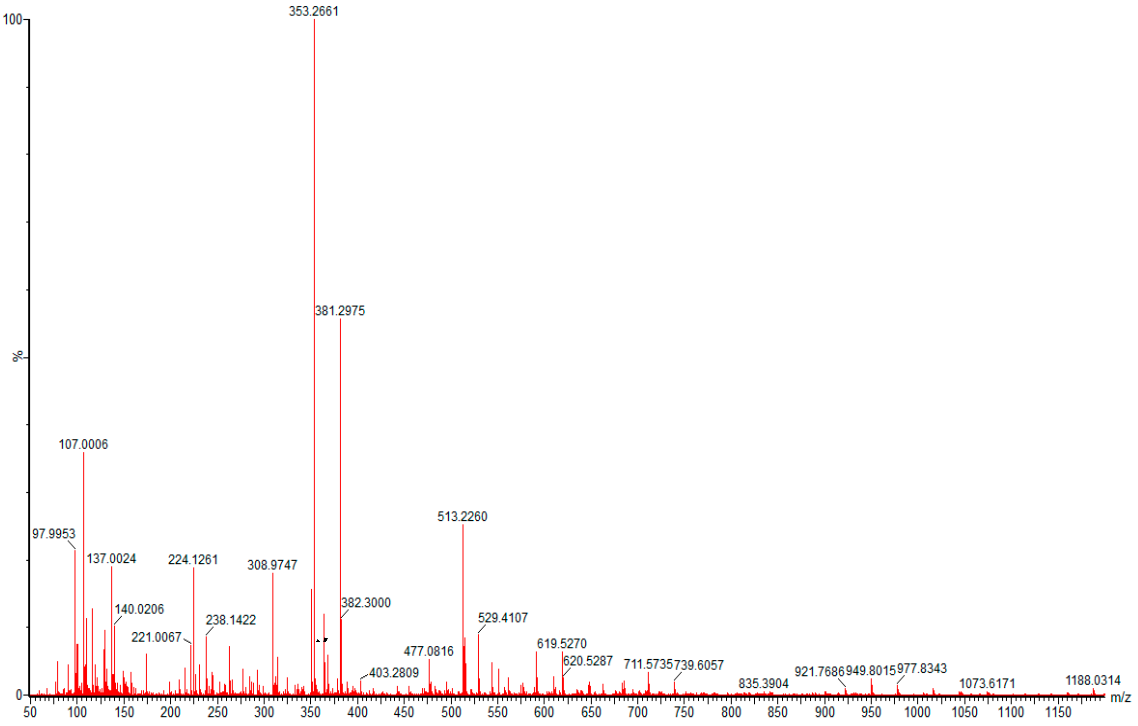

Figure S26. Full ESI-MS spectrum of Indomethacin-NHS ester. Below: The expanded region of the [M+Na]$^+$ peak.

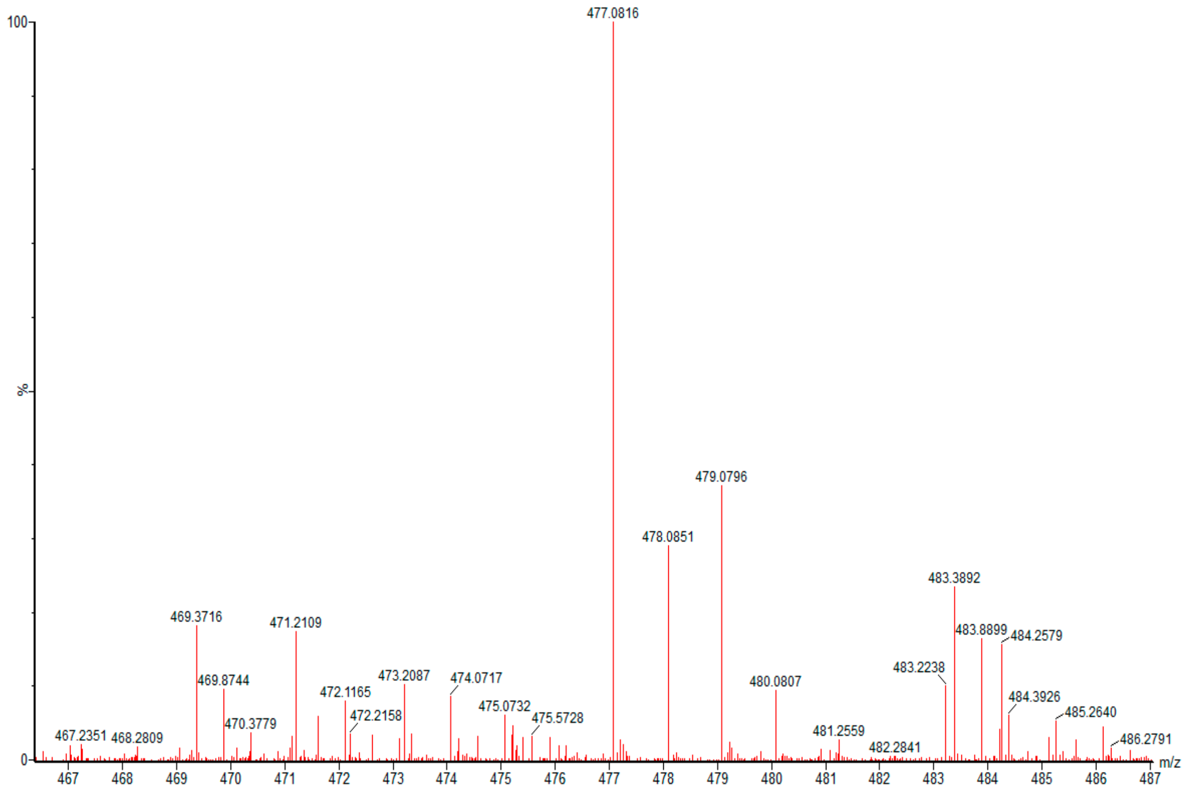

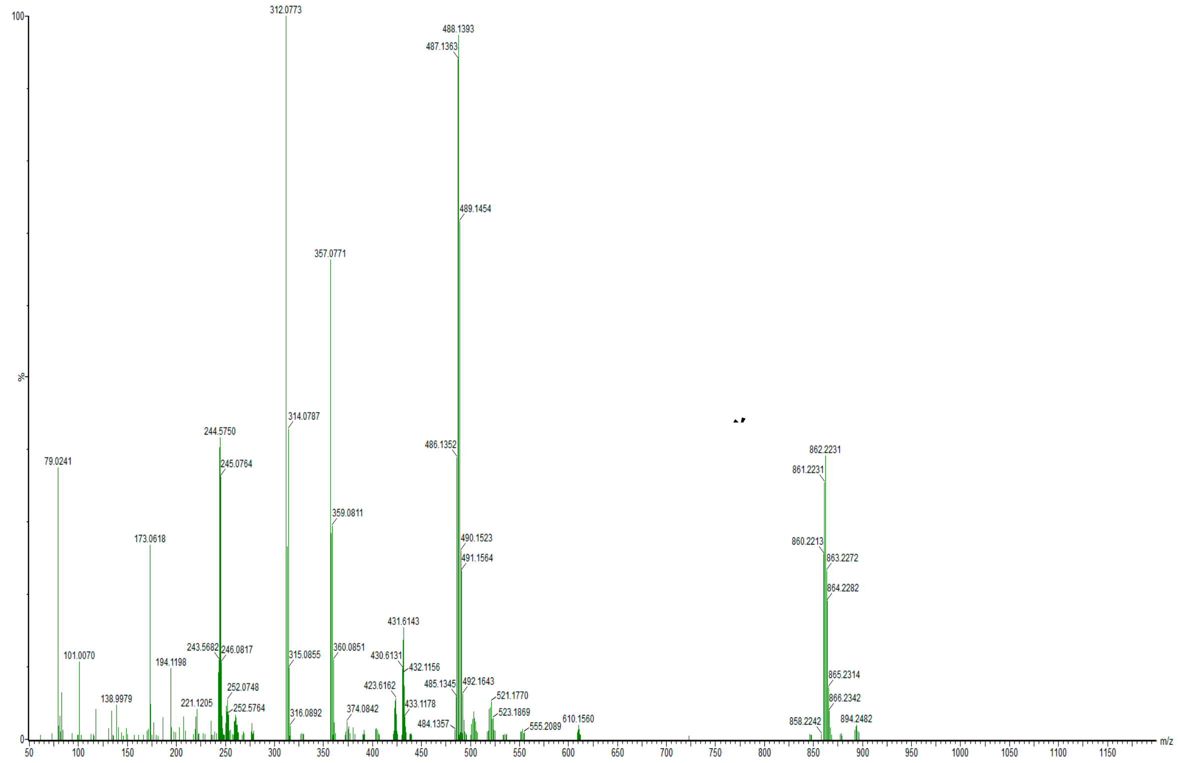

Figure S27. Full ESI-MS spectrum of [Pt(Phen)(SSDACH)(Indomethacin)(OH)]$(NO_3)_2$ (**1**). Below: The expanded region of the $[M]^+$ peak.

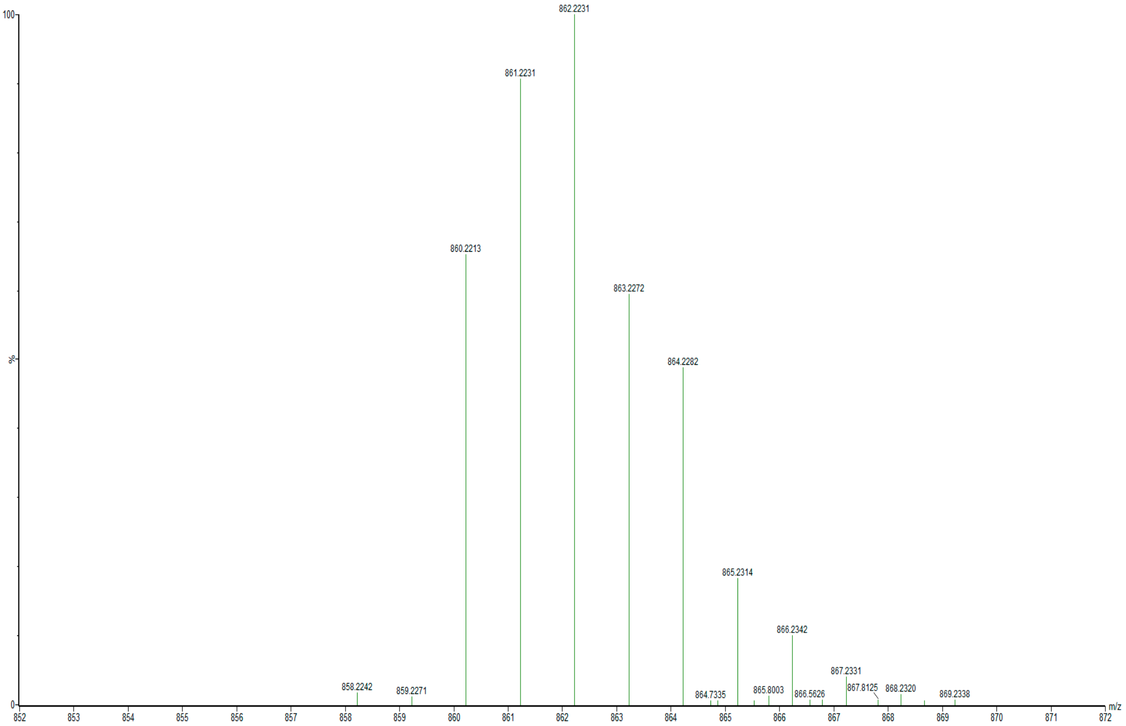

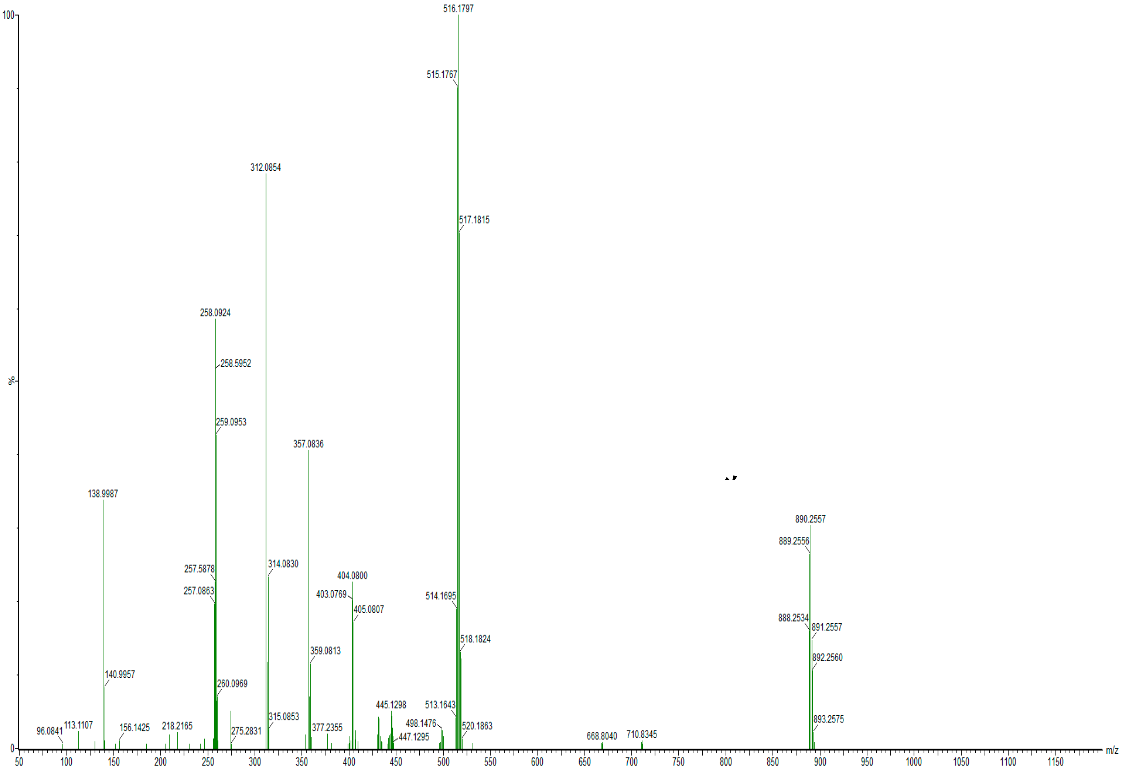

Figure S28. Full ESI-MS spectrum of [Pt(56Me_2Phen)(SSDACH)(Indomethacin)(OH)]$(NO_3)_2$ (**2**). Below: The expanded region of the $[M]^+$ peak.

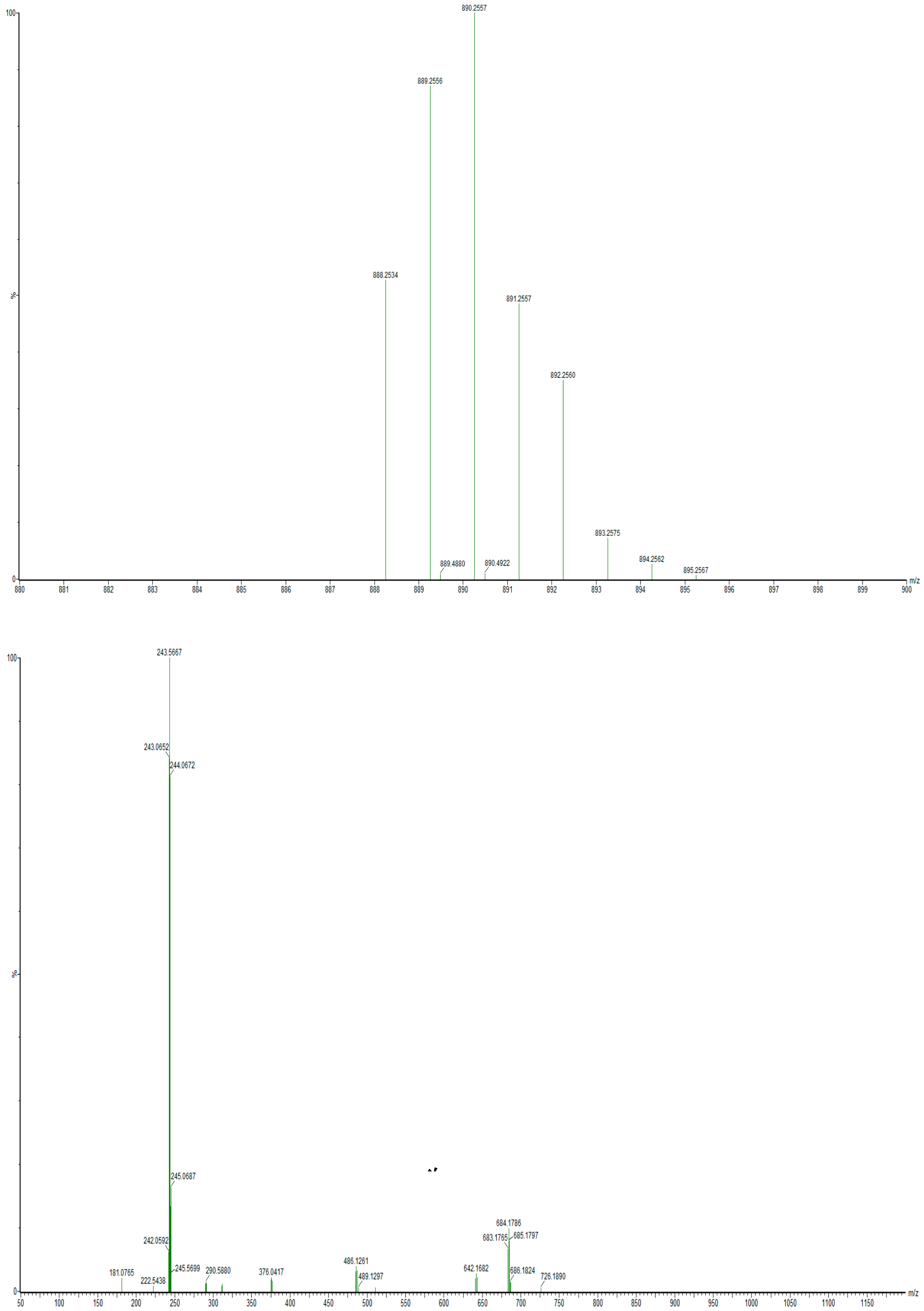

Figure S29. Full ESI-MS spectrum of [Pt(Phen)(SSDACH)(Aspirin)(OH)](NO_3)$_2$ (**3**). Below: The expanded region of the [M-H]$^+$ peak.

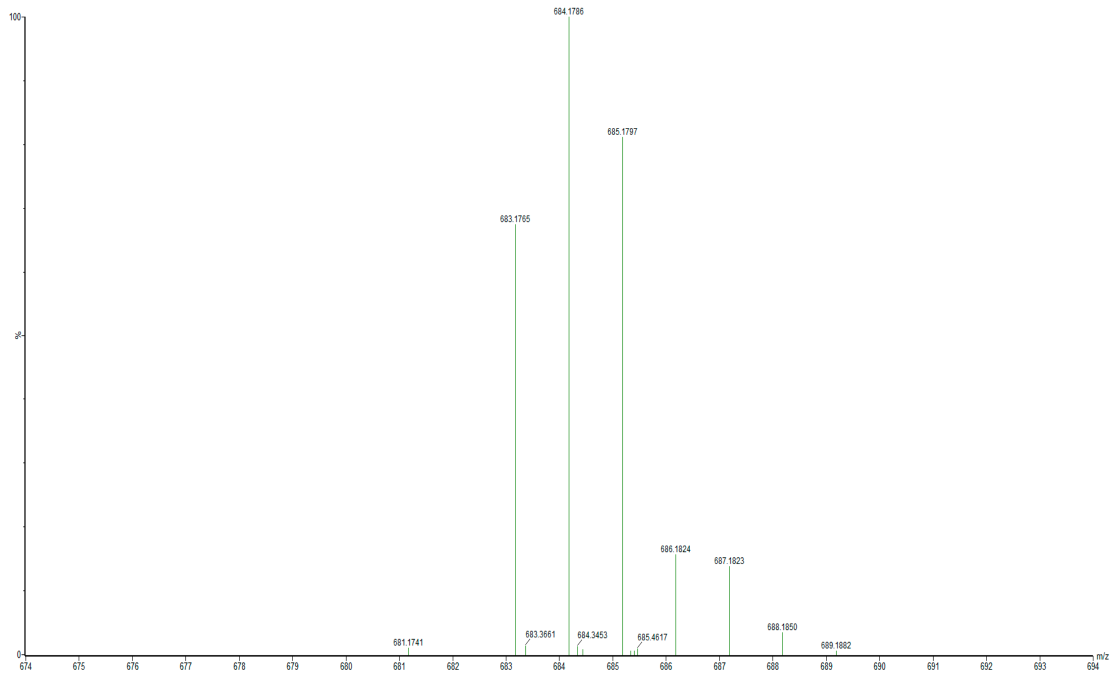

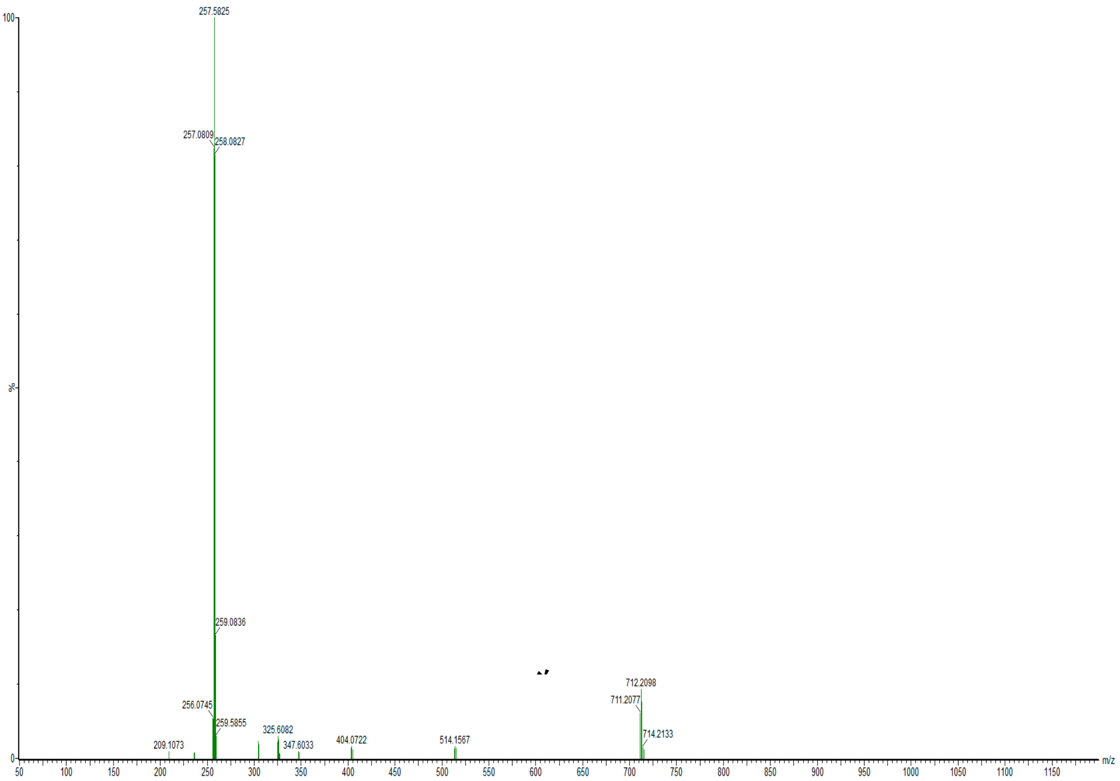

Figure S30. Full ESI-MS spectrum of [Pt(56Me$_2$Phen)(SSDACH)(Aspirin)(OH)](NO_3)$_2$ (**4**). Below: The expanded region of the [M-H]$^+$ peak.

6. Summary of Characterisation Data

Table S3. Summary of characterisation data for **1**–**4**.

Complex	Molecular Formula	Yield (%)	HRMS-ESI Calc. (found)	Electronic spectrum λ_{max} nm (ε/M^{-1} cm^{-1})	CD λ_{max} nm (mdeg mol L^{-1})	HPLC purity (%)
**1**	$C_{37}H_{38}ClN_7O_{11}Pt$	65	$[M]^+$ *m/z* = 862.2209 (862.2231)	201 (161,400 ± 430), 276 (50,000 ± 115), 303 (16,650 ± 120)	206 (-2.8), 267 (0.3), 290 (-0.4)	98.8
**2**	$C_{39}H_{42}ClN_7O_{11}Pt$	63	$[M]^+$ *m/z* = 890.2522 (890.2557)	202 (193,500 ± 215), 282 (51,600 ± 145), 313 (19,900 ± 245)	210 (-2.9), 242 (-1.2), 274 (0.7), 295 (-0.9)	99.1
**3**	$C_{27}H_{30}N_6O_{11}Pt$	69	$[M\text{-}H]^+$ *m/z* = 684.1786 (684.1786)	202 (94,900 ± 295), 279 (28,900 ± 75), 306 (8050 ± 40)	215 (-2.3)	99.3
**4**	$C_{29}H_{34}N_6O_{11}Pt$	67	$[M\text{-}H]^+$ *m/z* = 712.2099 (712.2098)	200 (108,200 ± 330), 291 (32,200 ± 105), 317 (7850 ± 25)	216 (-2.1)	96.8

7. UV Spectra

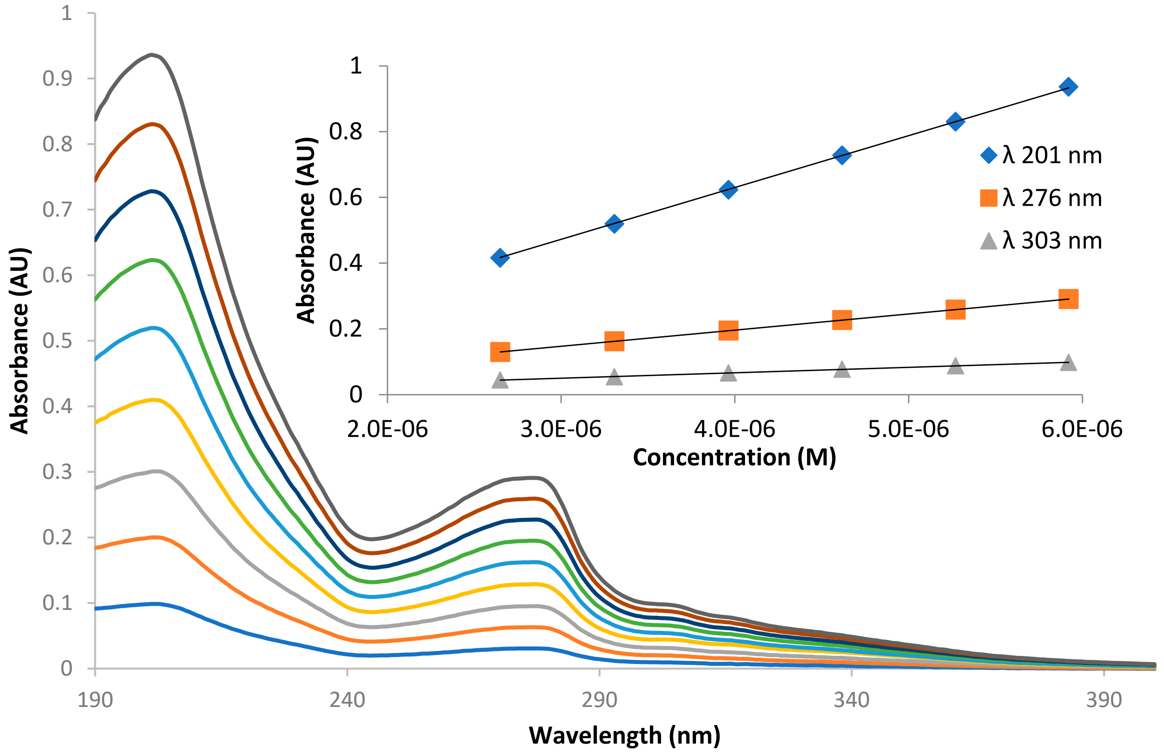

Figure S31. The UV spectrum of a replicate of [Pt(Phen)(SSDACH)(Indomethacin)(OH)]$(NO_3)_2$ (**1**) in water.

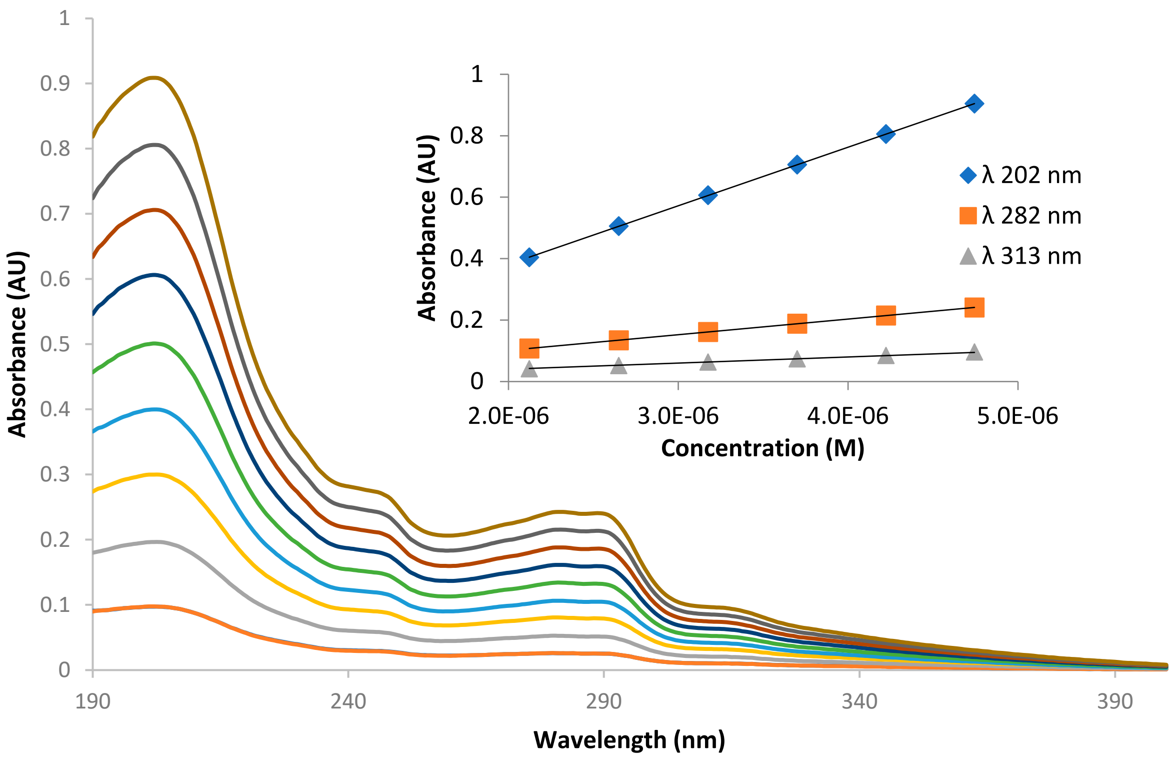

Figure S32. The UV spectrum of a replicate of [Pt(56Me$_2$Phen)(SSDACH)(Indomethacin)(OH)](NO_3)$_2$ (**2**) in water.

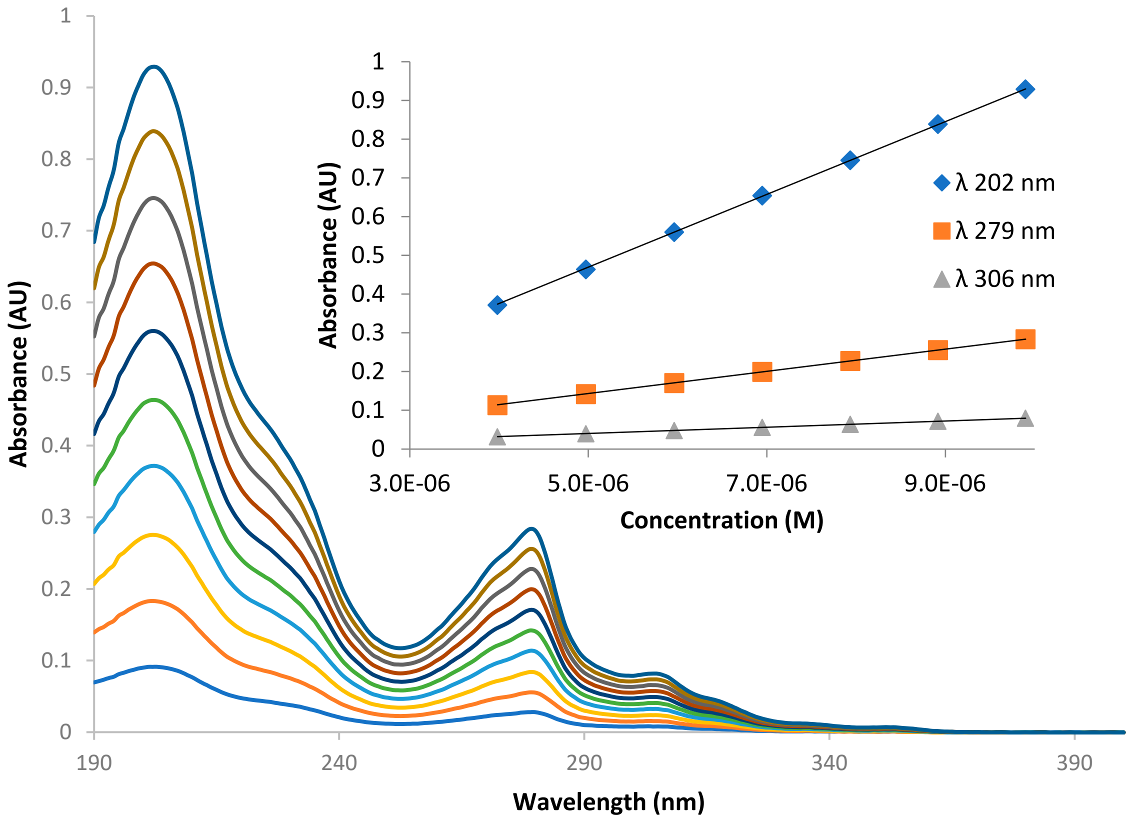

Figure S33. The UV spectrum of a replicate of [Pt(Phen)(SSDACH)(Aspirin)(OH)](NO_3)$_2$ (**3**) in water.

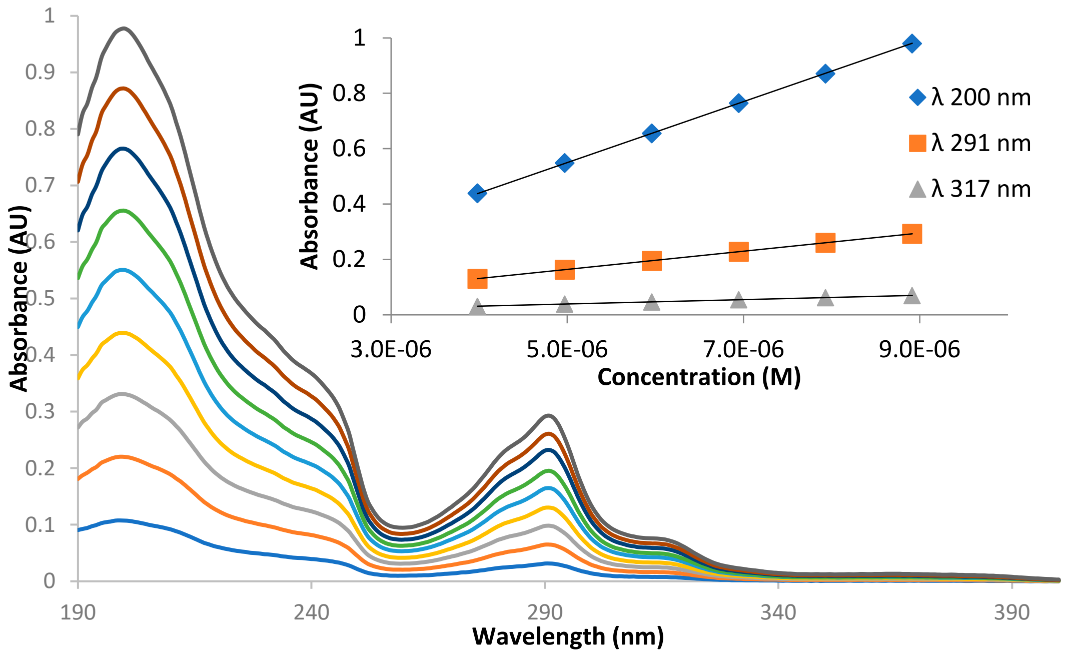

Figure S34. The UV spectrum of a replicate of [Pt(56Me$_2$Phen)(SSDACH)(Aspirin)(OH)](NO$_3$)$_2$ (**4**) in water.

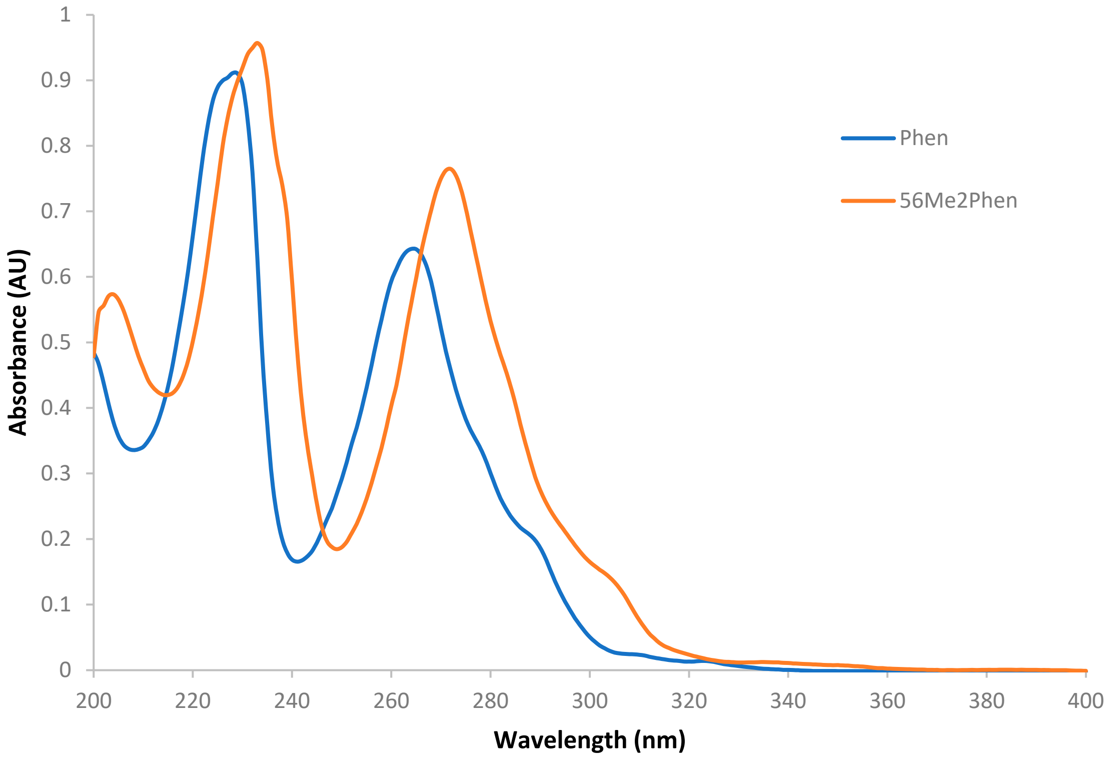

Figure S35. The UV spectra of Phen (in water) and 56Me$_2$Phen (in MeOH).

8. CD and SRCD Spectra

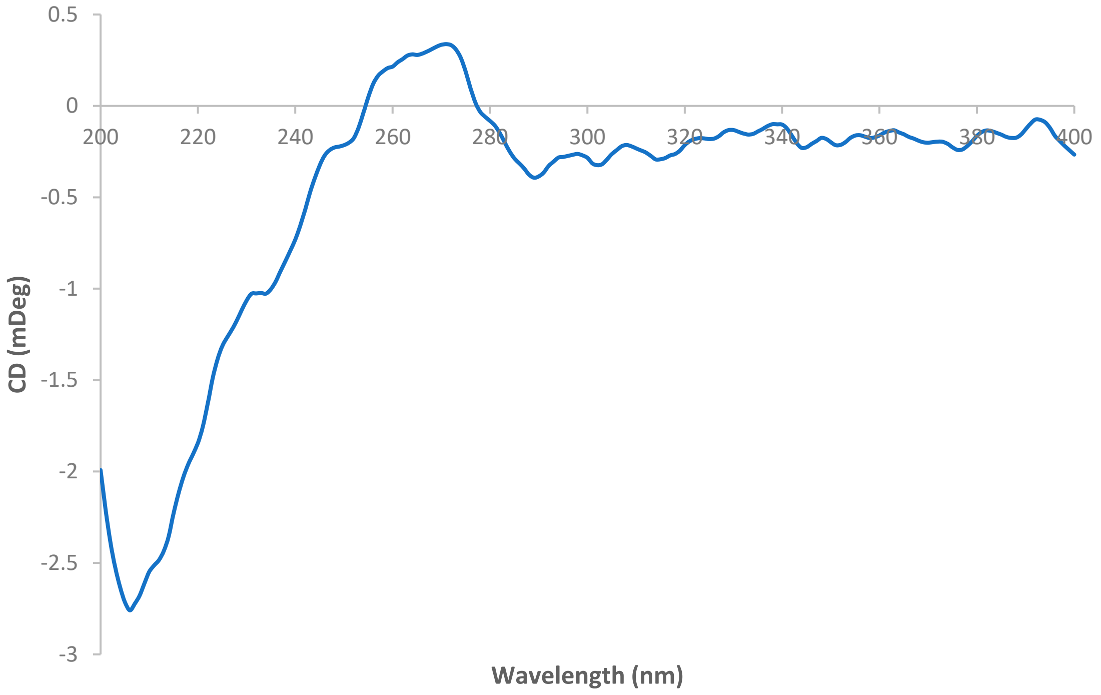

Figure S36. The CD spectrum of [Pt(Phen)(SSDACH)(Indomethacin)(OH)]$(NO_3)_2$ (**1**) in water. 9pt smoothing applied.

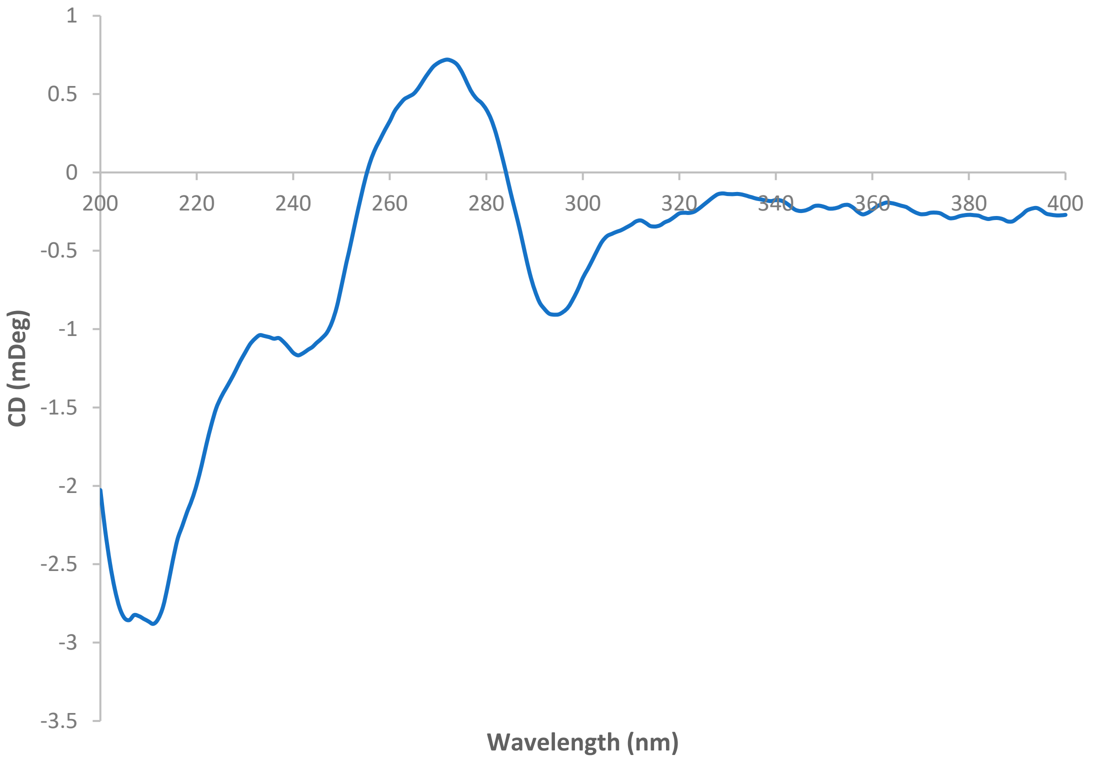

Figure S37. The CD spectrum of [Pt(56Me$_2$Phen)(SSDACH)(Indomethacin)(OH)]$(NO_3)_2$ (**2**) in water. 9pt smoothing applied.

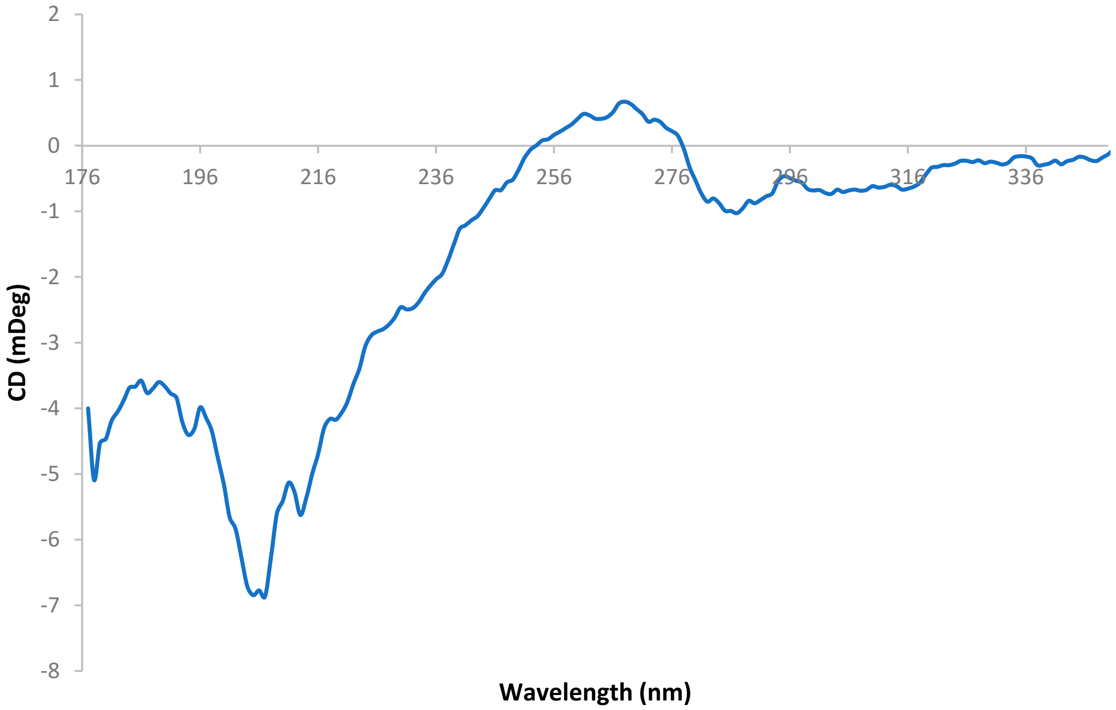

Figure S38. The SRCD spectrum of [Pt(Phen)(SSDACH)(Indomethacin)(OH)]$(NO_3)_2$ (**1**) in water. 7pt smoothing applied.

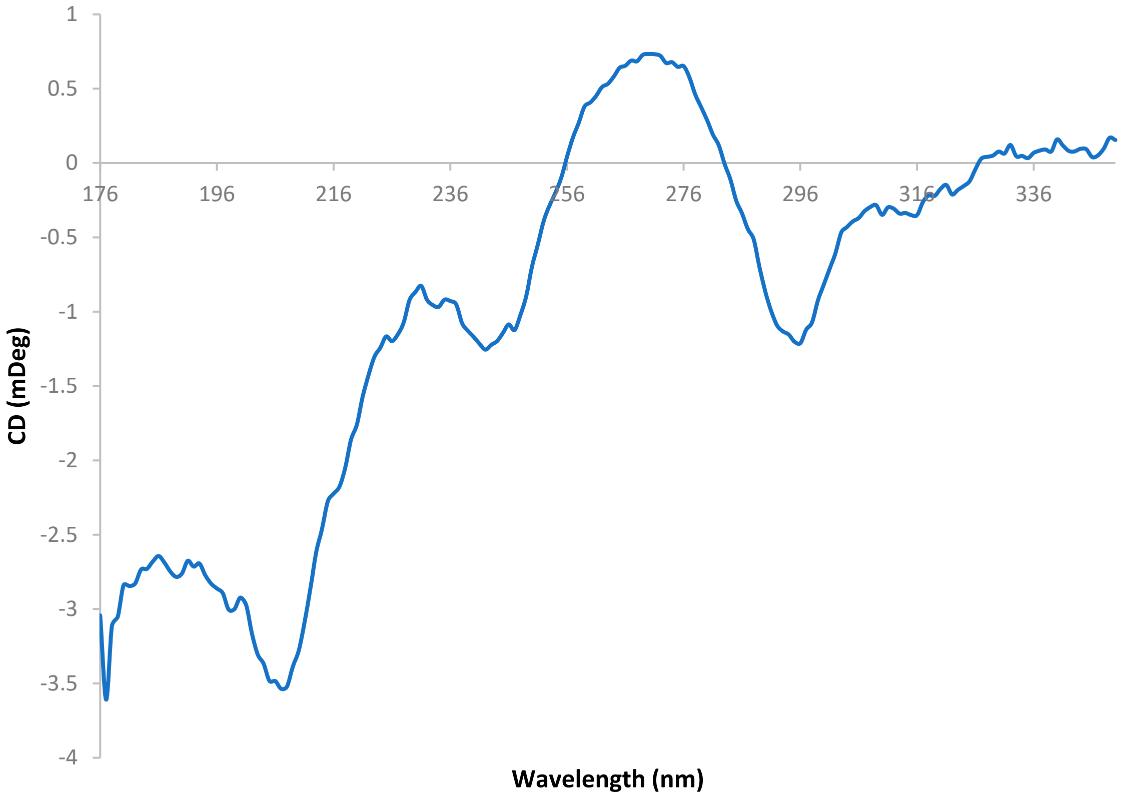

Figure S39. The SRCD spectrum of [Pt(56Me2Phen)(SSDACH)(Indomethacin)(OH)]$(NO_3)_2$ (**2**) in water. 7pt smoothing applied.

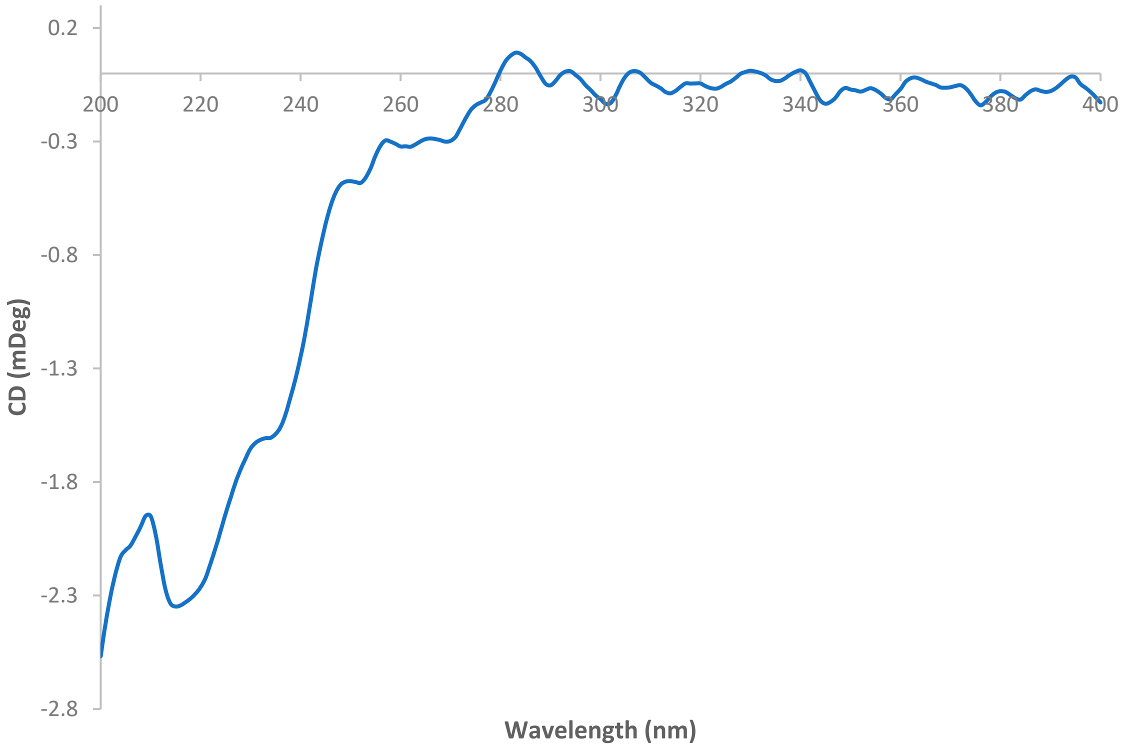

Figure S40. The CD spectrum of [Pt(Phen)(SSDACH)(Aspirin)(OH)]$(NO_3)_2$ (**3**) in water. 9pt smoothing applied.

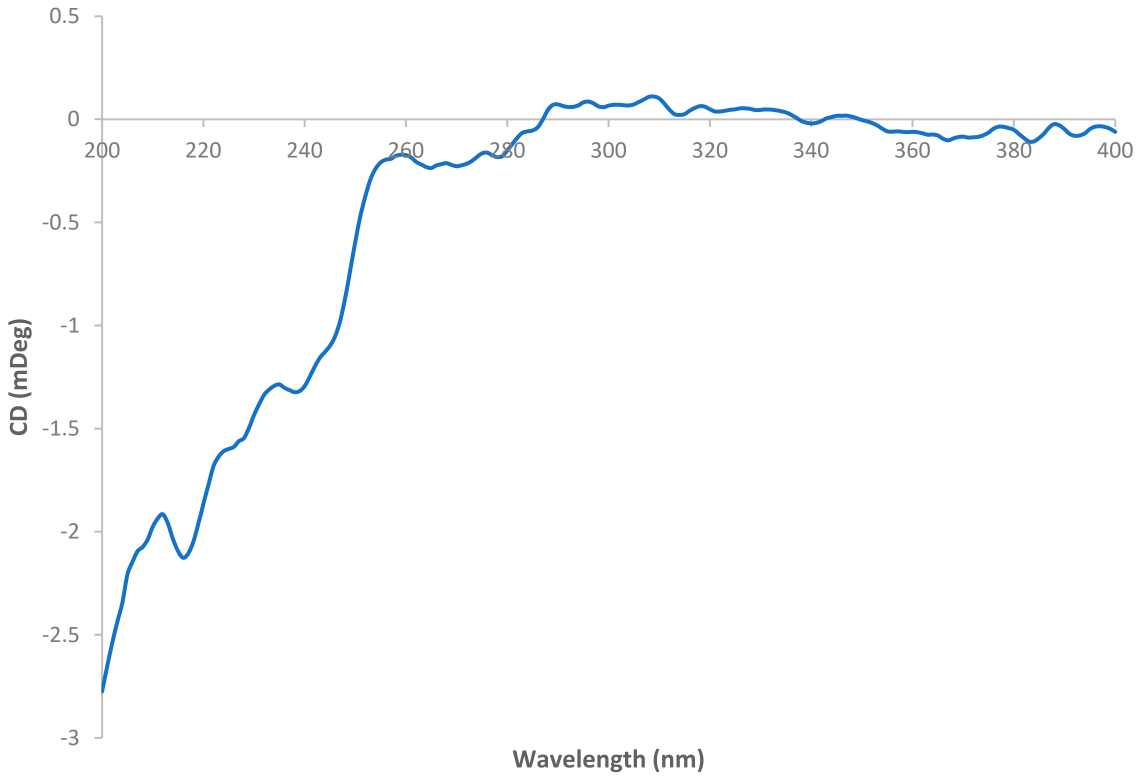

Figure S41. The CD spectrum of [Pt(56Me_2Phen)(SSDACH)(Aspirin)(OH)]$(NO_3)_2$ (**4**) in water. 9pt smoothing applied.

9. Reduction Studies

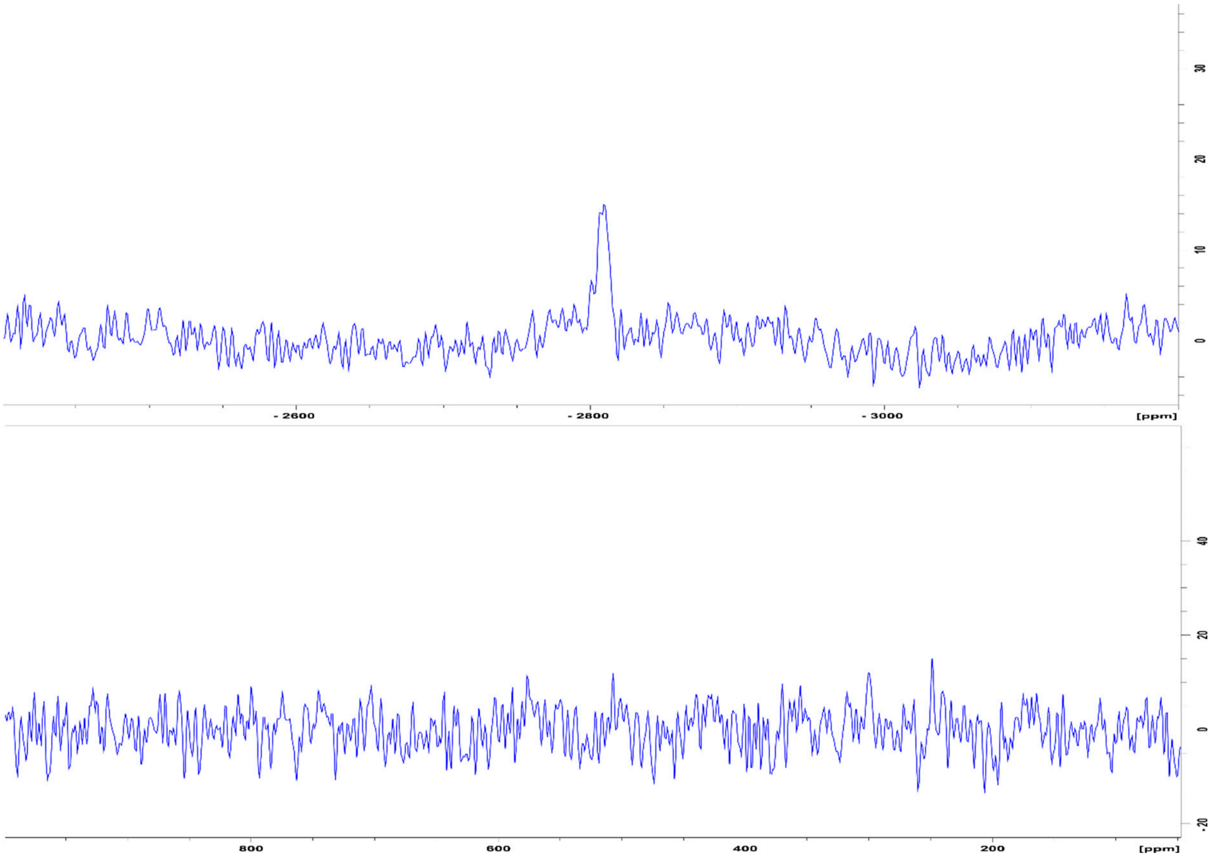

Figure S42. The ^{195}Pt NMR spectra of **3** with 10X PBS in D_2O after being reduced with ascorbic acid, showing the new peak that developed in the platinum(II) region (top) and the disappearance of the peak in the platinum(IV) region (bottom).

10. Lipophilicity Studies

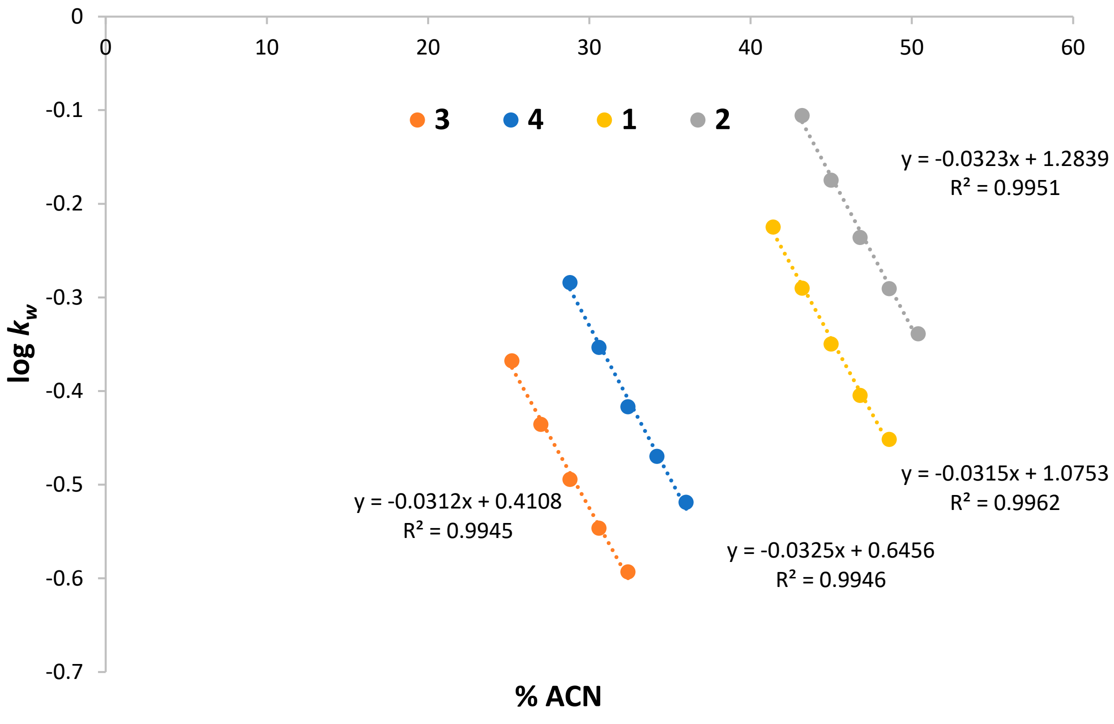

Figure S43. Plot of the concentration of organic solvent vs. $\log k_w$ for **1–4**.

11. *In vitro* Cytotoxicity Studies

Table S4. The *in vitro* cytotoxicity values of complexes **1**–**4**, their free ligands, platinum(II) and platinum(IV) di-hydroxido precursors, cisplatin, carboplatin and oxaliplatin. GI_{50} values (nM) are reported with standard error of the mean; produced from experiments that were conducted on three separate occasions (n = 3); n.d. = not determined.

	GI_{50} ± SEM (nM)											
**Compound**	**HT29**	**U87**	**MCF-7**	**A2780**	**H460**	**A431**	**Du145**	**BE2-C**	**SJ-G2**	**MIA**	**MCF10A**	**ADDP**
**Indomethacin**	>50000	>50000	>50000	5700 ± 800	>50000	>50000	>50000	>50000	>50000	>50000	>50000	>50000
**Aspirin**	>50000	>50000	>50000	>50000	>50000	>50000	>50000	>50000	>50000	>50000	>50000	>50000
**1**	83 ± 40	1300 ± 270	740 ± 230	390 ± 87	370 ± 55	400 ± 84	160 ± 54	n.d.	970 ± 290	330 ± 55	340 ± 100	350 ± 81
**2**	13 ± 3.6	47 ± 4.9	37 ± 2.3	34 ± 8.6	22 ± 4.2	40 ± 14	7.5 ± 3.6	130 ± 41	170 ± 19	21 ± 2	23 ± 8.2	24 ± 6.1
**3**	140 ± 10	600 ± 60	390 ± 80	260 ± 30	350 ± 20	670 ± 100	140 ± 10	620 ± 90	360 ± 60	160 ± 30	210 ± 29	200 ± 0
**4**	7 ± 1	24 ± 0	36 ± 10	22 ± 0	15 ± 1	31 ± 0	3 ± 1	98 ± 21	63 ± 6	8 ± 1	9 ± 2	12 ± 0
**PHENSS(II)**	87 ± 27	1200 ± 340	250 ± 41	290 ± 37	300 ± 32	340 ± 100	110 ± 27	390 ± 60	440 ± 55	240 ± 22	240 ± 60	230 ± 49
**56MESS(II)**	15 ± 6.2	76 ± 8.7	33 ± 12	39 ± 7.7	28 ± 4.7	36 ± 11	10 ± 1.3	86 ± 3	150 ± 6.7	22 ± 0.88	25 ± 10	27 ± 4.1
**PHENSS(IV)** [1]	710 ± 300	4900 ± 610	16000 ± 4500	800 ± 84	1700 ± 200	4300 ± 530	310 ± 92	3000 ± 530	1700 ± 350	3400 ± 2200	1700 ± 200	1300 ± 350
**56MESS(IV)**	36 ± 7	190 ± 23	480 ± 140	56 ± 7.1	190 ± 150	120 ± 22	15 ± 2.6	240 ± 22	210 ± 45	43 ± 2.5	61 ± 7.3	170 ± 120
**Cisplatin** [2]	11300 ± 1900	3800 ± 1100	6500 ± 800	1000 ± 100	900 ± 200	2400 ± 300	1200 ± 100	1900 ± 200	400 ± 100	7500 ± 1300	5200 ± 520	28000 ± 1700
**Oxaliplatin** [2]	900 ± 200	1800 ± 200	500 ± 100	160 ± 0	1600 ± 100	4100 ± 500	2900 ± 400	900 ± 200	3000 ± 1200	900 ± 200	n.d.	822 ± 130
**Carboplatin** [2]	>50000	>50000	>50000	9200 ± 2900	14000 ± 1000	24300 ± 2200	14700 ± 1200	18700 ± 1200	5700 ± 200	>50000	>50000	>50000

12. COX Inhibition Assay

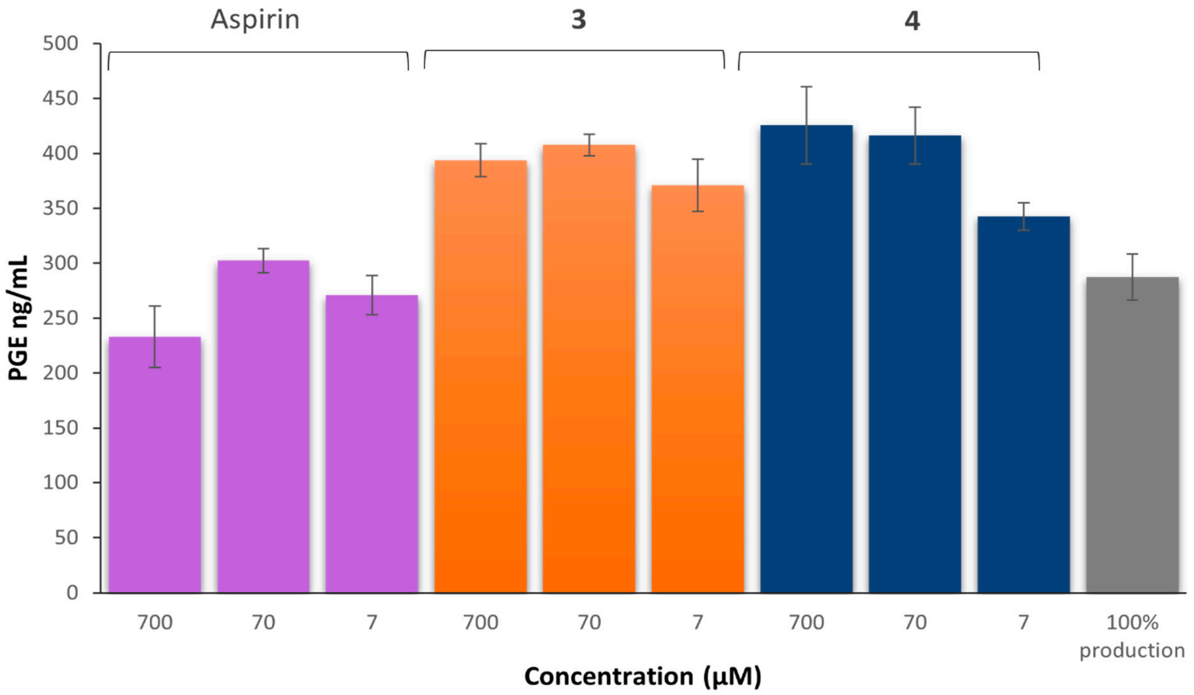

Figure S44. Inhibition of human COX- 2 by aspirin, **3** and **4**. Error bars are the average of triplicates from one experiment.

13. Platinum Uptake Studies

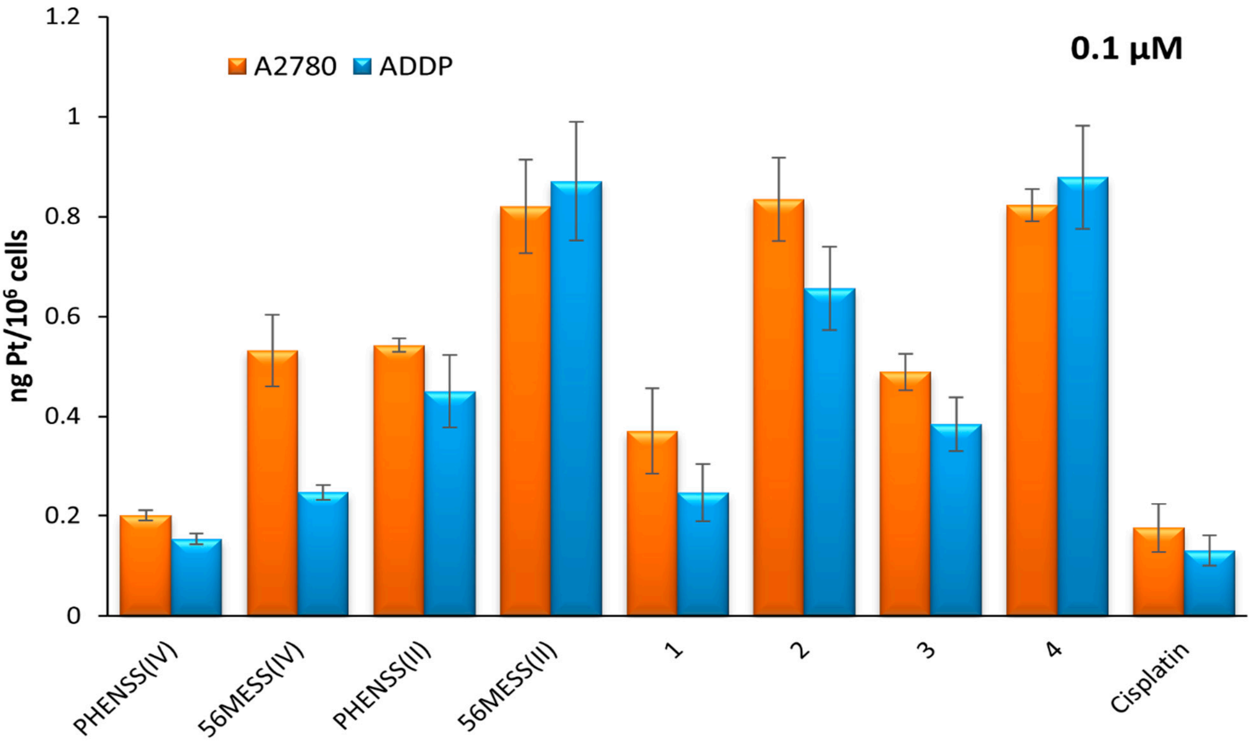

Figure S45. Cellular accumulation levels of **1–4**, their platinum(II) and platinum(IV) precursors and cisplatin against A2780 (ovarian) and ADDP (cisplatin-resistant ovarian) cancer cells that were treated with 0.1 µM for 4 hours. Values are reported in ng Pt/10^6 cells and are reported with standard error of the mean; produced from three independent experiments.

References

1. Deo, K. M.; Sakoff, J.; Gilbert, J.; Zhang, Y.; Wright, J. R. A., Synthesis, characterisation and potent cytotoxicity of unconventional platinum (IV) complexes with modified lipophilicity. *Dalton Trans.* **2019**, *48*, 17217–17227.
2. Macias, F. J.; Deo, K. M.; Wormell, P.; Clegg, J. K.; Zhang, Y.; Li, F.; Zheng, G.; Sakoff, J.; Gilbert, J.; Aldrich-Wright, J. R., Synthesis and analysis of the structure, diffusion and cytotoxicity of heterocyclic platinum (IV) complexes. *Eur. J. Chem.* **2015**, *21*, 16990–17001.
